# A little goes a long way: Weak vaccine transmission facilitates oral vaccination campaigns against zoonotic pathogens

**DOI:** 10.1371/journal.pntd.0007251

**Published:** 2019-03-08

**Authors:** Andrew J. Basinski, Scott L. Nuismer, Christopher H. Remien

**Affiliations:** 1 Department of Mathematics. University of Idaho, Moscow, Idaho, United States of America; 2 Department of Biological Sciences. University of Idaho, Moscow, Idaho, United States of America; George Washington University School of Medicine and Health Sciences, UNITED STATES

## Abstract

Zoonotic pathogens such as Ebola and rabies pose a major health risk to humans. One proven approach to minimizing the impact of a pathogen relies on reducing its prevalence within animal reservoir populations using mass vaccination. However, two major challenges remain for vaccination programs that target free-ranging animal populations. First, limited or challenging access to wild hosts, and second, expenses associated with purchasing and distributing the vaccine. Together, these challenges constrain a campaign’s ability to maintain adequate levels of immunity in the host population for an extended period of time. Transmissible vaccines could lessen these constraints, improving our ability to both establish and maintain herd immunity in free-ranging animal populations. Because the extent to which vaccine transmission could augment current wildlife vaccination campaigns is unknown, we develop and parameterize a mathematical model that describes long-term mass vaccination campaigns in the US that target rabies in wildlife. The model is used to investigate the ability of a weakly transmissible vaccine to (1) increase vaccine coverage in campaigns that fail to immunize at levels required for herd immunity, and (2) decrease the expense of campaigns that achieve herd immunity. When parameterized to efforts that target rabies in raccoons using vaccine baits, our model indicates that, with current vaccination efforts, a vaccine that transmits to even one additional host per vaccinated individual could sufficiently augment US efforts to preempt the spread of the rabies virus. Higher levels of transmission are needed, however, when spatial heterogeneities associated with flight-line vaccination are incorporated into the model. In addition to augmenting deficient campaigns, our results show that weak vaccine transmission can reduce the costs of vaccination campaigns that are successful in attaining herd immunity.

## Author summary

Zoonotic pathogens pose a significant health risk to humans. Mass vaccination programs have shown promise for controlling zoonoses in reservoir populations and, in turn, lessening the health burden posed to neighboring human populations. Despite some significant successes, major logistical challenges remain for programs that seek to establish and maintain herd immunity in free-ranging animal populations. Specifically, limited host access and costs associated with vaccine distribution may hinder efforts to vaccinate a host population and preempt spillover of a zoonotic pathogen. We use mathematical models, parameterized with data from campaigns in the US that target rabies in wildlife, to illustrate how transmissible vaccines can overcome these challenges. Specifically, we find levels of vaccine transmission necessary to boost vaccination efforts that seek to preempt the spread of rabies, and also predict the cost savings that could be realized with a transmissible vaccine.

## Introduction

Zoonotic pathogens represent a global threat to human welfare. Rabies circulating in domestic dogs in Asia and Africa, for example, results in 59,000 human deaths each year [1]. Ebola, a disease that circulates in non-human primates and bats, killed over 11,000 people during the 2014 outbreak [2]. In addition to the continual threat posed by zoonotic pathogens that occasionally spill over into human populations, zoonoses function as a major source of new infectious diseases in humans [3, 4]. Over 60% of emerging infectious diseases in humans originated as zoonotic pathogens, and recent studies predict that new harmful zoonoses are most likely to originate in geographical hotspots where health infrastructure is poorest [3]. Given these global risks, the ability to vaccinate free-ranging animal populations against dangerous zoonotic pathogens remains an essential goal for safeguarding human populations against future infectious diseases.

Free-ranging animal populations present challenges to mass vaccination. The ultimate goal of any vaccination campaign is to establish herd immunity against a targeted pathogen, that is, to vaccinate a proportion of the population that is sufficient to preclude the pathogen’s spread. In the US, various free-ranging mammalian populations, including coyotes, gray fox, and raccoons, still act as potential or active reservoirs for multiple variants of the rabies virus [5]. These wildlife pose a serious health risk to humans or domestic pets that come into contact with a rabid animal. However, achieving herd immunity in these populations requires that vaccine be distributed across thousands of square kilometers [5, 6]. Because of the inaccessibility of wildlife hosts, Oral Rabies Vaccine (ORV) baits, distributed by aircraft, have been the primary means of vaccinating animal populations that are spread across large tracts of land [7, 8]. ORV bait programs have been crucial in lowering the incidence of raccoon rabies in the US and Canada, and played a fundamental role in eliminating canine rabies from difficult-to-access populations such as coyotes and foxes [9, 10].

Though proven effective in some cases, ORV programs highlight challenges that long-term wildlife vaccination campaigns must overcome. In North America, raccoons serve as the primary reservoir of the raccoon variant of the rabies virus. In order to mitigate the risk of transmission to humans, the US and Canadian governments have organized intense vaccination efforts since the 1990s, with the goal of preventing the westward spread of raccoon rabies across the Appalachian mountains, as well as the northward spread of the virus into Canada [11, 12]. However, low rates of seroconversion in raccoons, and bait competition with non-targeted hosts, together prevent vaccine coverage from exceeding the herd immunity threshold [13–15]. In turn, despite decades of ongoing vaccination effort, the rabies virus still occasionally breaches vaccination barriers meant to contain it [5, 16, 17]. For other wildlife reservoirs, such as coyotes and gray fox, ORV programs in the US are successful at establishing and maintaining herd immunity [9]. However, to ensure that the rabies virus cannot re-invade, these programs may need to be maintained for decades before the risk of rabies re-infection has passed. These challenges highlight the need for cost-effective ways to immunize populations that are difficult to access.

Transmissible vaccines are a promising new technology that, when paired with oral vaccine technology, could transform our ability to vaccinate wildlife populations. Transmissible viral vaccines are engineered to transmit between hosts, inoculating hosts they infect. Vaccine transmission supplements direct vaccination efforts and increases vaccine coverage. To date, transmissible vaccines have been explored for zoonotic pathogens such as Ebola in non-human primates [18] and Hantavirus in deer mice [19], and have been suggested as a possibility for rabies [20]. Although transmissible vaccines that target human pathogens are still in the early stages of development, a transmissible vaccine targeting myxoma and rabbit hemorrhagic fever has been both developed and tested in European rabbits. Studies of the rabbit vaccine demonstrated relatively high levels of transmission in caged rabbit populations, and in field trials, the vaccine was shown to immunize a substantial portion of a rabbit population through horizontal transmission [21, 22].

In addition to this promising empirical work, theoretical models of transmissible vaccines suggest that low levels of transmission can dramatically increase the level of vaccine coverage in a well-mixed host population [23–25]. However, little is known about the extent to which weak vaccine transmission might augment campaigns that target a geographically widespread, free-ranging animal population in which host interactions are spatially localized. The extent to which the vaccine transmits is encapsulated in the basic reproduction number, notated *R*_0,*v*_, that describes the average number of secondary vaccine infections caused by one vaccine-infected individual in a susceptible population. Weakly transmissible vaccines, defined as vaccines with *R*_0,*v*_ < 1, are particularly desirable as they have a reduced likelihood of vaccine evolution, which reduces the risk of vaccine reversion, as well as competition between the vector and vaccine [23, 26].

We use a mathematical modeling framework, based on the SIR (Susceptible-Infected-Recovered) infection model, to quantify the benefits imparted by vaccine transmission on long-term ORV-style vaccination campaigns that target wildlife in the US. Our focal questions are: (1) can weak levels of vaccine transmission augment campaigns in the US that fail to establish herd immunity in raccoon populations? (2) to what extent can vaccine transmission reduce the costs of maintaining herd immunity in ORV programs that are successful? We address these questions using mathematical models parameterized with data from historical campaigns that targeted raccoons, coyotes and gray fox in the US.

## Materials and methods

We model a population of animal hosts that are regularly vaccinated with a transmissible vaccine bait to preempt the establishment of rabies. We assume that the rabies virus has not yet infected the host population, so any immunity that exists in the population is a result of vaccination. The modeling framework uses differential equations to model the effects of host demography, vaccine transmission, and attributes of the vaccination campaign to predict the fraction of the population that is vaccinated at steady state (i.e. seroprevalence). We use two versions of a single underlying mathematical model. The first model ignores any spatial heterogeneities that might exist in the distribution of immune hosts as a result of vaccination. The second model incorporates the spatial challenges associated with vaccination programs that distribute vaccine along lines in the environment (i.e. flight-lines).

### Modeling framework

We start with a model that describes a well-mixed host population. The model tracks the densities of hosts that are susceptible to rabies infection (*S*), hosts that are currently infected with a transmissible vaccine (*I*_*v*_), and hosts that have recovered from vaccine infection (*V*). In the model, new susceptible hosts are born at constant rate *b*, and all hosts die at per-capita rate *d*. Vaccination of susceptible hosts occurs in one of two ways. The first is through direct consumption of a vaccine bait containing a transmissible vaccine, which occurs with per-capita rate *σ*. Upon consumption of the bait, susceptible hosts become infected with the vaccine virus. We assume that, simultaneously, exposure to the rabies antigen that the vaccine carries prompts a host immune response that results in lifelong immunity to the rabies virus. Alternatively, susceptible hosts can become vaccinated through infectious contact with another host that is infected with the vaccine. The rate at which such contacts occur will depend on attributes of the vector virus from which the vaccine is made and the rate at which hosts experience infectious contact with each other. We assume that vaccine-infected hosts transmit the vaccine to susceptible hosts at frequency-dependent rate $\frac{\beta_{v}S\left(t\right)\;I_{v}\left(t\right)}{S\left(t\right)+I_{v}\left(t\right)+V\left(t\right)}$. Vaccine-infected hosts clear the infection at per-capita rate *δ*_*v*_, and transition into a vaccine-recovered class (*V*). After recovering from infection with the vaccine, the host is immune to subsequent vaccine infection, as well as infection with the rabies virus. These biological assumptions lead to the following system of differential equations:

$$\begin{array}{c} \begin{array}{cc} \frac{dS}{dt} & =b-\sigma S-dS-\frac{\beta_{v}SI_{v}}{S+I_{v}+V}\\ \frac{dI_{v}}{dt} & =\sigma S-\left(d+\delta_{v}\right)I_{v}+\frac{\beta_{v}SI_{v}}{S+I_{v}+V}\\ \frac{dV}{dt} & =\delta_{v}I_{v}-dV \end{array} \end{array}$$ (1)

Many ongoing rabies campaigns utilize aircraft or cars to distribute vaccines into geographically widespread wildlife populations. In these scenarios, the vaccine is distributed along lines in the environment. In order to ensure an even distribution of vaccines, the flight-line spacing must be chosen with the home range of the host animal in mind [27]. Choosing a flight-line spacing that is too large relative the animal’s home range, for example, will cause gaps in seroprevalence between flight-lines. We modify System (1) to investigate how vaccine transmission addresses these unique spatial challenges associated with flight-line vaccination. The resulting model tracks the same classes as System (1), however each state variable is a one-dimensional spatial density described by a partial differential equation. For each host class, we use a diffusion term with diffusion coefficient *k* to model the movement of a host throughout its lifetime (S1 Appendix). In the model, flight-lines are spaced at intervals of width 2*L*, and the vaccination rate *σ* is normally distributed around flight-line positions according to 2*Lf*(*x*)*σ*. Here, *f*(*x*) is a normal distribution that is truncated to the interval [−*L*, *L*] with standard deviation *ξ*; the factor 2*L* ensures that the mean density of vaccine effort is independent of the flight-line spacing that is chosen (more details in S2 Appendix). Now, vaccine infection is a spatially localized process, so that an infected host at location *x* can only infect susceptible hosts that are also at location *x*. The resulting system is

$$\begin{array}{c} \begin{array}{cc} \frac{\partial S}{\partial t} & =k\frac{\partial^{2}S}{\partial x^{2}}+b-2Lf\left(x\right)\sigma S-dS-\frac{\beta_{v}SI_{v}}{S+I_{v}+V}\\ \frac{\partial I_{v}}{\partial t} & =k\frac{\partial^{2}I_{v}}{\partial x^{2}}+2Lf\left(x\right)\sigma S-\left(d+\delta_{v}\right)I_{v}+\frac{\beta_{v}SI_{v}}{S+I_{v}+V}\\ \frac{\partial V}{\partial t} & =k\frac{\partial^{2}V}{\partial x^{2}}+\delta_{v}I_{v}-dV \end{array} \end{array}$$ (2)

We also use variations of Systems (1) and (2) to model campaigns that use a nontransmissible vaccine. For these simulations, the *I*_*v*_ class is omitted, *β*_*v*_ is set to 0, and directly vaccinated susceptible hosts transition into the *V* class.

### Data

We use data from the USDA to parameterize our models. Each year, the USDA compiles a “National Rabies Management Summary Report” that provides an overview of the previous year’s vaccination efforts, including where vaccination campaigns were carried out, types of wildlife that are vaccinated, and the number of vaccine baits used. In addition, these reports document the seroprevalence that was measured in follow-up population surveys. All data were retrieved from summary reports posted on the USDA website for the years 2006–2010 [28].

### Vaccination campaigns without continual vaccination

If campaigns occur only rarely, the effective vaccination rate *σ* is zero, and the host population relies on vaccine transmission to distribute the vaccine. In this case, our nonspatial model reduces to a classic SIR infection model. Local stability analysis of our model indicates that if a small number of vaccine-infected individuals are introduced into an otherwise susceptible population, the density of seropositive hosts will increase when *R*_0,*v*_ > 1, and comprise a fraction

$$\phi =1-\frac{1}{R_{0,v}}$$ (3)

of the host population at steady state (S3 Appendix). Here, *R*_0,*v*_ is the so-called basic reproduction number of the vaccine, defined as the number of secondary vaccine infections caused by one infected individual in an otherwise susceptible population ($R_{0,v}=\frac{\beta_{v}}{d+\delta_{v}}$, parameters defined in Table 1). Eq (3) implies that, if the goal of a campaign is to maintain seroprevalence in the host population at a level *ϕ*, the vaccine used must transmit at a level

$$R_{0,v}=\frac{1}{1-\phi }.$$ (4)

**Table 1:** Description of state variables and model parameters.

Name	Description
*S*	State variable that tracks density of susceptible hosts
*I*_*v*_	State variable that tracks density of vaccine-infected hosts
*V*	State variable that tracks density of hosts recovered from vaccine infection
*b*	Birth rate
*d*	Death rate
*σ*	Vaccination rate
*β*_*v*_	Vaccine transmission coefficient
*δ*_*v*_	Vaccine recovery rate
*k*	Diffusion coefficient
*L*	One-half flight-line spacing
*ξ*	Standard deviation of vaccine distribution (see text)
*C*_*f*_	Per km cost of flight-line vaccination
*C*_*b*_	Cost per vaccine bait

Time units are years (yr), spatial units are kilometers (km). Both vaccine-infected and vaccine-recovered individuals are assumed to be immune to rabies infection. More information on parameter choices can be found in the S1 Appendix.

### Augmenting regular vaccination in a homogeneous population

To understand the extent to which vaccine transmission can augment long-term campaigns when regular vaccination is possible, we find steady states of System (1) with *σ* > 0. Stability analysis indicates that with constant vaccination, the seroprevalence of System (1) approaches a level *ϕ* described by the expression

$$\phi =\frac{d\left(1-R_{0,v}\right)-\sigma +\sqrt{{\left(dR_{0,v}+d+\sigma \right)}^{2}-4d^{2}R_{0,v}}}{2dR_{0,v}}$$ (5)

(S3 Appendix). Eq (5) shows that the long-term effect of vaccine transmission on seroprevalence is again encapsulated in the vaccine’s *R*_0,*v*_. Furthermore, for a fixed value of *R*_0,*v*_, the steady state benefit from transmission does not depend on the length of time over which these secondary infections occur, which is given by $\frac{1}{\delta_{v}}$.

To find the level of vaccine transmission that is necessary to augment real-world campaigns, we parameterize *σ* in Eq (5) to a range of seroprevalence outcomes from USDA vaccination campaigns applied to raccoons. Between 2006–2010, follow-up seroprevalence surveys reported average seroprevalence that varied from a minimum of 0.29 in 2006, to a high of 0.37 in 2010. Interpreted as steady state seroprevalence levels, and assuming that raccoons live for 2.5 years, these values of *ϕ* imply a range of vaccination rates 0.17 < *σ* < 0.24 yr^−1^(S1 Appendix).

### Augmenting seroprevalence in a nonhomogeneous population

We use our spatial model to understand how heterogeneities in vaccine distribution affect the benefits of a transmissible vaccine. To this end, we numerically solve for steady state solutions of System (2) on the interval [−*L*, *L*], with Neumann boundary conditions that describe the aggregate effects of many repeating flight-lines. We simulate high and low values of spatial heterogeneity in the distribution of vaccines by adjusting *ξ*, and we use values of the diffusion coefficient *k* to simulate small (1 km^2^) and large (10 km^2^) host home ranges. This variability in home range is chosen to reflect the variability that is found in raccoons in peri-urban and rural environments (details in S1 Appendix).

We nondimensionalize System (2) to better understand the potential for vaccine transmission to smooth spatial heterogeneities in population seroprevalence. Nondimensionalization is an analytical technique that summarizes the effects of a model’s parameters into unitless parameter combinations (S2 Appendix). Our analyses show that spatial heterogeneities are encapsulated in two nondimensional parameters. $\hat{\xi }$ describes the level of spatial heterogeneity in the distribution of vaccination effort around each flight-line location, scaled relative to one-half of the flight-line spacing. *κ* is referred to as scaled dispersal, and describes the capacity for spatial heterogeneities in seroprevalence to persist as a function of host home range, the duration of vaccine infection, and the spacing of flight-lines in the environment:

$$\begin{array}{ccc} \kappa =\frac{k}{\left(d+\delta_{v}\right)L^{2}} & & \hat{\xi }=\frac{\xi }{L} \end{array}$$ (6)

Motivated by transmissible vaccine designs with a long duration of infection, we investigate how vaccines with slow recovery rates (i.e. small *δ*_*v*_) might augment the spatial lows that are predicted by our model. We parameterize our model to the yearly averaged seroprevalence levels that were realized in campaigns targeting raccoons. Next, we use a root-solving method to determine the minimal amount of vaccine transmission, *R*_0,*v*_, that is necessary to achieve herd immunity. In these simulations, we consider a population protected from rabies when the minimum of the spatial seroprevalence is raised to the herd immunity threshold *ϕ* = 0.5 (details in S2 Appendix). All numerical analysis is performed in the statistical language R [29].

### Cost reduction in homogeneous populations

In populations where a traditional oral vaccination campaign can achieve herd immunity (e.g., coyotes and gray fox), the use of a weakly transmissible vaccine could result in large reductions in program costs. To quantify the savings that might be realized by using a transmissible vaccine, we use the spatially homogeneous model, described by System (1), to find the fractional reduction in the rate of vaccination that is required to sustain herd immunity at level *ϕ* in a host population. In doing so, we use the fact that a fractional reduction in the vaccination rate is equivalent to a fractional reduction in the rate at which vaccine baits must be deposited (S2 Appendix). Furthermore, if bait depletion by other animals can be ignored, a continual vaccination rate *σ* relates to the number of vaccines distributed per year, *ρ*, by

$$\sigma =\rho {\left(\frac{b}{d}\right)}^{-1}.$$ (7)

Here, $\frac{b}{d}$ is the steady state density of the host population.

If a nontransmissible vaccine is used to maintain seroprevalence at level *ϕ*, the rate of vaccination must exceed

$$\sigma_{NT}^{*}=d\frac{\phi }{1-\phi }$$ (8)

By solving Eq (5) for *σ*, we find that a transmissible vaccine can achieve the same seroprevalence with

$$\sigma_{T}^{*}=d\frac{\phi }{1-\phi }\left(1-R_{0,v}\left(1-\phi \right)\right)$$ (9)

(S3 Appendix). With Eqs (8) and (9), we calculate the fractional reduction in the rate of vaccination that is required for sustained herd immunity,

$$f_{\sigma }=1-\frac{\sigma_{T}^{*}}{\sigma_{NT}^{*}}=R_{0,v}\left(1-\phi \right).$$ (10)

Note that the population density $\frac{b}{d}$ is not present in the fractional reduction calculation, and need not be estimated.

We use Eq (10) to calculate the theoretical reduction in bait costs that would have been possible in past campaigns if a transmissible vaccine with *R*_0,*v*_ = 0.9 was used. To parameterize *ϕ*, we use seroprevalence outcomes in campaigns that targeted coyotes and gray fox between 2006–2010. Next, we multiply the calculated reductions by the total number of vaccine baits that were used, and the cost per vaccine bait. For this calculation, we assume that the per-unit cost of the transmissible vaccine bait is the same as a nontransmissible bait, and later evaluate how the anticipated savings might differ if the transmissible vaccine is more expensive. Accounting for inflation, and using vaccine bait costs that were reported for similar campaigns [30], we estimate a current value of $2.12 per bait (details in S1 Appendix).

### Cost reduction in a nonhomogeneous population

In addition to the expenses associated with the number of vaccine baits that are required, campaigns must also acquire, maintain, and man aircraft that distribute baits. To better understand the cost reductions that are possible in such programs, we define a function that incorporates both the expenses from the use of aircraft (e.g. wages, maintenance, fuel), and the purchase of vaccine baits. To this end, we assume the vaccinated region *A* is an *ℓ* × *w* km^2^ rectangle. Given that flight-lines are arranged along either the *ℓ* or *w* direction and spaced at intervals of 2*L*, the linear flight distance required to vaccinate the region *A* grows according to $\frac{A}{2L}$ km. Defining *C*_*f*_ as the cost per linear kilometer of flight, the total flight costs of vaccinating the area *A* scale with flight-line spacing as $C_{f}\frac{A}{2L}$. The expenses from the purchase of vaccine baits are given by the product $C_{b}\;\sigma \left(\frac{b}{d}\right)\;A$, where *C*_*b*_ is the cost per bait, and $\sigma (\frac{b}{d})\;A$ is the number of vaccine baits required per year to achieve an effective vaccination rate *σ* when population density is $\frac{b}{d}$ (S2 Appendix). Combining flight and bait costs, and dividing by the area of region *A* gives a per km^2^ cost of

$$C=C_{f}\frac{1}{2L}+C_{b}\frac{b}{d}\sigma .$$ (11)

To estimate the cost reduction that is possible in flight-line vaccination campaigns, we use a numerical solver to find the pairing of vaccination rate σ*, and flight-line spacing 2L* km, that minimizes Eq (11) while maintaining seroprevalence at level *ϕ* = 0.5. To convert the optimal strategy into a dollar amount, we use the same baseline vaccine bait cost as before (*C*_*b*_ = 2.12), and a flight-line cost of *C*_*f*_ = 18.16 km^−1^. We vary *C*_*b*_ to better understand how sensitive the cost reductions are to the cost markups that might apply to transmissible vaccines. The value of *C*_*f*_ is derived using averaged flight costs reported for campaigns in Ohio, and multiplying by the standard flight-line spacing (0.5 km) to convert to cost per linear kilometer of flight (S1 Appendix). We choose host densities of $\frac{b}{d}=1,10,100$ km^−2^ to simulate the wide range of densities found in raccoons.

### Sensitivity analysis of cost reduction

In order to gauge the sensitivity in the cost reductions that are predicted by our model, we also calculate the cost reductions that occur when assumptions of the model are changed. The Baseline model simulates a vaccine with a 1 month infectious period, *R*_0,*v*_ = 1, and a desired seroprevalence of *ϕ* = 0.5. The “Lagged Immunity” and “Temporary Immunity” variants are obtained by changing the equations of the Baseline model. In the Lagged Immunity variant, hosts are not immune to rabies until they have fully recovered from vaccine infection. In the Temporary Immunity variant, rabies-immunity wanes after a period of one year. All other variants are obtained by changing parameter values. More details can be found in the S2 Appendix.

## Results

### Vaccination campaigns without continual vaccination

In the absence of repeated vaccinations, a single campaign could in theory preempt the establishment of rabies if *R*_0,*v*_ is sufficiently large. Standard epidemiological theory implies that to achieve seroprevalence *ϕ*, the vaccine must transmit at level

$$R_{0,v}=\frac{1}{1-\phi }.$$

This expression implies that 1.7 < *R*_0,*v*_ < 2.5 is required to achieve the seroprevalence that successfully preempted the reinvasion of rabies into wild canines (0.4 < *ϕ* < 0.6, [9]). Similarly, *R*_0,*v*_ ≈ 2 is required to achieve the recommended seroprevalence in raccoons (*ϕ* ≈ 0.5, [31, 32]). Because it is currently unknown whether these levels of vaccine transmission are feasible or will ever be deemed safe to implement in free-ranging animal populations, we next evaluate the extent to which vaccine transmission can augment ongoing campaigns that regularly vaccinate the host population.

### Augmenting seroprevalence in a homogeneous population

**Fig 1:**
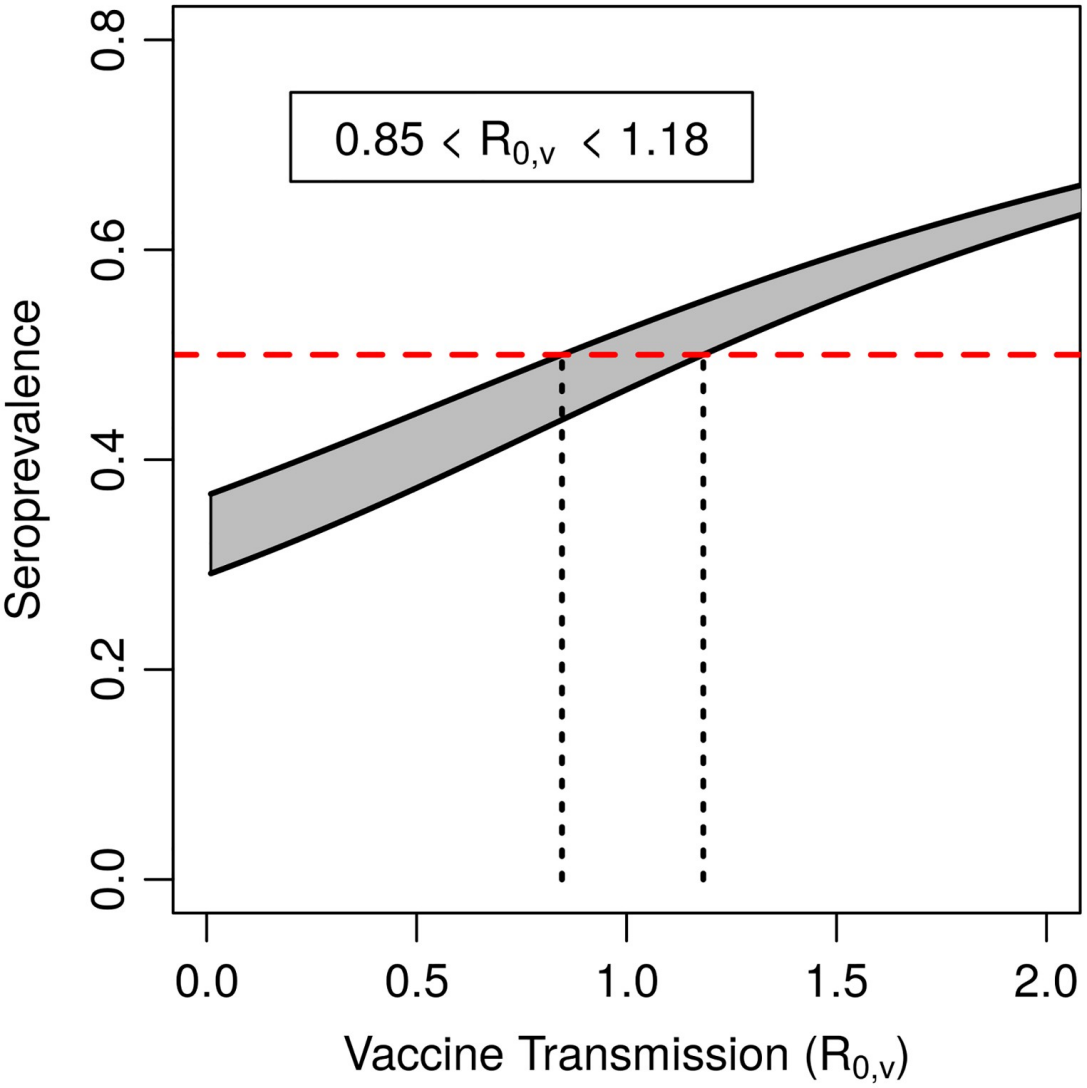
Predicted seroprevalence with vaccine transmission in US raccoon rabies campaigns. The filled region indicates the range in seroprevalence that would be achieved with a transmissible vaccine, given the range in seroprevalence that was achieved by USDA campaigns with a nontransmissible vaccine. The red dashed line indicates the coverage that is recommended to protect a raccoon population from rabies. The dashed black lines indicate the range of *R*_0,*v*_ that satisfies the herd immunity threshold. Other parameters: *d* = 0.416 yr^−1^, *δ*_*v*_ = 12 yr^−1^.

If spatial heterogeneities are ignored, our model predicts that weak vaccine transmission could be effective at augmenting US campaigns that target raccoons but do not achieve the desired herd immunity threshold of *ϕ* = 0.5. When parameterized to vaccination outcomes reported in National Rabies Management Summary Reports between 2006–2010, our model suggests that a vaccine with 0.85 < *R*_0,*v*_ < 1.18 would augment the range of seroprevalence averages to that required for herd immunity (Fig 1). This implies that even weakly transmitting vaccines, i.e. those that do not transmit sufficiently to remain endemic in the population, might substantially benefit campaigns that seek to establish herd immunity in raccoon populations.

### Augmenting seroprevalence in a nonhomogeneous population

**Fig 2:**
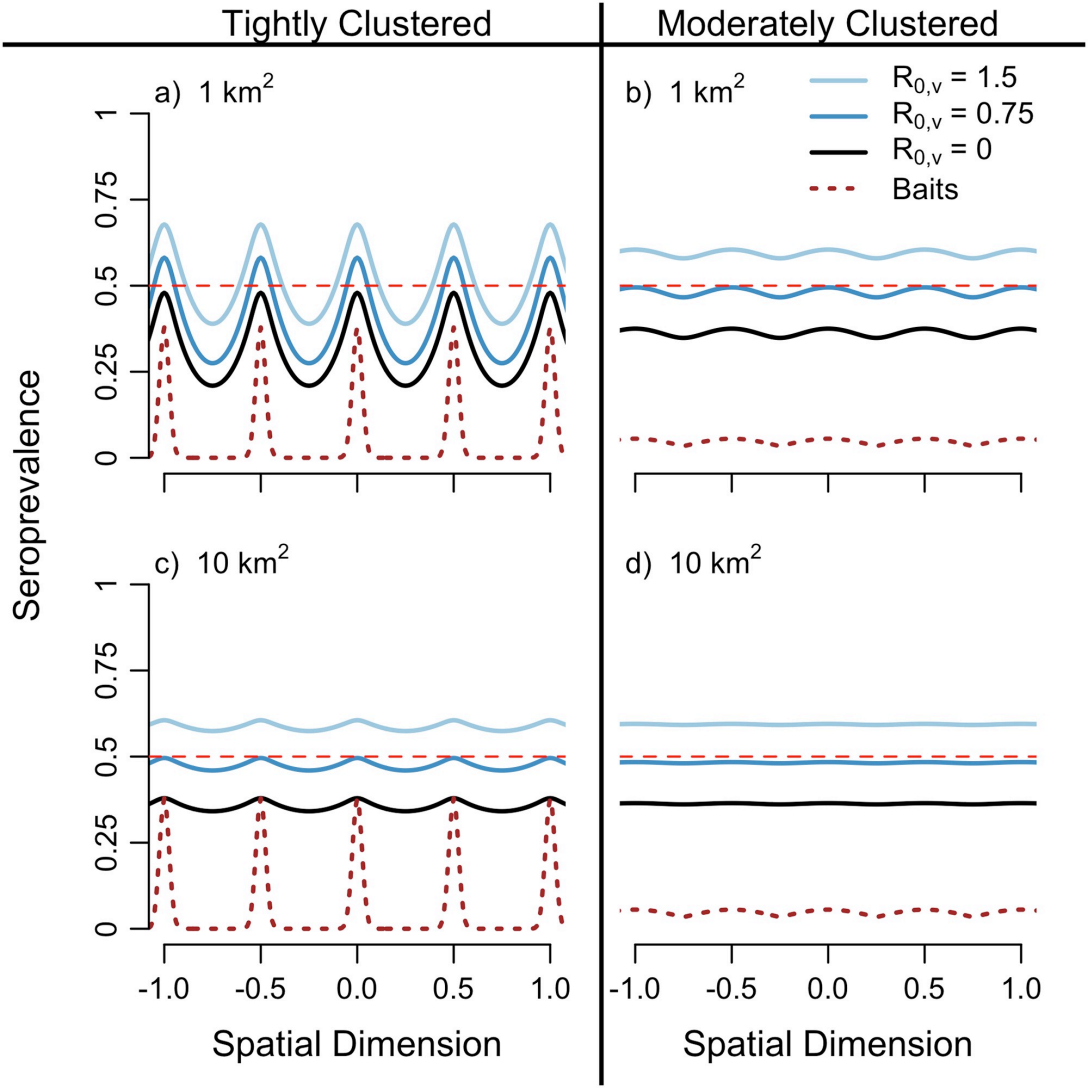
Spatially explicit seroprevalence achieved with traditional and transmissible vaccines. The host home range is set to coincide with typical home ranges of raccoons: 1 km^2^ (panes a and b, *k* = 0.01) and 10 km^2^ (panes c and d, *k* = 0.1). The vaccine bait heterogeneity is either tightly clustered (*ξ* = 0.025, left column) or moderately clustered (*ξ* = 0.25, right column). The brown dashed line depicts the steady state distribution of vaccine, scaled for visibility. Other parameters: *d* = 0.416 yr^−1^, *σ* = 0.24 yr^−1^, *δ*_*v*_ = 12 yr^−1^.

When spatial heterogeneities are incorporated, elevating the minimal seroprevalence to the herd immunity threshold can require substantially higher levels of vaccine transmission. Both host movement and vaccine bait heterogeneity influence the amount of vaccine transmission that is necessary to raise seroprevalence levels above the 0.5 herd immunity threshold (Fig 2). Our model predicts that hosts with small home ranges (∼1 km^2^) are most likely to be affected by heterogeneities in vaccine coverage when the distribution of vaccine is spatially clustered along flight-lines. In these populations, seroprevalence falls below the herd immunity threshold even when vaccine transmission is relatively high, *R*_0,*v*_ = 1.5. As a result, portions of the population remain unprotected from pathogen invasion (Fig 2).

**Fig 3:**
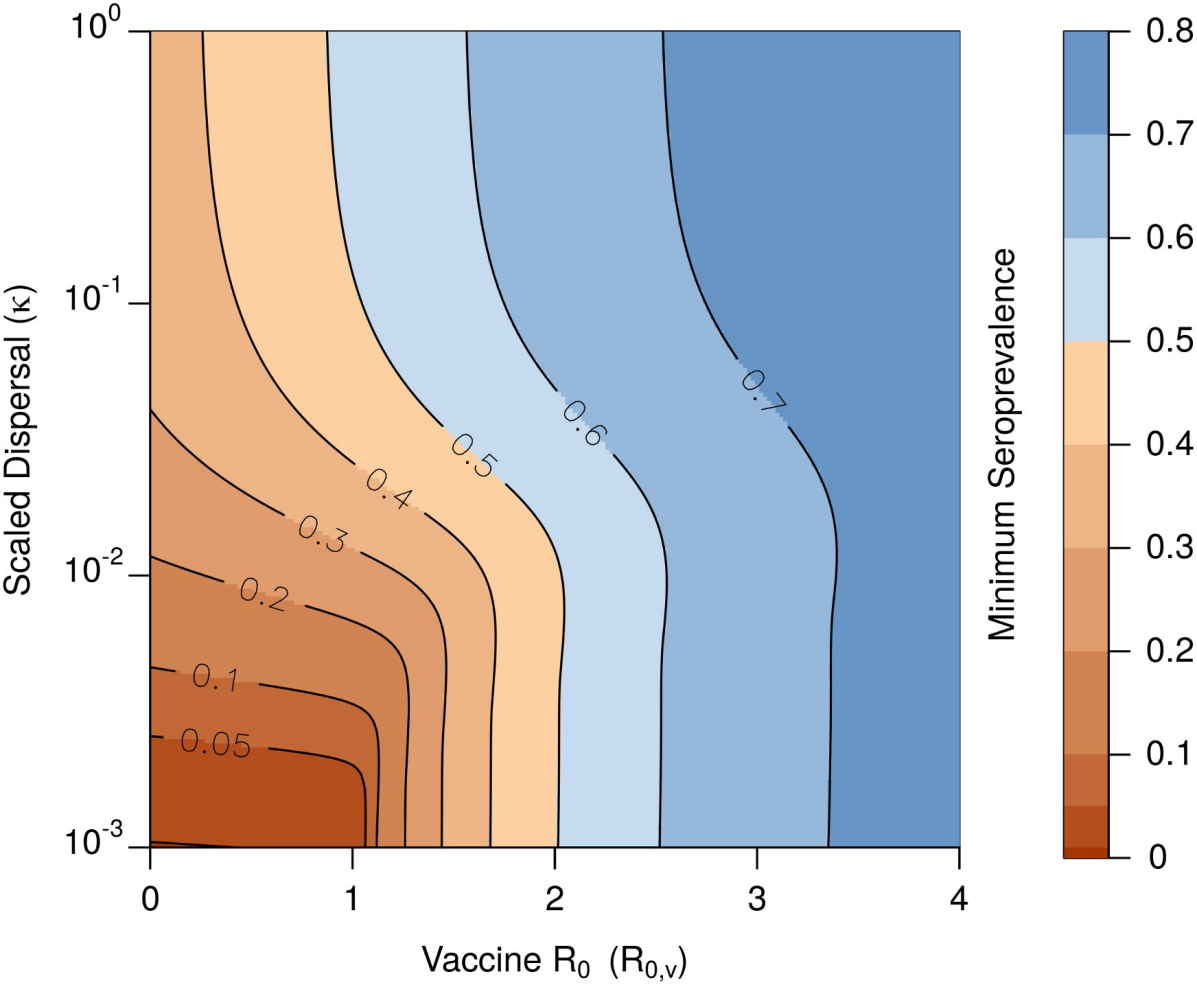
Minimal seroprevalence achieved with heterogeneous distribution of vaccine. With a flight-line spacing of 0.5 km, 0.013 ≤ *κ* ≤ 0.13 corresponds to host home ranges between 1 km^2^ and 10 km^2^. Vaccine distribution is tightly clustered (*ξ* = 0.025 km). Blue indicates seroprevalence values that exceed the 0.5 threshold required for herd immunity, red indicates values that do not. Other parameters: *d* = 0.416 yr^−1^, *σ* = 0.24 yr^−1^, *δ*_*v*_ = 12 yr^−1^.

A nondimsionalization of our model reveals that the parameter combination

$$\kappa =\frac{k}{\left(d+\delta_{v}\right)L^{2}}$$

determines the extent to which spatial heterogeneities in seroprevalence persist at steady state. Small values of *κ* describe scenarios where flight-line spacing is too large, relative to host dispersal, to significantly smooth out heterogeneities in seroprevalence. One way to overcome these heterogeneities is to increase vaccine transmission via *R*_0,*v*_. However, augmenting the spatial lows in seroprevalence requires relatively high levels of vaccine transmission (*R*_0,*v*_ > 1). Specifically, when scaled dispersal is small, *κ* ≈ 10^−2^, and the steady state distribution of baits is relatively clustered around each flight-line, increasing vaccine transmission from no transmission, *R*_0,*v*_ = 0, to modest transmission, *R*_0,*v*_ = 1, fails to substantially augment the minimal seroprevalence in the spatially explicit model (Fig 3). This demonstrates that weak transmission has a limited effect on augmenting seroprevalence lows that result from a heterogeneous bait distribution.

**Fig 4:**
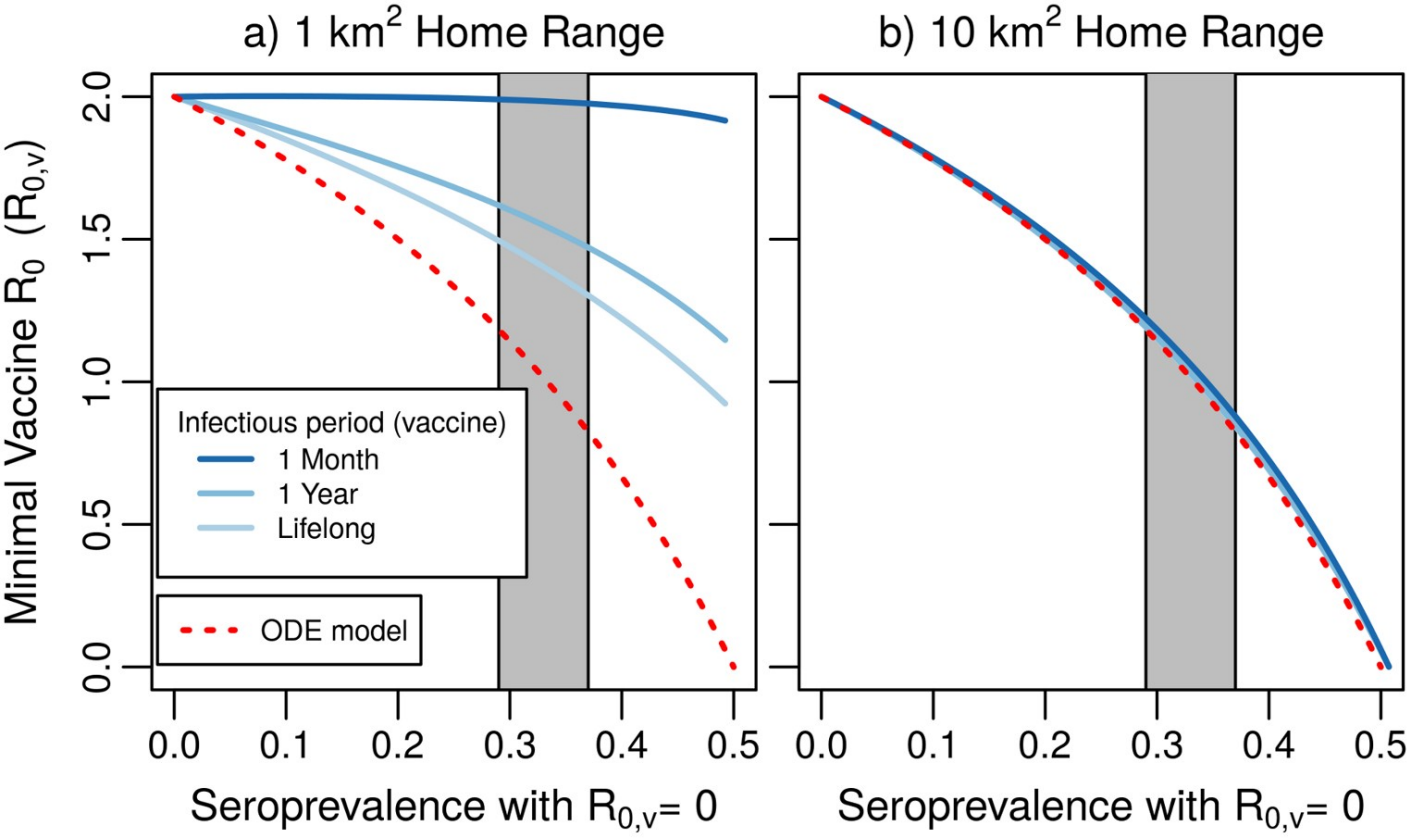
Vaccine transmission required for herd immunity in a nonhomogeneous population. Lines indicate the minimal vaccine transmission that augments spatially averaged seroprevalence to the 0.5 herd immunity threshold. We assume a tightly clustered bait distribution, *ξ* = 0.025 km. Gray region depicts typical seroprevalence levels achieved in US raccoon campaigns. In both panels, the minimal vaccine *R*_0,*v*_ required to protect a homogeneous population is plotted for comparison (“ODE model”). Other parameters: *d* = 0.416 yr^−1^.

The expression for *κ* implies that vaccines with longer infectious periods might be beneficial for overcoming spatial heterogeneities in vaccine coverage. For fixed *R*_0,*v*_, increasing the duration of vaccine infection increases the scaled dispersal parameter *κ*, which, in turn, smooths out spatial heterogeneities in the seroprevalence profile. In a host with a 1 km^2^ home range, our results indicate that establishing herd immunity requires *R*_0,*v*_ ≈ 2 when vaccine infection lasts 1 month, but only *R*_0,*v*_ ≈ 1.5 if the duration of infection is lifelong (Fig 4). However, our results also indicate that weak levels of vaccine transmission, even when paired with a longer duration of infection, will likely be ineffective at augmenting seroprevalence levels in raccoons with small home ranges. In contrast, for populations with larger home ranges, the required levels of vaccine transmission are similar to those predicted by the spatially homogeneous model, regardless of the duration of infection (Fig 4).

### Cost reduction in a homogeneous population

Our model provides broad estimates of the cost-savings that might be possible in campaigns that use transmissible vaccines. Assuming a homogeneous host population, the fractional reduction in the rate at which vaccine baits need to be distributed, while maintaining herd immunity at level *ϕ*, is

$$f_{\sigma }=R_{0,v}\;\left(1-\phi \right).$$

**Fig 5:**
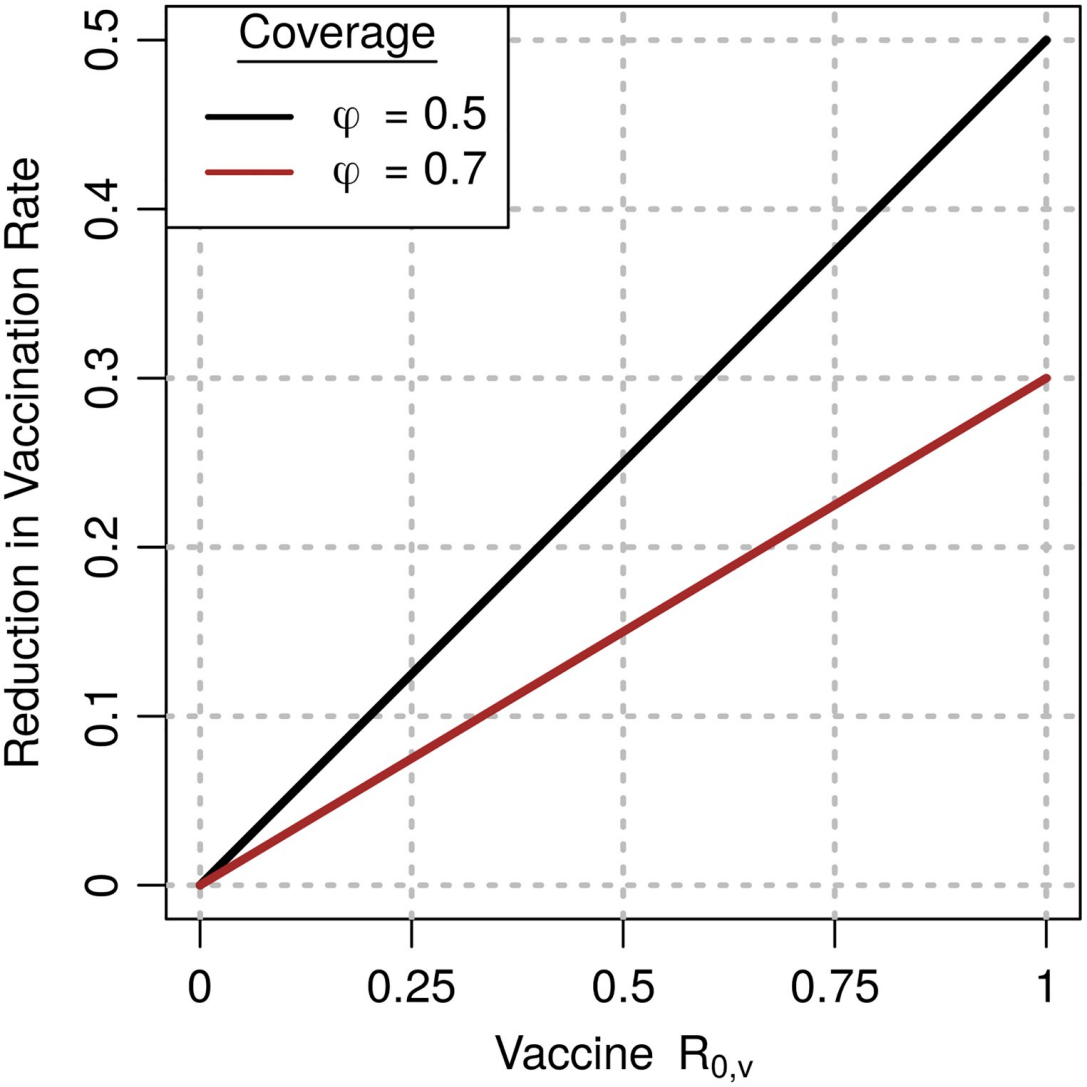
Fractional reduction in baits required for herd immunity in wildlife. The y-axis shows the reduction in the vaccine deposition rate due to vaccine transmission in campaigns that maintain herd immunity in North American wildlife (*ϕ* = 0.5), and at higher levels (*ϕ* ≈ 0.7) that are achieved in coyote populations.

The expression for *f*_*σ*_ implies that, in campaigns that seek to maintain herd immunity at a level *ϕ* = 0.5 in wildlife, a weakly transmitting vaccine with *R*_0,*v*_ = 0.5 would reduce the number of vaccine baits required each year by 25% (Fig 5). Evaluating *f*_*σ*_ with *R*_0,*v*_ = 1 shows that the maximal reduction in baits that is provided by weak transmission is 50%.

The cost-savings that are predicted by *f*_*σ*_ can be substantial. Between 2006 and 2010, vaccination efforts of the US Wildlife Services that targeted coyote and gray fox populations distributed approximately 2 million baits every year. In gray fox populations, these efforts resulted in an average seroprevalence of 0.69. *f*_*σ*_ implies that the corresponding reduction due to a vaccine with *R*_0,*v*_ = 0.9 is 27.9%. Given that 1.53 million baits were distributed each year, we calculate that by using a transmissible vaccine, the same seroprevalence could be achieved with 430,000 fewer baits per year. In coyotes, the average seroprevalence was 0.55, which implies that a 40.5% reduction in the number of baits is possible with *R*_0,*v*_ = 0.9. This reduction would bring the number of baits required each year down from 571,000 to 340,000. Using a cost per vaccine bait reported in other USDA campaigns, the total cost-savings on vaccine baits associated with transmission at *R*_0,*v*_ = 0.9 is $1.4 million each year (S1 Appendix [30]). Here, the savings due to vaccine transmission account for 32% of the estimated $4.4 million total bait costs.

### Cost reduction in nonhomogeneous populations

**Fig 6:**
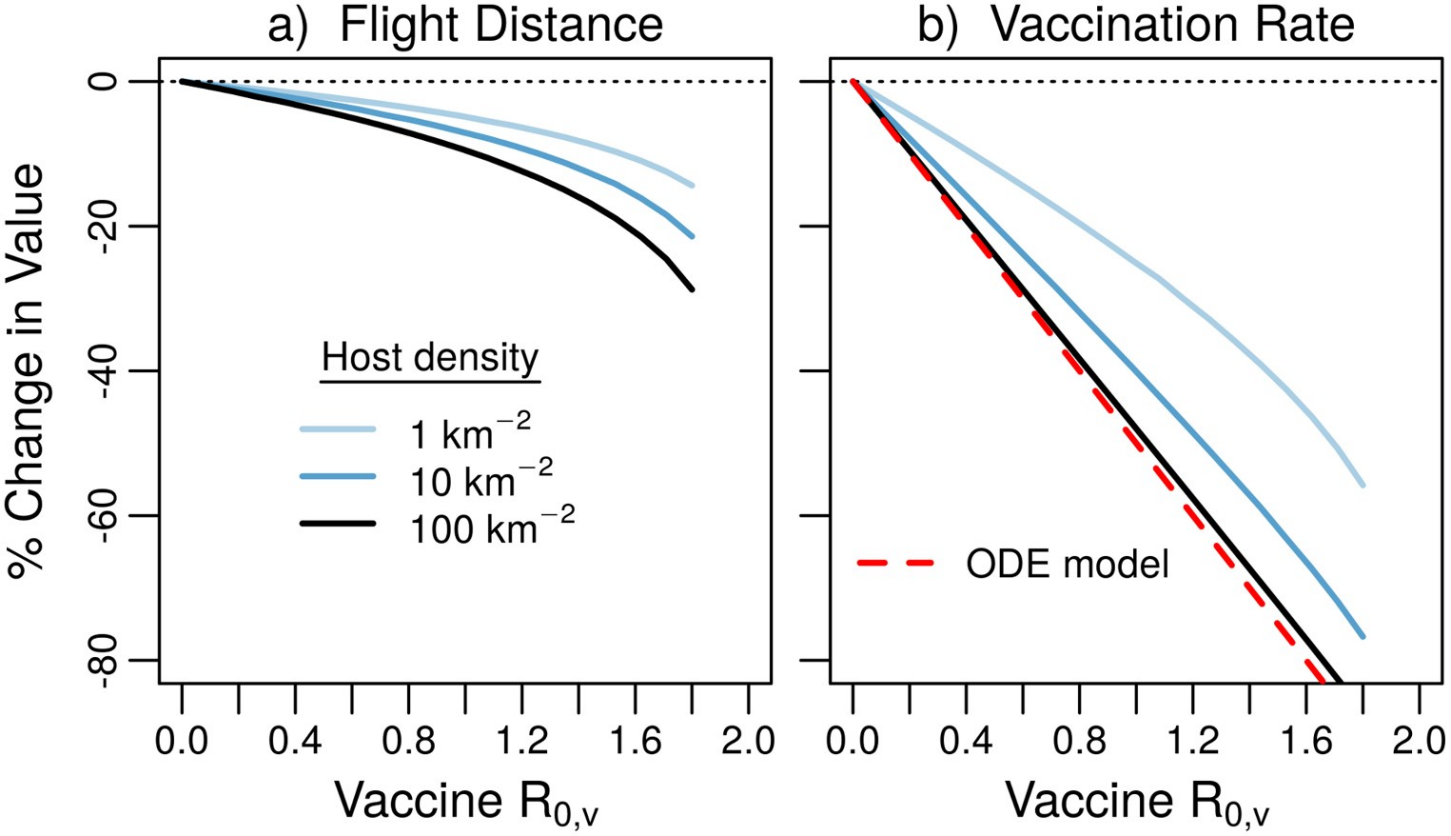
Proportional change in flight distance and vaccination rate due to vaccine transmission. The red dashed line shows the reduction in vaccine baits predicted for a homogeneous population. Other parameters, *d* = 0.416 yr^−1^, *δ*_*v*_ = 12 yr^−1^, *k* = 0.1 km^2^ yr^−1^, *ξ* = 0.25 km, *C*_*f*_ = 18.16 km^−1^, *C*_*b*_ = 2.12.

When the costs of aerial bait delivery are incorporated into our model, our results suggest that a transmissible vaccine can reduce the costs of vaccination programs in two ways: by reducing the spatial density of flight-lines that are necessary to ensure even coverage, and by reducing the rate at which vaccine baits need to be distributed (Fig 6). Parameterized with the aircraft and vaccine bait costs of Ohio campaigns between 1997 and 2000, our model finds the optimal flight-line spacing and vaccination rate that minimizes the costs of maintaining seroprevalence at 0.5. Though we do not prove it, numerical explorations suggest that the optimal combination of vaccination rate and flight-line spacing is unique (S1 Fig). Compared to the strategy using a vaccine that does not transmit, the effect of vaccine transmission on the optimal strategy is to reduce the vaccination rate, and to a lesser extent, decrease the total flight distance needed to distribute the vaccine by widening the flight-line spacing (Fig 6). Our results imply that the primary role of vaccine transmission is to reduce the quantity of vaccine that needs to be distributed along each flight-line, as opposed to changing how the vaccine baits are distributed spatially (Fig 6).

**Fig 7:**
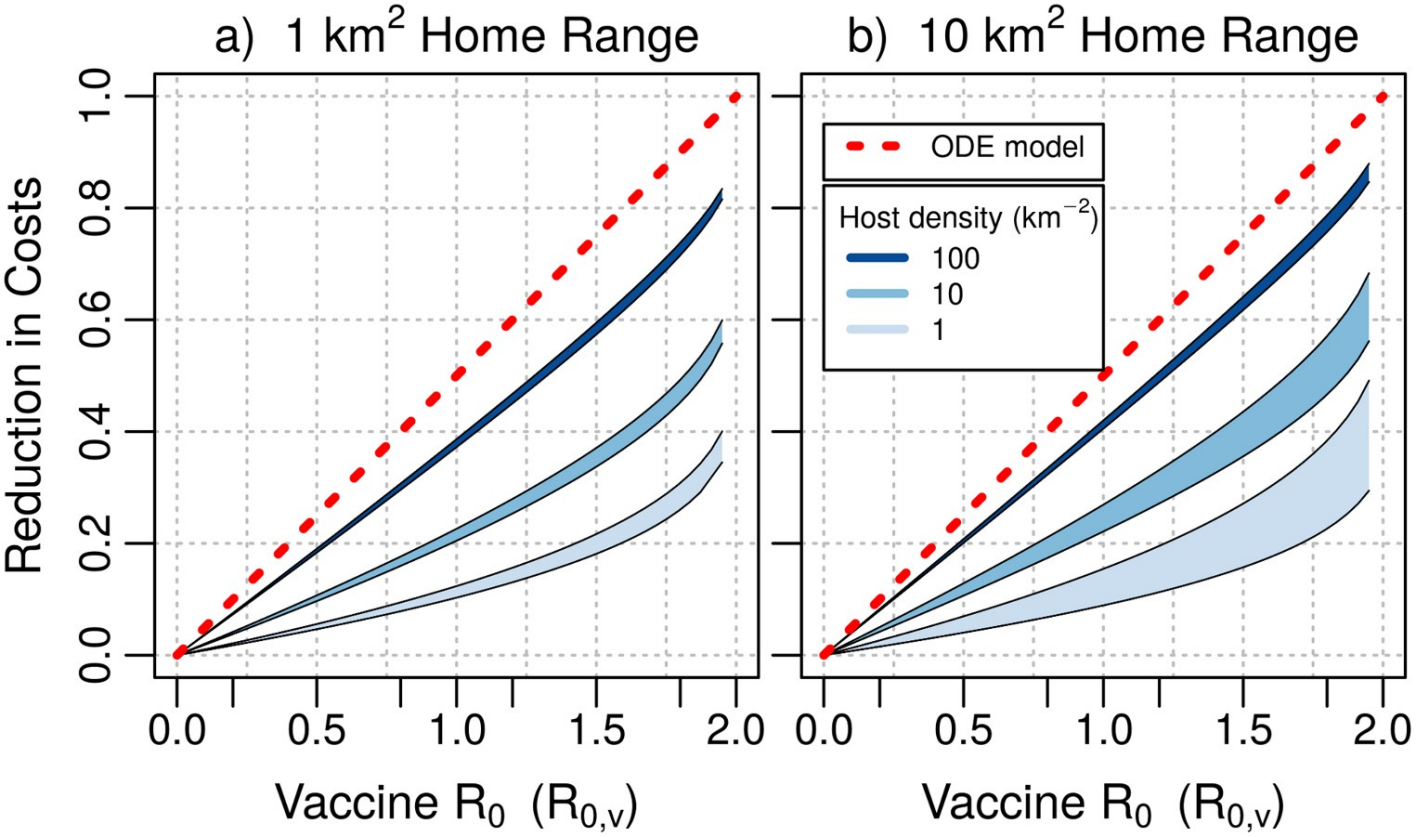
Fractional reduction in costs associated with the optimal vaccination strategy. Each region shows the cost-savings realized in a campaign that maintains seroprevalence at the 0.5 herd immunity threshold. Subfigures show model output when host home range is 1 km^2^ (panel a: *k* = 0.01) and 10 km^2^ (panel b: *k* = 0.1). Regions are bounded above and below by the reduction in costs that occur when the duration of vaccine infection is 1 year (*δ*_*v*_ = 1), and 1 month (*δ*_*v*_ = 12). The red line depicts the fractional reduction in vaccine baits predicted by the spatially homogeneous model. Other parameters: *d* = 0.416 yr^−1^, *ξ* = 0.25 km, *C*_*f*_ = 18.16 km^−1^, *C*_*b*_ = 2.12.

Our results imply that the cost-savings associated with vaccine transmission will be greatest in high density populations (Fig 7). The fractional reduction in costs predicted by the spatial model is always less than the savings predicted by the homogeneous model. This is due to the limited effect that vaccine transmission has on easing the flight-costs of vaccination. However, in campaigns that target host populations at high densities, the cost reductions that result are similar to those predicted by the homogeneous model, where the sole cost is the purchase of vaccine. In campaigns that target hosts at low densities, the flight costs comprise a greater proportion of the total costs. In this case, a vaccine with a long duration of infection can provide a greater reduction in total costs. For a modestly transmissible vaccine with *R*_0,*v*_ = 1, and moderate raccoon densities of 10 km^−2^, the net reduction in cost is about 20% when home range is small, and between 20-30% for larger home ranges, depending on the duration of infection.

### Sensitivity analysis of cost reduction

**Fig 8:**
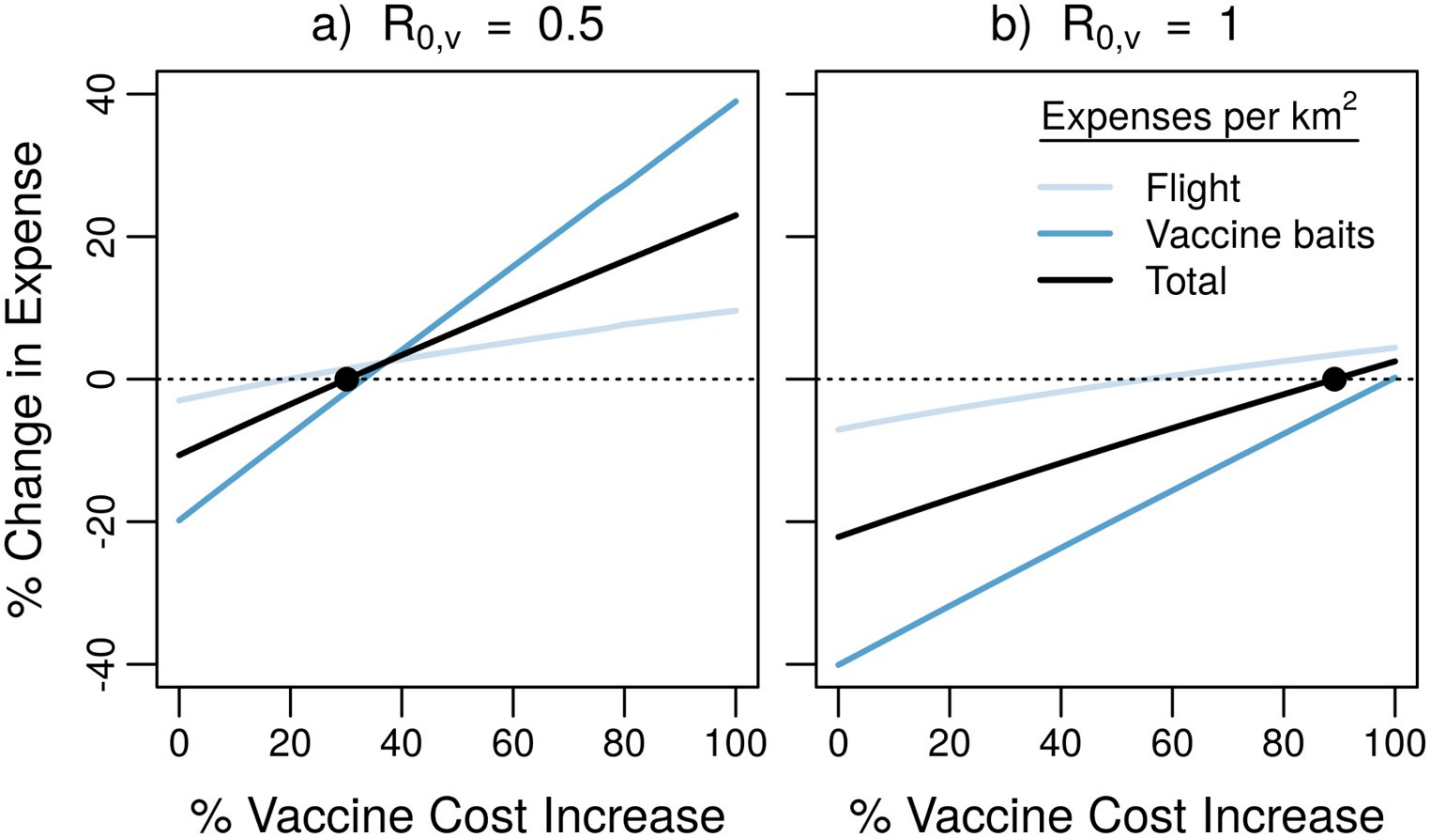
Cost reduction in typical ORV programs. We assume a baseline cost of *C*_*b*_ = 2.12 for a nontransmissible vaccine, and a higher per vaccine cost for a transmissible vaccine. Flight-Line costs are kept constant at *C*_*f*_ = 18.16 km^−1^. Other parameters set to *d* = 0.416 yr^−1^, *ξ* = 0.25 km.

Next, we investigate how our model’s prediction of the cost reduction due to vaccine transmission changes with different model assumptions. One important factor that will influence the anticipated cost savings is the price of vaccine baits that contain a transmissible vaccine virus, compared to the price of conventional, nontransmissible baits. Our model shows that, for a vaccine that transmits at a level *R*_0,*v*_ = 0.5, up to a 30% increase in vaccine bait cost can be tolerated and still reduce the overall costs of the campaign. If the vaccine transmits at *R*_0,*v*_ = 1, an increase of 89% is allowable (Fig 8).

Table 2 summarizes how modifying other assumptions of the baseline model changes the cost-reduction that is provided by vaccine transmission. When parameterized to a vaccine with *R*_0,*v*_ = 1 and a host with a 10 km^2^ home range, our baseline model predicts a 22% reduction of the summed aircraft and vaccine unit costs. Similar reductions occur when hosts that are vaccinated with a transmissible vaccine do not gain rabies immunity until after they recover from vaccine infection (“Lagged immunity” variant), or when the underlying distribution of vaccines is tightly clustered. Even greater reductions are predicted when limitations of the host cause rabies-immunity to wane after an average period of one year (“Temporary immunity” variant), or if vaccine transmission occurs throughout a host’s lifespan (“Lifelong vaccine infection” variant). However, this reduction is only 16% if high levels of seroprevalence are necessary for herd immunity, or if the transmissible vaccine is 25% more expensive than the nontransmissible vaccine.

**Table 2:** Cost reductions predicted by variations of the spatial model.

Model Variant	Total	Flight	Vaccine
Baseline model	22%	4%	18%
0.7 herd immunity threshold	16%	2%	15%
Temporary immunity	29%	4%	25%
Clustered vaccine distribution	22%	5%	16%
Lagged immunity	21%	4%	17%
Lifelong vaccine infection	29%	10%	19%
25% Increased vaccine cost	16%	2%	14%

“Total” column shows the percent reduction in total costs that result from using a transmissible vaccine with *R*_0,*v*_ = 1, relative a nontransmissible vaccine. “Flight” and “Vaccine” columns decompose the total reduction into savings from flight expenses, and expenses due to the purchase of vaccine baits. Percentages are rounded to the nearest whole number. The Baseline model describes a host population density of 10 km^−2^. Other parameters of the Baseline model are: *d* = 0.416 yr^−1^, *δ*_*v*_ = 12 yr^−1^, *ξ* = 0.25 km, *C*_*f*_ = 18.16 km^−1^, *C*_*b*_ = 2.12.

## Discussion

Oral vaccine technology has proven effective at vaccinating widely dispersed animal populations, and in turn protecting wildlife against zoonoses that harm humans [9, 10]. At the same time, however, ongoing campaigns against rabies in North America have identified challenges that must be overcome for wildlife vaccination to become a broadly applicable tool for controlling human pathogens in wildlife reservoir populations. Specifically, the difficulties associated with delivering the vaccine to widely distributed wildlife populations, and the costs of manufacturing sufficient quantities of vaccine, constrain the extent to which herd immunity can be established, and the length of time herd immunity can be maintained [13, 32]. When paired with oral vaccination technology, our mathematical results indicate that a vaccine with the capacity for weak transmission could help to overcome these challenges, facilitating robust and cost-effective control of zoonotic pathogens.

Historically, ORV campaigns have struggled to establish herd immunity in raccoon populations, due to low rates of seroconversion in raccoons that consume baits, as well as competition from non-targeted species that consume vaccine baits [13–15]. When parameterized by the range in seroprevalences achieved in raccoons by USDA campaigns, our results show that weak levels of vaccine transmission (*R*_0,*v*_ < 1) are capable of increasing seroprevalence levels to those required for herd immunity.

Moreover, for vaccination efforts that maintain herd immunity in North American wildlife, the costs associated with the purchase of vaccine baits can be decreased by up to 50% by a weakly transmissible vaccine. Because this estimate does not incorporate the costs of aerial delivery, these reductions are upper bounds on the total cost-savings that are possible with weak transmission. Even so, these estimates are relevant because the purchase of vaccine baits typically constitutes the majority of the total costs of oral vaccination programs [33]. When the costs of distributing baits with aircraft are incorporated, the cost-reductions predicted by our model are more modest, but still substantial. Our results indicate that weak transmission can cut between 20-30% of the total costs associated with protecting a reservoir population like raccoons against rabies.

In addition to the number of secondary infections, described by the vaccine *R*_0,*v*_, the duration of vaccine infection can influence the overall effectiveness of weak vaccine transmission. Our model predicts that for fixed vaccine *R*_0,*v*_, vaccines with longer periods of infection result in a greater reduction in the overall costs of a vaccination campaign. In hosts with large home ranges and low population density, a vaccine with a long infectious period can cut an additional 7% of the costs of vaccination, relative to the savings that result when a transmissible vaccine with a short infectious period is used. Currently, herpesviruses such as cytomegalovirus are being considered as vaccine vectors due to their relatively low virulence, natural occurrence in mammalian populations, apparent ability to reinfect hosts, and host specificity [20]. Though the epidemiological details of cytomegaloviruses are still being uncovered, in human, simian, and mouse models these viruses are broadly characterized by weak levels of infection that sometimes persist for life [34, 35]. As such, transmissible vaccines that are vectored by cytomegalovirus may allow greater reductions in costs, especially in hosts typified by large home ranges, and in populations with high host densities.

Our model also clarifies the role of vaccine transmission in augmenting oral vaccination campaigns. The self-disseminating properties of transmissible vaccines will amplify the effect that a fixed number of vaccine baits has on a population’s seroprevalence, and reduce the rate at which vaccine baits need to be deposited in order to maintain herd immunity. Our results also indicate that weak transmission is relatively ineffective at augmenting spatial minimums in seroprevalence that might arise because of flight-line style vaccination. In light of this, weak transmission may hold the most promise in campaigns that achieve an even distribution of vaccine baits, but are unable to establish herd immunity due to the challenges associated with limited host access.

Although insightful, our model simplifies several potentially important aspects of the vaccination process. Most obviously, our results are based upon model predictions at steady state. In contrast, population seroprevalence will fluctuate in time due to seasonal birth pulses that introduce new susceptibles into the host population, pulse-style vaccination campaigns, and waning immunity. In addition, accurately predicting the time-dependent outcome for a transmissible vaccine will require more flexible models that are able to incorporate short-term host movements, details of the vaccine’s design, as well as details of how hosts interact. Our model assumes that transmission is frequency-dependent, meaning that the number of infectious interactions does not scale with population size. Because our results stem from model behavior at steady state, similar outcomes would be expected for density-dependent transmission [36]. In contrast, how transmission scales with population size will be important in non steady state models, especially for wildlife populations that experience annual fluctuations due to seasonal reproduction [37].

The need for more detailed models will become greater as future empirical studies clarify the potential for engineering transmissible vaccines. Agent-based simulations, for example, could be used to incorporate contact structure of raccoon populations in different environments, stochasticity in the infection process, and predict at a fine scale the spread of different raccoon vectors. This undertaking will also require accurate details of the vaccination program, including details of how vaccines are distributed in the environment. Our spatially explicit results assume a fixed location of flight-lines in the environment. Consequently, the flight-line vaccination model presented here describes a worst-case scenario where the campaign relies entirely on host movement to avoid heterogeneities in vaccine coverage. This assumption might be appropriate, however, for campaigns that distribute baits from vehicles along roadways.

Our results focus on preemptive vaccination scenarios in which the pathogen has not yet spread within the host population. More generally, transmissible vaccines will need to function in populations that have pre-existing immunity from the pathogen. This might be an important limitation, for example, in targeting bat reservoirs of Ebola and rabies [20]. In terrestrial carnivores, however, rabies is typically not an immunizing infection. As a result, competition between the pathogen and the vaccine is minimal in the scenarios we model.

We have focused on quantifying the benefits of vaccine transmission for rabies circulating within North American wildlife because long-running oral vaccination campaigns provide opportunities for estimating key model parameters. However, our results suggest that transmissible vaccines could be used in other wildlife reservoirs for which parenteral vaccination is not possible. These results motivate the continued investigation of transmissible vaccines that target zoonotic diseases.

### Conclusion

Technology that engineers vaccine transmission may never be deemed safe for use in humans, but empirical studies have shown its efficacy and safety in non-human animals [20–22]. The need for better vaccine technology, particularly in the control of zoonotic pathogens in free-ranging animal populations, is apparent from ongoing campaigns in the US. A primary concern for the anticipated use of transmissible vaccines is the extent to which vaccine transmission can be engineered. Our results, combined with the capacity of oral vaccine campaigns to distribute vaccine to free-ranging host populations, demonstrate that weak vaccine transmission should be explored as a means of augmenting campaigns that do not achieve seroprevalence levels that are required for herd immunity. Namely, our results imply that weak vaccine transmission could bolster ongoing rabies campaigns that target raccoons, yet fail to establish herd immunity. More generally, though empirical research into the engineering of transmissible vaccines is still in its early stages, our results indicate that even weak levels of vaccine transmission could play a major role in the global control of infectious disease.

## Supporting information

S1 Appendix Model parameterization. (PDF)

S2 Appendix Details of model analysis. Provides a derivation of the vaccination rate parameter *σ* from a model that assumes a constant deposition rate of vaccine baits. This appendix also provides details of the nondimensionalization of the spatially explicit model, as well as details of the model variants that were explored.
(PDF)

S3 Appendix Model analysis in mathematica. This zip file contains a Mathematica file, as well as a PDF of the Mathematica file’s contents. The Mathematica file contains derivations for all analyses mentioned in the main text, including the calculation of steady state solutions, stability analysis, and the derivation of threshold conditions.
(ZIP)

S4 Appendix Numerical simulation. This zip file contains R scripts that simulate and plot the numerical results presented in the manuscript.
(ZIP)

S1 Fig Optimal combination of vaccination rate and flight-line spacing. Panel (a) shows the minimal vaccination rate, σ*, that is necessary to hold seroprevalence at *ϕ* = 0.5 for each flight-line spacing. Panel (b) shows the resulting costs of the strategies in panel (a). Dots indicate the cost-minimizing strategy. Other parameters: *d* = 0.416 yr^−1^, *δ*_*v*_ = 12 yr^−1^, *k* = 0.1 km^2^ yr^−1^, *ξ* = 0.25 km, *C*_*f*_ = 18.16 km^−1^, *C*_*b*_ = 2.12.
(TIF)
