# Supplementary material for: A little goes a long way: Weak vaccine transmission facilitates oral vaccination campaigns against zoonotic pathogens
Source: PLoS Negl Trop Dis. 2019 Mar 8;13(3):e0007251. doi: 10.1371/journal.pntd.0007251 (PMC6426267; doi:10.1371/journal.pntd.0007251)
Supplement: S1 Appendix — (PDF) [file pntd.0007251.s001.pdf]

S1 Appendix

We broadly parameterize our model to raccoon vaccination campaigns that occur in the US. To this end, we acquire parameters that describe raccoon population dynamics and home ranges. A non-dimensionalization of our spatially explicit model shows that the resulting seroprevalence profile depends only on the vaccine's basic reproduction number, $R_{0,v}$, the non-dimensional diffusion coefficient κ, and the scaled rates of mortality and vaccination, $\hat{d}$ and $\hat{\sigma}$. For simplicity, we only estimate those dimensional parameters that are necessary to find seroprevalence profiles in the non-dimensional version of the model. main text.

Raccoon demography (d, b):

Adult raccoons live, on average, for 964 days, which yields a natural mortality rate of $d = 0.416\,\mathrm{yr}^{-1}$ [1]. A non-dimensionalization of the spatially homogeneous and the spatially explicit model shows that the birth parameter b does not modify the steady state equilibrium values, nor the stability of the steady states when $R_{0,v}$ is held constant. This is commented on in the Mathematica file in S3. The birth parameter does influence the cost function when the vaccination rate σ is converted into a rate of bait deposition (See below).

Vaccination rate σ in USDA campaigns

Here, we derive a range of vaccination rates σ that reproduce historical seroprevalences achieved by USDA raccoon campaigns. When a nontransmissible vaccine is used to vaccinate a host population, the densities of rabies seronegative (S) and seropositive (V) hosts are described by

$$\begin{aligned} \frac{dS}{dt} &= b - \sigma S - dS \\ \frac{dV}{dt} &= \sigma S - dV. \end{aligned} \tag{1}$$

System (1) predicts that in long-term campaigns, the seroprevalence approaches a steady state

$$v^* = \frac{\sigma}{d + \sigma} \tag{2}$$

(S3 Appendix). We use Eq (2) to parameterize long-term vaccination effort in our model, described by σ, with the 5-year range of seroprevalence achieved in raccoons for each year between 2006 - 2010. During this time the USDA Rabies Management Program conducted seroprevalence surveys 4 - 12 weeks following vaccination of raccoons, and reported yearly seroprevalence averages that varied from a low of 0.29 in 2006, to a high of 0.37 in 2010. Interpreted as steady state seroprevalence levels, these values are converted into a range of vaccination rates by solving Eq (2) for σ:

$$\sigma = \frac{dv^*}{1 - v^*} \tag{3}$$

Fig 1 graphs the relationship between σ, and the observed steady state seroprevalence v^* given by Eq (3). The range of empirically measured seroprevalence imply that the effective vaccination rate lies between $\sigma = 0.17$ and $\sigma = 0.24$ (Fig 1).

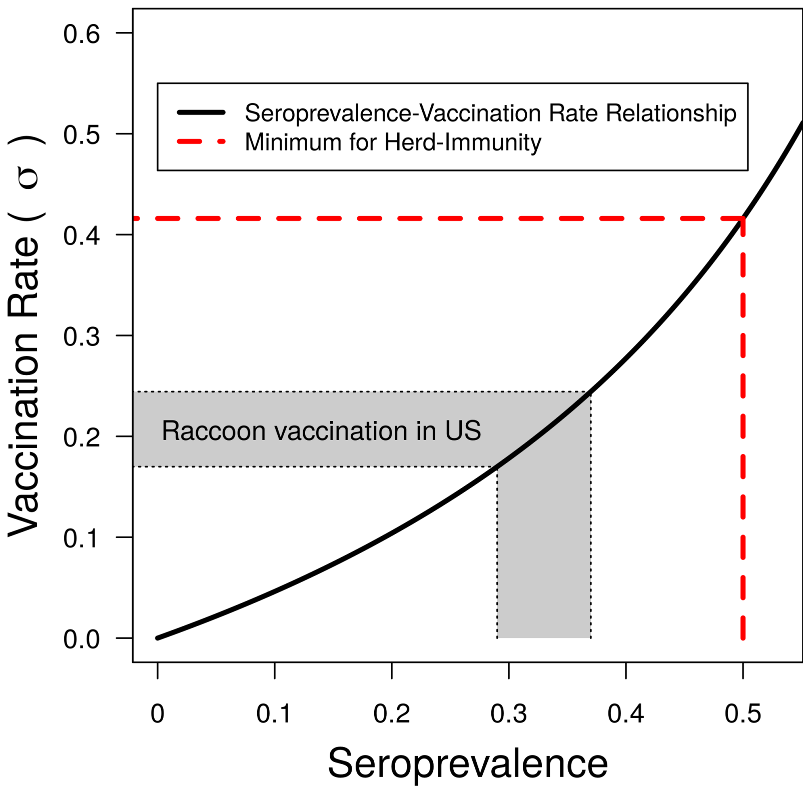

Figure 1. Parameterization of the per-capita vaccination rate in USDA campaigns. The dark gray region indicates the range of yearly averages for raccoon seroprevalence in the US, following vaccine bait drops between 2006 - 2010.

Diffusion coefficient (k):

The diffusion coefficient k describes the average dispersal of an adult raccoon throughout its lifetime. Below, we use empirical data on raccoon home ranges to estimate an appropriate range of k.

Empirical studies have found that raccoon home ranges are highly variable, depending largely on the availability of food resources [2]. In urban and peri-urban settings, raccoons seek out stable food sources at dumpsters and dump sites. These resources allow raccoons in urban or peri-urban areas to have smaller home ranges. We choose ranges with areas of 1 and 10 km^2 as a characteristic spread of ecologically plausible home ranges for raccoons in peri-urban and rural environments [1–3]. Next, we translate these home ranges into estimates for the 2D diffusion coefficient of a raccoon, k.

We use a simple diffusion sub-model of animal movement that describes the steady state spatial distribution of a raccoon that is released near a focal point $(x, y) = (0, 0)$. Upon release, the raccoon wanders its two-dimensional environment according to a random walk until death, at which point another raccoon is instantly released at position $(0, 0)$. At each time-step $\triangle t$, the lone, living raccoon takes steps of length $\pm\ell$ in either the x or y direction. Assuming that the probabilities of moving left, right, down, and up in a two-dimensional environment are equal and sum to unity, the probability distribution of the raccoon's location (x, y) at time t, s(x,y,t), is described by

$$\frac{\partial s}{\partial t} = k\left(\frac{\partial^2 s}{\partial x^2} + \frac{\partial^2 s}{\partial y^2}\right) + d\,I(x, y) - ds. \tag{4}$$

The domain is chosen to be the entire x-y plane. The diffusion coefficient k results from a diffusion limit of the random walk process,

$$k = \lim_{\substack{\triangle t \to 0 \\ \ell \to 0}} \frac{\ell^2}{4\,\triangle t}$$

[4]. Like other animal movement models, this description requires the step-length ℓ and time-step $\triangle t$ to balance so that k is finite. $I(x, y)$ is a probability distribution that describes where in space new raccoons are released.

Because Eq (4) assumes no directional biases exist in raccoon movement, the steady

state solutions will be radially symmetric so long as the distribution $I(x, y)$ is chosen to be radially symmetric. We assume I has radial symmetry, and as a consequence, can be rewritten in terms of the radial distance from the origin:
$I(x, y) = I_{pol}(\sqrt{x^2 + y^2}) = I_{pol}(r)$. With this assumption, Eq (4) is more conveniently expressed in polar coordinates, with the substitution of the new spatial variables $r = x^2 + y^2$ and $\theta = \text{atan2}(y, x)$. In its steady state form and described in polar coordinates, Eq (4) becomes

$$0 = k\frac{1}{r}\frac{\partial}{\partial r}\left(r\frac{\partial s}{\partial r}\right) - d(I_{pol}(r) - s). \tag{5}$$

When evaluated at steady state, Eq (5) describes the long-term probability distribution of raccoon locations. We use R's "stodes" function in the "rootSolve" package to solve for the spatial density of raccoon probability mass, predicted by Eq (5) for an organism with diffusion coefficient k and mortality d [5]. Similar to before, we use a finite difference scheme to convert Eq (5) into a finite set of algebraic equations. These equations are solved with an iterative root-solving method. In the numerical simulations, the solution $s(r)$ is solved at a finite set of discrete radial locations, $\{r_1, r_2, ..., r_{N+1}\}$, that describe the distribution of raccoon probability mass along concentric, annular rings of width $\triangle r$. Note that to allow numerical simulation, the spatial coordinate domain $0 < r < \infty$ is necessarily approximated by a set of points contained within a finite interval $0 < r < R$.

$I_{pol}(r)$ is chosen so that raccoons are released uniformly along the annulus that, in the discretized domain, is closest to the origin. This annulus is defined by two concentric circles with average radius r_1, and is the smallest annular ring on which the solution, s(r), is approximated.

$$I_{pol}(r) = \begin{cases} \frac{1}{2\pi r \triangle r} & \text{if } r == r_1 \\ 0 & \text{otherwise} \end{cases} \tag{6}$$

Chosen in this way, I_{pol} approximates a delta-Dirac distribution in polar coordinates, with probability mass one concentrated at the origin.

The steady state solution of Eq (5), $s^*(r)$, is a probability distribution that describes

the time-averaged distance between the raccoon's position while alive, and its birthplace, located at the origin, $r = 0$. We define a foraging area, $A_f = \pi r_f^2$, as the circular area that is bounded by the radial distance from the origin, r_f, that contains 0.9 of the probability mass of $s^*(r)$. In the continuum description of our model, r_f satisfies

$$2\pi \int_0^{r_f} s^*(r) r \, \mathrm{d}r = 0.9. \tag{7}$$

Here the factor $2\pi r \, \mathrm{d}r$ describes the differential area of the thin annuli over which integration occurs.

We use simulations across a range of k values to interpolate the diffusion coefficients that correspond to foraging areas of 1 and 10 km^2. Foraging areas of size 1 km^2 and 10 km^2 correspond to $k = 0.0128$ and $k = 0.1282$, respectively (Fig. 2). We use $k = 0.01$ and $k = 0.1$ in our simulations.

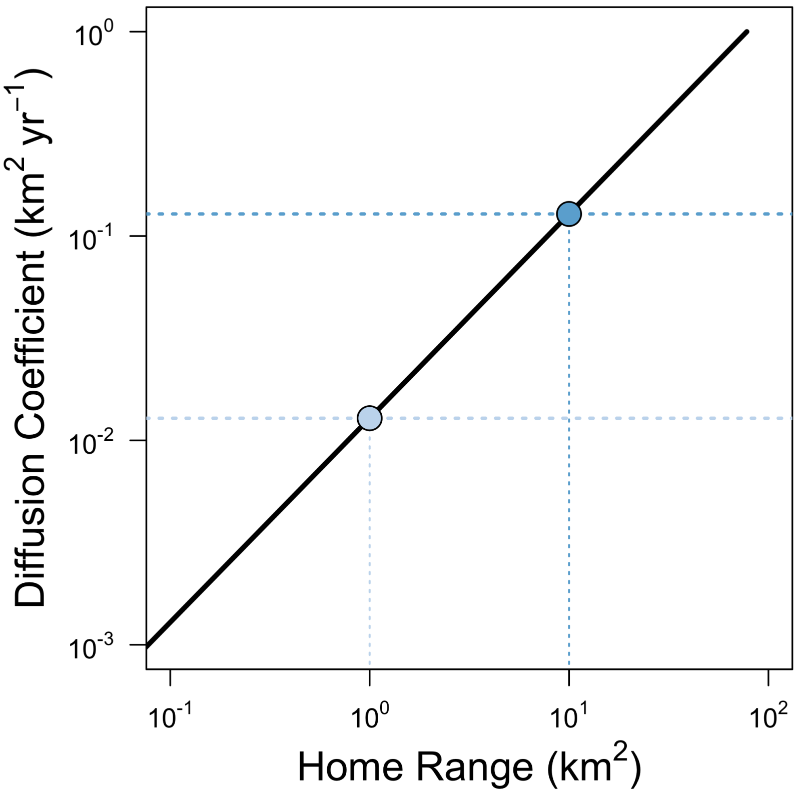

Figure 2. Graph of the diffusion coefficient as a function of home range. Raccoons with home ranges of 1 and 10 km^2 approximately correspond to diffusion coefficients of approximately $k = 0.01$ and $k = 0.1$. Raccoon mortality is set to $d = 0.416$.

Heterogeneity in vaccine baits (ξ):

We could not find empirical data that allowed us to parameterize the level of heterogeneity in flight-line campaigns. Instead, we choose values so that, when parameterized with a typical flight-line spacing of 0.5 km, the nondimensional parameter $\hat{\xi}$ takes on values 0.1 and 1. These values correspond to $\xi = 0.025$ km in the tightly clustered bait scenarios, and $\xi = 0.25$ km in the moderately clustered bait scenarios.

Costs of vaccine bait campaign (C_f, C_b, $\frac{b}{d}$):

In the spatially explicit model, the costs of the vaccine bait campaign are encapsulated in three parameters: C_f describes the cost per linear kilometer of flight, C_b describes cost per vaccine bait, and the steady state host density $\frac{b}{d}$ affects how many baits are required to increase the effective vaccination rate σ by a fixed amount. We use data from [6] to estimate the costs associated with distributing vaccine baits via aircraft, per linear kilometer of flight-line. The study calculates that, between 1997 - 2003, the flight costs of the Ohio campaign targeting raccoon rabies, including wages, maintenance, and fuel, amounted to \$24.71 per km^2. This allows us to calculate C_f as

$$C_f = (\text{Cost per km}^{-2}) \times (2L) \tag{8}$$

Choosing the typical flight-line spacing as 0.5 km ($L = 0.25$), and correcting for inflation of 47% between 2000 and 2018, this estimate gives a value of $C_f = 18.16$ [7]. The vaccine bait costs of Ohio campaigns averaged \$1.44 each, which gives an inflation-corrected value of $C_b = \$2.12$.

Completing the parameterization of the cost model requires specifying a relationship between the vaccination rate σ that is achieved given a bait deposition rate ρ. In S2 Appendix, we show that if vaccine bait competition is negligible, these parameters are related by host density,

$$\rho = \frac{b}{d}\sigma. \tag{9}$$

We choose values of $\frac{b}{d}$ that describe host densities of raccoons: 1, 10, and 100 km^{-2} [8,9].

References

1. Rosatte R, Ryckman M, Ing K, Proceviat S, Allan M, Bruce L, et al. Density, movements, and survival of raccoons in Ontario, Canada: implications for disease spread and management. J Mammal. 2010;91(1):122–135.

2. Totton SC, Rosatte RC, Tinline RR, Bigler LL. Seasonal home ranges of raccoons, Procyon lotor, using a common feeding site in rural eastern Ontario: rabies management implications. Can Field Nat. 2004;118(1):65–71.

3. Berentsen AR, Dunbar MR, Fitzpatrick CE, Walter WD. Spatial ecology of urban raccoons in northeastern Ohio: Implications for oral rabies vaccination. Prairie Nat. 2013;45:39–45.

4. Berg HC. Random walks in biology. Princeton University Press; 1993.

5. Soetaert K. rootSolve: Nonlinear root finding, equilibrium and steady-state analysis of ordinary differential equations; 2009.

6. Foroutan P, Meltzer MI, Smith KA. Cost of distributing oral raccoon-variant rabies vaccine in Ohio: 1997–2000. J Am Vet Med Assoc. 2002;220(1):27–32.

7. CPI Inflation Calculator. https://www.bls.gov/data/inflation_calculator.htm.

8. Nottingham BG, Johnson KG, Pelton MR. Evaluation of scent-station surveys to monitor raccoon density. Wildlife Society Bulletin (1973-2006). 1989;17(1):29–35.

9. Prange S, Gehrt SD, Wiggers EP. Demographic factors contributing to high raccoon densities in urban landscapes. J Wildl Manage. 2003; p. 324–333.
