# Supplementary material for: A little goes a long way: Weak vaccine transmission facilitates oral vaccination campaigns against zoonotic pathogens
Source: PLoS Negl Trop Dis. 2019 Mar 8;13(3):e0007251. doi: 10.1371/journal.pntd.0007251 (PMC6426267; doi:10.1371/journal.pntd.0007251)
Supplement: S2 Appendix — Provides a derivation of the vaccination rate parameter σ from a model that assumes a constant deposition rate of vaccine baits. This appendix also provides details of the nondimensionalization of the spatially explicit model, as well as details of the model variants that were explored. (PDF) [file pntd.0007251.s002.pdf]

S2 Appendix

Relationship between vaccination rate and vaccine baits

We first derive the relationship between the vaccination rate of susceptible hosts, σ, and the rate at which vaccine baits are deposited into the environment, ρ. For simplicity we use a a version of our model that does not include vaccine transmission, however a similar analysis would apply to the model with vaccine transmission. The altered form tracks the densities of seronegative and seropositive host classes, $S(t)$ and $V(t)$, and in addition tracks the density of vaccine baits $B(t)$ in the environment. Vaccine baits are distributed at rate ρ, and are depleted by the targeted host population at rate $c\,B(t)\,(S(t)+V(t))$. Here, c is the rate at which members of the host population find and consume vaccine baits. Besides the targeted host population, vaccine baits can also be depleted by non-targeted hosts or rendered ineffective by the environment [1, 2]. These effects are incorporated with a parameter that describes the rate of bait depletion, δ_B. Susceptible hosts that consume a vaccine bait acquire perfect, lifelong immunity to the rabies virus, while consumption of vaccine by a host that is already antibody seropositive has no effect on that host's vaccination status. With these assumptions, the time-varying spatial densities of vaccine baits (B), and antibody seronegative (S) and seropositive (V) individuals obeys

$$\begin{aligned} \frac{dB}{dt} &= \rho - \delta_B B - c(S+V)B \\ \frac{dS}{dt} &= b - cSB - dS \\ \frac{dV}{dt} &= cSB - dV. \end{aligned} \tag{1}$$

Because we are interested in seroprevalence outcomes in long-term vaccination campaigns, we solve for the steady state spatial densities of the state variables, notated B^*, S^* and V^*. By definition, steady states are solutions of system (1) that are constant in time, so the equations for the steady states are obtained by setting the time derivatives in system (1) to zero and solving the resulting system for the state-variables. We show in the supplementary mathematica file (S3 Model Analysis) that system (1)

has a unique, locally stable equilibrium:

$$\begin{aligned} B^* &= \frac{\rho}{c\frac{b}{d} + \delta_B} \\ S^* &= \frac{b}{d}\frac{bc + d\delta_B}{c(b+\rho) + d\delta_B} \\ V^* &= \frac{b}{d}\frac{c\rho}{c(b+\rho) + d\delta_B}. \end{aligned} \tag{2}$$

While mechanistically insightful, utilizing Eq's (2) requires estimates of vaccine bait parameters that are not easily measured. In order to simplify the parameter space, we introduce the per-capita, steady state vaccination rate parameter

$$\sigma = cB^* = \frac{c\rho}{c\frac{b}{d} + \delta_B}. \tag{3}$$

σ summarizes the steady state effect of vaccine baits into a single parameter, but still allows a mechanistic conversion from vaccination rate to rates of bait deposition and depletion. Because σ is proportional to ρ, a fractional reduction in the vaccination rate is equivalent to a fractional reduction in the rate at which baits need to be deposited. Note that if bait competition is negligible, $\delta_B = 0$, and Eq (3) simplifies to $\sigma = \rho(\frac{b}{d})^{-1}$.

As we are only interested in long-term vaccination programs, system (1) can be more clearly written as a simpler system where the contribution from vaccine baits is at equilibrium,

$$\begin{aligned} \frac{dS}{dt} &= b - \sigma S - dS \\ \frac{dV}{dt} &= \sigma S - dV. \end{aligned} \tag{4}$$

Derivation of spatial model

Here, we provide more details for the derivation of the spatial model presented in the main text. Vaccine baits are distributed along flight-lines. In the model, flight-lines are parallel lines in the x-y plane that extend infinitely far in the direction of the y-axis and are spaced a distance $2L$ km apart along the the x-axis. Because we assume that flight-lines extend infinitely far along the y-axis direction, the spatial densities of host classes only need to be tracked along the direction of the x-axis. To incorporate spatial heterogeneities in the distribution of vaccine baits due to flight-line vaccination, the spatially averaged deposition rate of baits, ρ yr^{-1} km^{-2}, is now distributed around each

flight-line according to the spatially dependent function $f(x)$, where x describes distance in the direction perpendicular to each flight-line. For the flight-line positioned at $x = 0$, $f(x)$ is a normal distribution on the interval $[-L, L]$, normalized so that $\int_{-L}^{L} f(x)\ \mathrm{dx} = 1$.

$$\begin{aligned} F(x) &:= \frac{1}{\sqrt{2\pi\xi^2}} \mathrm{Exp}\left(\frac{-x^2}{2\,\xi^2}\right) \\ f(x) &= \frac{F(x)}{\int_{-L}^{L} F(x)\,\mathrm{d}x} \end{aligned} \tag{5}$$

The extent to which vaccine infection spreads the vaccine spatially depends on the breadth of the hosts' home range. The home range size is incorporated by a diffusion coefficient k. Larger values of k describe animals with larger home ranges (details of the parameterization of k are in the S1 Appendix. With these assumptions, a transmissible vaccine-bait model of a single flight-line positioned at $x = 0$ is

$$\begin{aligned} \frac{\partial B}{\partial t} &= 2L\rho f(x) - \delta_B B - c(S + I_v + V)B \\ \frac{\partial S}{\partial t} &= k\frac{\partial^2 S}{\partial x^2} + b - cSB - dS - \frac{\beta_v S I_v}{S + I_v + V} \\ \frac{\partial I_v}{\partial t} &= k\frac{\partial^2 I_v}{\partial x^2} + cSB - (d + \delta_v)I_v + \frac{\beta_v S I_v}{S + I_v + V} \\ \frac{\partial V}{\partial t} &= k\frac{\partial^2 V}{\partial x^2} + \delta_v I_v - dV \end{aligned} \tag{6}$$

Note that in system (6) the vaccination rate ρ is multiplied by the period of the repeating flight-lines, $2L$. This ensures that the spatially averaged rate of bait deposition across each flight-line, described mathematically as

$$\frac{1}{2L}\int_{-L}^{L} 2L\rho f(x)dx = \rho,$$

is independent of the flight-line spacing that is chosen.

In the US, efforts of oral rabies vaccine campaigns often encompass hundreds of thousands of square kilometers of land [1, 3–5]. We focus on the interior of a large region so as to preclude effects from locations outside the zone of flight-line vaccination. As an approximation of flight-lines that repeat regularly in the environment across large distances, we choose Neumann boundary conditions that enforce spatial symmetry of the state variables across locations $x = -L$, and $x = L$. Choosing the boundary conditions

in this way incorporates the effects of an infinite number of adjacent flight-lines, centered at x-coordinates $\{... - 4L, -2L, 0, 2L, 4L...\}$. The symmetry of the spatial distribution of vaccine baits across $x = 0$ can be used to further simplify the model description. Specifically, we only track solutions on the truncated domain $x \in [0, L]$, which, as long as $f(x)$ is symmetric across $x = 0$, is a mirror image of the solution for $x \in [-L, 0]$. On the domain $0 \leq x \leq L$, the appropriate boundary conditions that impose symmetry at $x = 0$ and $x = L$ are, for each state variable $X \in \{S, I_v, V\}$,

$$\left.\frac{\partial X}{\partial x}\right|_{x=0} = 0 \qquad\qquad \left.\frac{\partial X}{\partial x}\right|_{x=L} = 0. \tag{7}$$

Table 1. Description of state variables in the model.

Name	Description
B	Vaccine baits
S	Susceptible to vaccine
I_v	Vaccine-infected
V	Recovered from vaccine

All state variables have units of $\frac{\text{Individuals}}{\text{km}^2}$. Hosts that become infected with the vaccine (I_v) are assumed to gain lifelong, perfect immunity. Infected hosts recover to class V, where they no longer transmit the vaccine, but maintain lifelong immunity.

As before, we rewrite system (6) in terms of a summarized per-capita vaccination rate σ, which is now an explicit function of space. At steady state, the per-capita rate of vaccination of susceptible hosts is $cB^*(x)$, where $B^*(x)$ is the spatially dependent density of vaccine baits. $B^*(x)$ is obtained by setting the time derivative in the $B(t)$ equation of system (6) to 0, and solving for the spatially dependent state variable B as a function of the steady state variables $S^*(x), I_v^*(x)$, and $V^*(x)$:

$$B^*(x) = \frac{2\,L\,f(x)\,\rho}{c(S^*(x) + I_v^*(x) + V^*(x)) + \delta_B} \tag{8}$$

The expression for B^* can be simplified by noting that the long-term spatial density of hosts, given by $S^*(x) + I_v^*(x) + V^*(x)$, is uniformly equal to $\frac{b}{d}$. This is true at steady state because the total population density, which in our model is defined by

$N(t,x) = S(t,x) + I_v(t,x) + V(t,x)$, satisfies

$$\begin{aligned} \frac{\partial N}{\partial t} &= \frac{\partial S}{\partial t} + \frac{\partial I_v}{\partial t} + \frac{\partial V}{\partial t} \\ &= b - dN(t,x) + k\frac{\partial^2 N}{\partial x^2}. \end{aligned} \tag{9}$$

This PDE has a unique biologically relevant steady state solution, $N^*(x) = \frac{b}{d}$, which is stable to perturbations (S3 Model Analysis). Given that N(t,x) evolves to the steady state solution $\frac{b}{d}$, we can rewrite the per-capita vaccination rate of susceptibles as

$$\begin{aligned} c\,B^*(x) &= c\,\frac{2\,L\,f(x)\,\rho}{c(S^*(x) + I_v^*(x) + V^*(x)) + \delta_B} \\ &= 2\,L\,f(x)\,\frac{c\rho}{c\frac{b}{d} + \delta_B} \\ &= 2\,L\,f(x)\,\sigma \end{aligned} \tag{10}$$

Eq (10) expresses the spatially dependent, per-capita steady state vaccination rate as a function of σ, defined in Eq (3), and the spatial distribution of vaccine baits across flight-lines spaced at intervals of $2L$, with a bait distribution $f(x)$ around each flight-line. We also use the fact that $N^*(x) = \frac{b}{d}$ to simplify the infection terms from $\frac{\beta_v S I_v}{S+I_v+V}$ to $\frac{d}{b}\beta_v S I_v$.

To understand how spatial heterogeneities influence the estimated vaccine $R_{0,v}$ that is necessary to augment campaigns targeting rabies in raccoons, we use the simplified system

$$\begin{aligned} \frac{\partial S}{\partial t} &= k\frac{\partial^2 S}{\partial x^2} + b - 2Lf(x)\sigma S - dS - \frac{d}{b}\beta_v S I_v \\ \frac{\partial I_v}{\partial t} &= k\frac{\partial^2 I_v}{\partial x^2} + 2Lf(x)\sigma S - (d+\delta_v)I_v + \frac{d}{b}\beta_v S I_v \\ \frac{\partial V}{\partial t} &= k\frac{\partial^2 V}{\partial x^2} + \delta_v I_v - dV \end{aligned} \tag{11}$$

with Neumann boundary conditions at $x = 0$ and $x = L$.

Nondimensionalization of spatial model

To clarify the relationship between flight-line spacing ($2L$), host home range (k), and the clustering parameter of vaccine baits (ξ), we nondimensionalize system (11) by

introducing new state variables, s, i_v, and v, defined as:

$$\frac{b}{d}s := S \quad \frac{b}{d}i_v := I_v \quad \frac{b}{d}v := V. \tag{12}$$

The nondimensional state variables track the density of each host class, scaled by the steady state population density $\frac{b}{d}$. In addition, we rescale space and time as $\chi = \dfrac{x}{L}$ and $\tau\dfrac{1}{d+\delta_v} = t$, respectively. The transformed space variable describes distance as a scaled multiple of the flight-line spacing parameter L, while the transformed time variable is measured with respect to the time that a vaccine-infected individual spends transmitting the vaccine to others. In addition, we introduce the non-dimensional parameters

$$\begin{aligned} \hat{\xi} &= \frac{\xi}{L} & R_{0,v} &= \frac{\beta_v}{d+\delta_v} & \hat{d} &= \frac{d}{d+\delta_v} \\ \hat{\sigma} &= \frac{\sigma}{d+\delta_v} & \kappa &= \frac{k}{(d+\delta_v)L^2} \end{aligned} \tag{13}$$

The spatial effects of flight-line vaccination are encapsulated in the non-dimensional parameter combinations that define $\hat{\xi}$ and κ. The function that describes the distribution of baits around each flight-line, notated in its non-dimensional form as $\hat{f}(\chi)$, remains a truncated normal distribution, now with standard deviation $\hat{\xi}$ and mean $\chi = 0$, that is normalized to integrate to 0.5 on the interval $0 < \chi < 1$ (thus integrating to 1 on the domain $-1 < \chi < 1$).

The nondimensionalized system is

$$\begin{aligned} \frac{\partial s}{\partial \tau} &= \kappa\frac{\partial^2 s}{\partial \chi^2} + \hat{d}(1-s) - 2\hat{\sigma}\hat{f}(\chi)\, s - R_{0,v} s\, i_v \\ \frac{\partial i_v}{\partial \tau} &= \kappa\frac{\partial^2 s}{\partial \chi^2} + 2\hat{\sigma}\hat{f}(\chi)\, s + R_{0,v} s\, i_v - i_v \\ \frac{\partial v}{\partial \tau} &= \kappa\frac{\partial^2 v}{\partial \chi^2} + (1-\hat{d})\, i_v - \hat{d}\, v \end{aligned} \tag{14}$$

with boundary conditions

$$\left.\frac{\partial X}{\partial \chi}\right|_{\chi=0} = 0 \qquad\qquad \left.\frac{\partial X}{\partial \chi}\right|_{\chi=1} = 0 \tag{15}$$

for $X \in \{s, i_v, v\}$.

Numerical simulation of spatial model

We numerically solve for steady states of system (14) to predict how the spatially explicit, steady state seroprevalence levels vary in long-term campaigns, given a nonhomogeneous distribution of vaccine baits in the environment. To this end, we solve a discretized version of system (14) that is obtained using a centered, second-order finite difference approximation. In order to better capture the spatial detail in solutions where the distribution of baits is tightly clustered around each flight-line (e.g. small $\hat{\xi}$), we use a grid with variable meshwidth chosen to focus gridpoints near $\chi = 0$. The resulting algebraic system is solved via the Newton-Raphson method, implemented with the "stodes" function in the statistical language R's "rootSolve" package [6, 7]. In rare cases where the iterative solver did not converge, we use the "lsodar" function of the "deSolve" package to dynamically run system (14) to steady state, starting from an initial condition with only susceptible individuals [8]. Here, steady state is assumed to be reached when the sum of the absolute values of the differentials is less than 0.0001.

We find steady states of system (14) across a range of parameter values, and use the spatially explicit seroprevalence profile to calculate the minimal seroprevalence that results. In non-dimensional parameters, this host population statistic is written

$$\min_{0 \leq \chi \leq 1} i_v^*(\chi) + v^*(\chi). \tag{16}$$

This, in turn, is used to understand the ability of vaccine transmission to augment campaigns that do not achieve a targeted herd immunity threshold, as well as the cost-savings that are possible using a transmissible vaccine. All R scripts mentioned in this text are available as supplementary materials (S4 Numerical Simulation).

Model Variants

In order to more fully understand the potential for variability in our cost analyses, we numerically simulate several variants of System (6). The model presented up to this point is referred to as the Baseline model, and is the model from which the variations are defined.

Lagged Immunity

In the baseline model, rabies-immunity in hosts occurs simultaneously with vaccine infection. In the Lagged immunity variant, a host is not immune to rabies until they have recovered from infection with the vaccine. This modification is incorporated by specifying that, for herd immunity, the density of vaccine-recovered hosts must be elevated to 0.5. Specifically, Eq (16) in the R script is modified to

$$\min_{0 \leq \chi \leq 1} v^{*}(\chi). \tag{17}$$

Temporary Immunity

When comparing the relative costs of using a nontransmissible and transmissible vaccine, we have assumed that rabies immunity is lifelong. We modify this assumption by incorporating a rate, α, at which immunized hosts transition back into the susceptible class and lose their rabies immunity. In our comparisons, we assume that waning immunity is a property of the host, and therefore applies to both the nontransmissible and transmissible vaccine scenarios. In our baseline model of a transmissible vaccine, rabies-immunity is assumed to occur as soon as a host is infected with the vaccine. Because of this, we assume that both vaccine-infected hosts and vaccine-recovered hosts lose immunity at rate α. The model variation is

$$\begin{aligned}
\frac{\partial S}{\partial t} &= k\frac{\partial^2 S}{\partial x^2} + b - 2Lf(x)\sigma S - dS - \frac{\beta_v S I_v}{S + I_v + V} \\
\frac{\partial I_v}{\partial t} &= k\frac{\partial^2 I_v}{\partial x^2} + 2Lf(x)\sigma S - (d + \delta_v)I_v + \frac{\beta_v S I_v}{S + I_v + V} - \alpha I_v \\
\frac{\partial V}{\partial t} &= k\frac{\partial^2 V}{\partial x^2} + \delta_v I_v - dV - \alpha V
\end{aligned} \tag{18}$$

with Neumann boundary conditions at $x = 0$ and $x = L$.

Other Variants

The remaining variations we explore use the baseline model with modified parameter values. These variants are: 1) an increased seroprevalence required for herd immunity, incorporated by setting $\phi = 0.7$ in Eq (16); 2) A vaccine distribution that is tightly clustered around flight-lines ($\xi = 0.025$); 3) Lifelong infection, incorporated by setting $\delta_v = 0$; and 4) a 25% increase in the cost of baits, included by increasing the cost of

vaccine baits from $C_b = 2.12$ to $C_b = 2.65$.

References

1. Sidwa TJ, Wilson PJ, Moore GM, Oertli EH, Hicks BN, Rohde RE, et al. Evaluation of oral rabies vaccination programs for control of rabies epizootics in coyotes and gray foxes: 1995–2003. J Am Vet Med Assoc. 2005; 227(5):785–792.

2. Sattler AC, Krogwold RA, Wittum TE, Rupprecht CE, Algeo TP, Slate D, et al. Influence of oral rabies vaccine bait density on rabies seroprevalence in wild raccoons. Vaccine. 2009; 27(51):7187–7193.

3. United States Department of Agriculture (USDA) National Rabies Management. https://www.aphis.usda.gov/wildlife_damage/oral_rabies/downloads/NationalReport_2010.pdf.

4. United States Department of Agriculture (USDA) ORV Distribution Summary; 2015. https://www.aphis.usda.gov/wildlife_damage/oral_rabies/oral_rabies_info_by_state/us/FY15%20National.pdf.

5. United States Department of Agriculture (USDA) ORV Distribution Summary; 2017. https://www.aphis.usda.gov/wildlife_damage/oral_rabies/oral_rabies_info_by_state/us/2017_National.pdf.

6. R Core Team. R: A Language and Environment for Statistical Computing; 2018. Available from: https://www.R-project.org/.

7. Soetaert K. rootSolve: Nonlinear root finding, equilibrium and steady-state analysis of ordinary differential equations; 2009.

8. Soetaert K, Petzoldt T, Setzer RW. Solving Differential Equations in R: Package deSolve. J Stat Softw. 2010; 33(9):1–25. doi:10.18637/jss.v033.i09.
