# Supplementary material for: A little goes a long way: Weak vaccine transmission facilitates oral vaccination campaigns against zoonotic pathogens
Source: PLoS Negl Trop Dis. 2019 Mar 8;13(3):e0007251. doi: 10.1371/journal.pntd.0007251 (PMC6426267; doi:10.1371/journal.pntd.0007251)
Supplement: S3 Appendix — This zip file contains a Mathematica file, as well as a PDF of the Mathematica file’s contents. The Mathematica file contains derivations for all analyses mentioned in the main text, including the calculation of steady state solutions, stability analysis, and the derivation of threshold conditions. (ZIP) [file pntd.0007251.s003.zip › S3_ModelAnalysis/S3_ModelAnalysis.pdf]

In this file, we present calculations that are alluded to in the methods and results of the main text.

Oral vaccine bait model (ODE)

Here, we show that the model presented in the main text (System 1) can be derived from a model that explicitly tracks the density of vaccine baits. This model is used to relate analyses that involve the per capita vaccination rate σ to quantities of vaccine baits deposited per year.

Vaccine bait model

Define the system of differential equations that describes the use of a non-transmissible vaccine. Also, define some conditions on the parameters that will be used in the analysis. Note that δ is used in place of δ_B to refer to the rate of bait depletion due to non-targeted hosts.

In[1]:=
```
Conds = {ρ > 0 && δ > 0 && d > 0 && b > 0 && c > 0};
dBdt = ρ - δ B - c (S + V) B;
dSdt = -c B S + b - d S;
dVdt = c B S - d V;
```

Solve for steady-state seroprevalence

Solve for the steady states of the above system by setting the time derivatives of the state variables to 0. The seroprevalence of the population is $\frac{V^*}{\frac{b}{d}}$, the steady-state number of vaccinated individuals divided by the steady-state population density.

In[5]:=
```
Eqns = {dBdt == 0, dSdt == 0, dVdt == 0};
SolNoTrans = FullSimplify[Solve[Eqns, {B, S, V}]]
```

Out[6]=
$$\left\{\left\{B \to \frac{d\,\rho}{b\,c + d\,\delta},\ S \to \frac{b\,(b\,c + d\,\delta)}{d\,(d\,\delta + c\,(b + \rho))},\ V \to \frac{b\,c\,\rho}{d\,(d\,\delta + c\,(b + \rho))}\right\}\right\}$$

These are Equations (2) of the S2 Appendix. When parameterized with biologically relevant parameter values, the equilibrium state variables are positive and therefore biologically relevant. Next, check that the above steady-state is stable by ensuring that all eigenvalues have a negative real part. The Jacobian is computed as:

In[7]:=
```
JacMat = {D[dBdt, {{B, S, V}}], D[dSdt, {{B, S, V}}], D[dVdt, {{B, S, V}}]} ;
JacMat // MatrixForm
```

Out[8]//MatrixForm=
$$\begin{pmatrix} -c\,(S + V) - \delta & -B\,c & -B\,c \\ -c\,S & -B\,c - d & 0 \\ c\,S & B\,c & -d \end{pmatrix}$$

Evaluated at equilibrium, the eigenvalues of the system are

In[9]:=
```
FullSimplify[Eigenvalues[JacMat /. SolNoTrans], Conds]
```
Out[9]= $\left\{-d, -\frac{b\,c + d\,\delta}{d}, d\left(-1 - \frac{c\,\rho}{b\,c + d\,\delta}\right)\right\}$

These are all clearly negative, indicating that the vaccine bait model possesses a single, stable steady-state.

Introduce the parameter $\sigma = c\,B^*$

Simplify the parameter space of the above model by introducing the parameter σ, which describes the rate at which susceptible individuals are vaccinated at steady state: σ = c B^*.

In[11]:=
```
SigSolve = σ → c (B /. SolNoTrans[[1]])
```
Out[11]= $\sigma \to \frac{c\,d\,\rho}{b\,c + d\,\delta}$

This can be written as $\sigma = \frac{c\,\rho}{c\,\frac{b}{d} + \delta_b}$, which is Equation (3) of the S2 Appendix. With the vaccination rate specified through σ, the model can at steady state be simplified to

In[12]:=
```
Clear[σ]
dSdtSig = -σ S + b - d S;
dVdtSig = σ S - d V;
```

Next, find the equilibrium values of S and V in terms of the summary parameter σ

In[15]:=
```
EqnsSig = {dSdtSig == 0, dVdtSig == 0};
SolNoTransSig = FullSimplify[Solve[EqnsSig, {S, V}]]
```
Out[16]= $\left\{\left\{S \to \frac{b}{d + \sigma}, V \to \frac{b\,\sigma}{d\,(d + \sigma)}\right\}\right\}$

Thus, the fraction of individuals vaccinated, written in terms of the effective vaccination rate σ, is $v^* = V^* \big/ \left(\frac{b}{d}\right) = \frac{\sigma}{d + \sigma}$. This is Equation (3) of the S1 Appendix.

In[17]:=
```
SeroNoTransSig = σ/(σ + d);
```

Transmissible vaccine bait model (ODE, σ = 0)

Define the model

Here, we analyze the scenario without continual vaccination. The resulting model is System 1 of the main text with σ set to 0.

```
Conds = {Bv > 0 && b > 0 && d > 0 && δv > 0};

In[18]:= dSdtTrans = b - d S - Bv S Iv / (S + Iv + V);
dIvdtTrans = Bv S Iv / (S + Iv + V) - (d + δv) Iv;
dVdtTrans = δv Iv - d V;
```

Find steady-states of the model

Solve for the steady state number of vaccinated individuals, as before.

```
In[21]:= EqnsTransSig0 = {dSdtTrans == 0, dIvdtTrans == 0, dVdtTrans == 0};
SolTransSig0 = FullSimplify[Solve[EqnsTransSig0, {S, Iv, V}], Conds[[1]]]
```

Out[22]= $\left\{\left\{S \to \frac{b}{d}, Iv \to 0, V \to 0\right\}, \left\{S \to \frac{b\,(d+\delta_v)}{Bv\,d}, Iv \to b\left(-\frac{1}{Bv} + \frac{1}{d+\delta_v}\right), V \to \frac{b\,(Bv - d - \delta_v)\,\delta_v}{Bv\,d\,(d+\delta_v)}\right\}\right\}$

In order to rewrite these steady-states in terms of the vaccine R_0(termed Rv), we replace instances of β_v with Rv ($d + \delta_v$).

```
In[23]:= SolTransSig0 = FullSimplify[SolTransSig0 /. Bv → Rv (d + δv), Conds && Rv > 0]
```

Out[23]= $\left\{\left\{S \to \frac{b}{d}, Iv \to 0, V \to 0\right\}, \left\{S \to \frac{b}{d\,Rv}, Iv \to \frac{b\,(-1+Rv)}{Rv\,(d+\delta_v)}, V \to \frac{b\,(-1+Rv)\,\delta_v}{d\,Rv\,(d+\delta_v)}\right\}\right\}$

Biological relevance of equilibria

Equilibrium 1

This equilibrium describes the steady state in which the transmissible vaccine is absent.

Equilibrium 2

```
In[24]:= Conds = {Rv > 0 && σ > 0 && δv > 0 && d > 0 && b > 0}
Simplify[Reduce[(S /. SolTransSig0[[2]]) > 0 && Conds[[1]] ], Conds[[1]]]
```

Out[24]= $\{Rv > 0 \,\&\&\, \sigma > 0 \,\&\&\, \delta_v > 0 \,\&\&\, d > 0 \,\&\&\, b > 0\}$

Out[25]= True

```
In[26]:= Simplify[Reduce[(Iv /. SolTransSig0[[2]]) > 0 && Conds[[1]] ], Conds[[1]]]
```

Out[26]= $Rv > 1$

Equilibrium 2 describes the case where the vaccine is transmitting sufficiently to maintain an endemic presence in the host population. This equilibrium is biologically relevant as long as the basic reproduction number of the vaccine is greater than 1. Store that condition:

```
In[27]:= Eq2Exists = Rv > 1;
```

Stability of the equilibria

Stability of the transmissible vaccine system is not impacted by the recovered class, as V individuals due not contribute to new infections. Therefore we only investigate stability in the reduced system of susceptible and vaccine-infected individuals.

Equilibrium 1

In[28]:=
```
JacMat = {D[dSdtTrans /. Bv → Rv (d + δv), {{S, Iv}}],
    D[dIvdtTrans /. Bv → Rv (d + δv), {{S, Iv}}]};
JacMat // MatrixForm
```

Out[29]//MatrixForm=

$$\begin{pmatrix} -d + \frac{Iv\,Rv\,S\,(d+\delta_v)}{(Iv+S+V)^2} - \frac{Iv\,Rv\,(d+\delta_v)}{Iv+S+V} & \frac{Iv\,Rv\,S\,(d+\delta_v)}{(Iv+S+V)^2} - \frac{Rv\,S\,(d+\delta_v)}{Iv+S+V} \\ -\frac{Iv\,Rv\,S\,(d+\delta_v)}{(Iv+S+V)^2} + \frac{Iv\,Rv\,(d+\delta_v)}{Iv+S+V} & -d - \delta_v - \frac{Iv\,Rv\,S\,(d+\delta_v)}{(Iv+S+V)^2} + \frac{Rv\,S\,(d+\delta_v)}{Iv+S+V} \end{pmatrix}$$

Next, find the eigenvalues of the system.

In[30]:=
```
Conds = {Rv > 0 && σ > 0 && δv > 0 && d > 0 && b > 0}
```

Out[30]= $\{Rv > 0 \,\&\&\, \sigma > 0 \,\&\&\, \delta_v > 0 \,\&\&\, d > 0 \,\&\&\, b > 0\}$

In[31]:=
```
FullSimplify[Eigenvalues[JacMat /. SolTransSig0[[1]]], Conds[[1]] ]
```

Out[31]= $\{-d, (-1 + Rv)\ (d + \delta_v)\}$

The second eigenvalue becomes positive when Rv > 1; thus, the steady state with the vaccine absent becomes unstable when Rv > 1.

Equilibrium 2

Evaluated at equilibrium, the eigenvalues of the system are

In[32]:=
```
Evals = FullSimplify[Eigenvalues[JacMat /. SolTransSig0[[2]]] /. Bv → Rv (d + δv),
   Conds && Rv > 0 && d + δv > 0]
```

Out[32]=

$$\left\{\frac{1}{2}\left(-d\,Rv - \sqrt{\frac{d}{Rv}}\ \sqrt{d\,(-2 + Rv)^2\,Rv - 4\,(-1 + Rv)^2\,\delta_v}\right),\ \frac{1}{2}\left(-d\,Rv + \sqrt{\frac{d}{Rv}}\ \sqrt{d\,(-2 + Rv)^2\,Rv - 4\,(-1 + Rv)^2\,\delta_v}\right)\right\}$$

Check stability of the equilibrium by evaluating the sign of the eigenvalues. To ensure the equilibrium is biologically relevant, we require that the condition Eq2Exists is satisfied.

In[40]:=
```
FullSimplify[Reduce[Re[Evals[[1]]] < 0 && Conds[[1]] && Eq2Exists],
 Eq2Exists && Conds[[1]]]
```

Out[40]= True

In[34]:=
```
FullSimplify[Reduce[Re[Evals[[2]]] < 0 && Eq2Exists && Conds[[1]]],
  Eq2Exists && Conds[[1]] ]
```
Out[34]=
```
True
```

Both eigenvalues are negative whenever the 2nd equilibrium is biologically relevant. As such, the steady state with the vaccine present is stable when it exists (i.e. when Rv > 1). This condition coincides with that required for instability in the vaccine-absent equilibrium.

Biological implications / results

Solutions of the endemic infection system evolve to the vaccine-absent equilibrium, SolTransSig0[[1]], when Rv < 1, and to the equilibrium that describes endemic infection, SolTransSig0[[2]], when Rv > 1.

Autonomous vaccine seroprevalence

Equilibrium 2 predicts the seroprevalence that results when a population is exposed to a highly transmissible vaccine. When scaled by the steady state host density $\frac{b}{d}$, the sum of the vaccine-infected and vaccine-recovered state variables gives the equilibrium seroprevalence as

In[41]:=
```
FullSimplify[(Iv + V) / (b / d) /. SolTransSig0[[2]]]
```
Out[41]= $\frac{-1 + Rv}{Rv}$

Transmission necessary for herd-immunity ϕ

In order to vaccinate a fraction ϕ of the population, Rv must satisfy

In[42]:= Solve$\left[\frac{-1 + Rv}{Rv} == \phi, Rv\right]$

Out[42]= $\left\{\left\{Rv \to \frac{1}{1 - \phi}\right\}\right\}$

This is equation 12 of the main text.

Transmissible vaccine bait model (ODE, $\sigma > 0$)

Define the model

Modify the model presented above to include both vaccine transmission and steady vaccination. We work with the vaccination rate parameter σ, defined as before, to predict the extent to which vaccine transmission can augment seroprevalence achieved in US campaigns that target rabies

in raccoons. Below, define system (1) of the main text.

```
In[43]:= Conds = {Bv > 0 && σ > 0 && b > 0 && d > 0 && Rv > 0};
```

```
In[44]:= dSdtTrans = -σ S + b - d S - Bv S / (S + Iv + V) Iv;
dIvdtTrans = σ S + Bv S Iv / (S + Iv + V) - (d + δv) Iv;
dVdtTrans = δv Iv - d V;
```

Later on, we use simulation to show that the vaccinated equilibrium is stable. Simulation is easier with fewer parameters, so we non-dimensionalize the system. To this end, we introduce the following parameter replacements:

dh = $\frac{d}{d+\delta_v}$

Rv = $\frac{Bv}{d+\delta_v}$

σh = $\frac{\sigma}{d+\delta_v}$

It must be true that 0< dh < 1. All other non-dimensional parameters are non-negative. In addition, we rescale the state variables by the steady state population density, $\frac{b}{d}$ **(i.e. s = S/ (**$\frac{b}{d}$**), etc). Note that lower case letters are used to denote the scaled state variables. Substituting these new parameters into the previous system yields:**

```
In[47]:= dsdtTrans = -σh s + dh (1 - s) - Rv s / (s + iv + v) iv;
divdtTrans = σh s + Rv s iv / (s + iv + v) - iv;
dvdtTrans = (1 - dh) iv - dh v;
```

Find steady-states of the model with vaccination σh > 0

Solve for the steady state number of vaccinated individuals, as before.

```
In[50]:= EqnsTransSig = {dsdtTrans == 0, divdtTrans == 0, dvdtTrans == 0};
SolTransSig = FullSimplify[Solve[EqnsTransSig, {s, iv, v}], Conds[[1]]]
```

Out[51]=

$$\left\{\left\{s \to \frac{dh + dh\,Rv + \sigma h - \sqrt{-4\,dh^2\,Rv + (dh + dh\,Rv + \sigma h)^2}}{2\,dh\,Rv},\right.\right.$$
$$iv \to \frac{dh\,(-1 + Rv) - \sigma h + \sqrt{-4\,dh^2\,Rv + (dh + dh\,Rv + \sigma h)^2}}{2\,Rv},$$
$$\left. v \to -\frac{1}{2\,dh\,Rv}(-1 + dh)\left(dh\,(-1 + Rv) - \sigma h + \sqrt{-4\,dh^2\,Rv + (dh + dh\,Rv + \sigma h)^2}\right)\right\},$$
$$\left\{s \to \frac{dh + dh\,Rv + \sigma h + \sqrt{-4\,dh^2\,Rv + (dh + dh\,Rv + \sigma h)^2}}{2\,dh\,Rv},\right.$$
$$iv \to -\frac{dh - dh\,Rv + \sigma h + \sqrt{-4\,dh^2\,Rv + (dh + dh\,Rv + \sigma h)^2}}{2\,Rv},$$
$$\left.\left. v \to \frac{1}{2\,dh\,Rv}(-1 + dh)\left(dh - dh\,Rv + \sigma h + \sqrt{-4\,dh^2\,Rv + (dh + dh\,Rv + \sigma h)^2}\right)\right\}\right\}$$

Check which equilibria are biologically relevant

Equilibrium 1:

In[52]:=
```
Conds = {Rv > 0 && σh > 0 && dh > 0 }
Simplify[Reduce[(s /. SolTransSig[[1]]) > 0 && Conds[[1]] ], Conds[[1]]]
```

Out[52]= {Rv > 0 && σh > 0 && dh > 0}

Out[53]= True

In[54]:=
```
Simplify[Reduce[(iv /. SolTransSig[[1]]) > 0 && Conds[[1]] ], Conds[[1]]]
```

Out[54]= True

In[55]:=
```
Simplify[Reduce[(v /. SolTransSig[[1]]) > 0 && Conds[[1]] ], Conds[[1]]]
```

Out[55]= dh < 1

Equilibrium 1 is biologically relevant when the parameters are biologically relevant.

Equilibrium 2:

In[56]:=
```
Conds = {Rv > 0 && dh > 0 && σh > 0 }
Simplify[Reduce[(s /. SolTransSig[[2]]) > 0 && Conds[[1]] ], Conds[[1]]]
```

Out[56]= {Rv > 0 && dh > 0 && σh > 0}

Out[57]= True

In[58]:=
```
Simplify[Reduce[(iv /. SolTransSig[[2]]) > 0 && Conds[[1]] ], Conds[[1]]]
```

Out[58]= False

In[59]:=
```
Simplify[Reduce[(v /. SolTransSig[[2]]) > 0 && Conds[[1]] ], Conds[[1]]]
```

Out[59]= dh > 1

Equilibrium 2 is not biologically relevant when the parameters are biologically relevant. This equilibrium is discarded.

Evaluate the stability of the relevant equilibrium

In[60]:=
```
JacMat = {D[dsdtTrans, {{s, iv}}], D[divdtTrans, {{s, iv}}]} ;
JacMat // MatrixForm
```

Out[61]//MatrixForm=

$$\begin{pmatrix} -dh + \frac{iv\,Rv\,s}{(iv+s+v)^2} - \frac{iv\,Rv}{iv+s+v} - \sigma h & \frac{iv\,Rv\,s}{(iv+s+v)^2} - \frac{Rv\,s}{iv+s+v} \\ -\frac{iv\,Rv\,s}{(iv+s+v)^2} + \frac{iv\,Rv}{iv+s+v} + \sigma h & -1 - \frac{iv\,Rv\,s}{(iv+s+v)^2} + \frac{Rv\,s}{iv+s+v} \end{pmatrix}$$

Evaluated at equilibrium, the eigenvalues of the system are

In[62]:=
```
Conds = {Rv > 0 && σh > 0 && dh > 0}
```
Out[62]=
```
{Rv > 0 && σh > 0 && dh > 0}
```

In[63]:=
```
Evals = FullSimplify[Eigenvalues[JacMat /. SolTransSig[[1]]], Conds[[1]] ]
```

Out[63]=

$$
\begin{aligned}
\Bigg\{ & -\frac{1}{4\, dh\, Rv}\Bigg(Rv\left(-\sigma h+\sqrt{dh^2\,(-1+Rv)^2+2\,dh\,(1+Rv)\,\sigma h+\sigma h^2}\right)+ \\
& dh\left(1+dh-Rv+dh\,Rv+\sigma h+\sqrt{dh^2\,(-1+Rv)^2+2\,dh\,(1+Rv)\,\sigma h+\sigma h^2}\right)+ \\
& \sqrt{2}\,\sqrt{\Bigg(Rv\left(dh^4\,(4-3\,Rv+Rv^3)+Rv\,\sigma h\left(\sigma h-\sqrt{dh^2\,(-1+Rv)^2+2\,dh\,(1+Rv)\,\sigma h+\sigma h^2}\right)+\right.} \\
& \quad dh\Bigg(Rv\sqrt{dh^2\,(-1+Rv)^2+2\,dh\,(1+Rv)\,\sigma h+\sigma h^2}- \\
& \quad Rv^2\left(-2\,\sigma h+\sqrt{dh^2\,(-1+Rv)^2+2\,dh\,(1+Rv)\,\sigma h+\sigma h^2}\right)+ \\
& \quad 4\,\sigma h\left(-\sigma h+\sqrt{dh^2\,(-1+Rv)^2+2\,dh\,(1+Rv)\,\sigma h+\sigma h^2}\right)\Bigg)+ \\
& \quad dh^2\Bigg(-2\,Rv^2+Rv^3+4\Bigg(\sqrt{dh^2\,(-1+Rv)^2+2\,dh\,(1+Rv)\,\sigma h+\sigma h^2}+ \\
& \quad \sigma h\left(-2+\sigma h-\sqrt{\left(dh^2\,(-1+Rv)^2+2\,dh\,(1+Rv)\,\sigma h+\sigma h^2\right)}\right)\Bigg)+ \\
& \quad Rv\Bigg(1-6\sqrt{dh^2\,(-1+Rv)^2+2\,dh\,(1+Rv)\,\sigma h+\sigma h^2}+ \\
& \quad \sigma h\left(-2+\sigma h+\sqrt{\left(dh^2\,(-1+Rv)^2+2\,dh\,(1+Rv)\,\sigma h+\sigma h^2\right)}\right)\Bigg)\Bigg)+ \\
& \quad dh^3\Bigg(-4\left(1-2\,\sigma h+\sqrt{dh^2\,(-1+Rv)^2+2\,dh\,(1+Rv)\,\sigma h+\sigma h^2}\right)+ \\
& \quad Rv\Bigg(6+6\,\sigma h+\sqrt{dh^2\,(-1+Rv)^2+2\,dh\,(1+Rv)\,\sigma h+\sigma h^2}+ \\
& \quad Rv\left(-2+2\,\sigma h+\sqrt{\left(dh^2\,(-1+Rv)^2+2\,dh\,(1+Rv)\,\sigma h+\sigma h^2\right)}\right)\Bigg)\Bigg)\Bigg)\Bigg)\Bigg), \\
& \frac{1}{4\, dh\, Rv}\Bigg(-Rv\left(-\sigma h+\sqrt{dh^2\,(-1+Rv)^2+2\,dh\,(1+Rv)\,\sigma h+\sigma h^2}\right)+ \\
& dh\left(1+dh-Rv+dh\,Rv+\sigma h+\sqrt{dh^2\,(-1+Rv)^2+2\,dh\,(1+Rv)\,\sigma h+\sigma h^2}\right)+ \\
& \sqrt{2}\,\sqrt{\Bigg(Rv\left(dh^4\,(4-3\,Rv+Rv^3)+Rv\,\sigma h\left(\sigma h-\sqrt{dh^2\,(-1+Rv)^2+2\,dh\,(1+Rv)\,\sigma h+\sigma h^2}\right)+\right.} \\
& \quad dh\Bigg(Rv\sqrt{dh^2\,(-1+Rv)^2+2\,dh\,(1+Rv)\,\sigma h+\sigma h^2}- \\
& \quad Rv^2\left(-2\,\sigma h+\sqrt{dh^2\,(-1+Rv)^2+2\,dh\,(1+Rv)\,\sigma h+\sigma h^2}\right)+ \\
& \quad 4\,\sigma h\left(-\sigma h+\sqrt{dh^2\,(-1+Rv)^2+2\,dh\,(1+Rv)\,\sigma h+\sigma h^2}\right)\Bigg)+ \\
& \quad dh^2\Bigg(-2\,Rv^2+Rv^3+4\Bigg(\sqrt{dh^2\,(-1+Rv)^2+2\,dh\,(1+Rv)\,\sigma h+\sigma h^2}+\sigma h \\
& \quad \left(-2+\sigma h-\sqrt{dh^2\,(-1+Rv)^2+2\,dh\,(1+Rv)\,\sigma h+\sigma h^2}\right)\Bigg)+ \\
& \quad Rv\Bigg(1-6\sqrt{dh^2\,(-1+Rv)^2+2\,dh\,(1+Rv)\,\sigma h+\sigma h^2}+\sigma h \\
& \quad \left(-2+\sigma h+\sqrt{dh^2\,(-1+Rv)^2+2\,dh\,(1+Rv)\,\sigma h+\sigma h^2}\right)\Bigg)\Bigg)+ \\
& \quad dh^3\Bigg(-4\left(1-2\,\sigma h+\sqrt{dh^2\,(-1+Rv)^2+2\,dh\,(1+Rv)\,\sigma h+\sigma h^2}\right)+ \\
& \quad Rv\Bigg(6+6\,\sigma h+\sqrt{dh^2\,(-1+Rv)^2+2\,dh\,(1+Rv)\,\sigma h+\sigma h^2}+Rv \\
& \quad \left(-2+2\,\sigma h+\sqrt{dh^2\,(-1+Rv)^2+2\,dh\,(1+Rv)\,\sigma h+\sigma h^2}\right)\Bigg)\Bigg)\Bigg)\Bigg)\Bigg)\Bigg\}
\end{aligned}
$$

We wish to show these eigenvalues have negative real part, thus implying that equilibrium 1 is stable. Analytical approaches to showing stability were not successful. Instead, we use numeri-

cal evaluation of the eigenvalue expressions, across the range of parameter combinations used in the manuscript, to show that the equilibrium is stable.

dh = $\frac{0.416}{0.416+\delta_v}$ <= 1

$\sigma h \leq \frac{2}{0.416 + \delta_v} \leq 5$

$\sigma h \leq \frac{2}{0.416 + \delta_v} \leq 5$

Rv <= 2

Plot the real part of Eigenvalue 1, across a range of non-dimensional parameter values.

```
ContourPlot[Re[Evals[[1]]] /. dh → 0.1, {σh, 0, 5}, {Rv, 0, 2}]
```

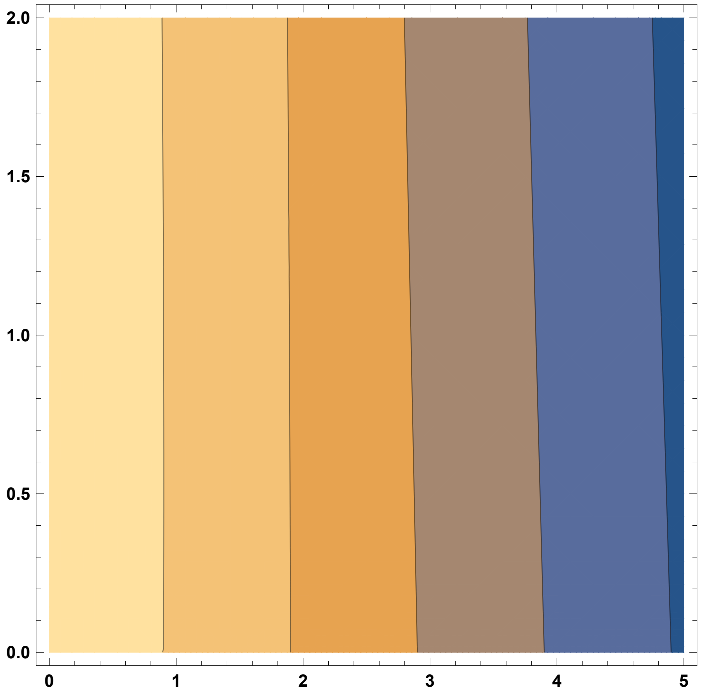

```
ContourPlot[Re[Evals[[1]]] /. dh → 0.5, {σh, 0, 5}, {Rv, 0, 2}]
```

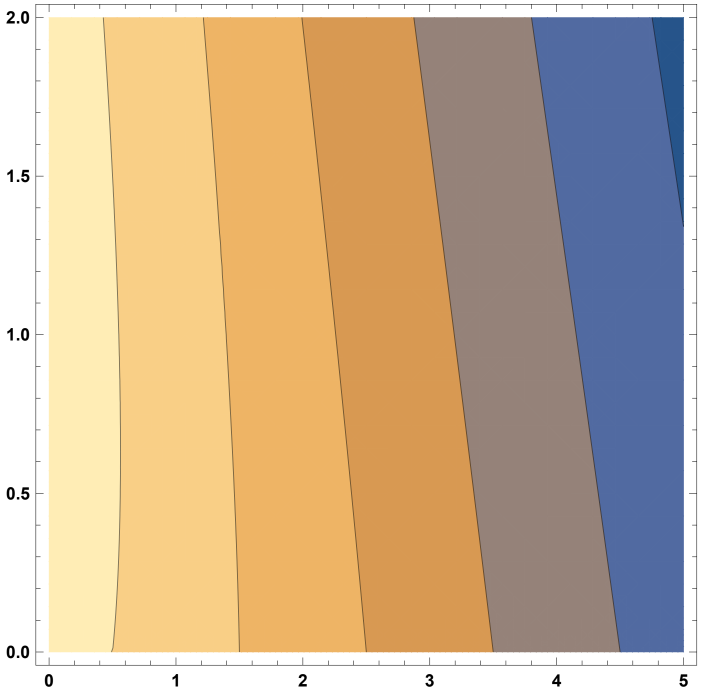

Check Eigenvalue 2:

```
ContourPlot[Re[Evals[[2]]] /. σh → 0.5, {dh, 0, 1}, {Rv, 0, 2}]
```

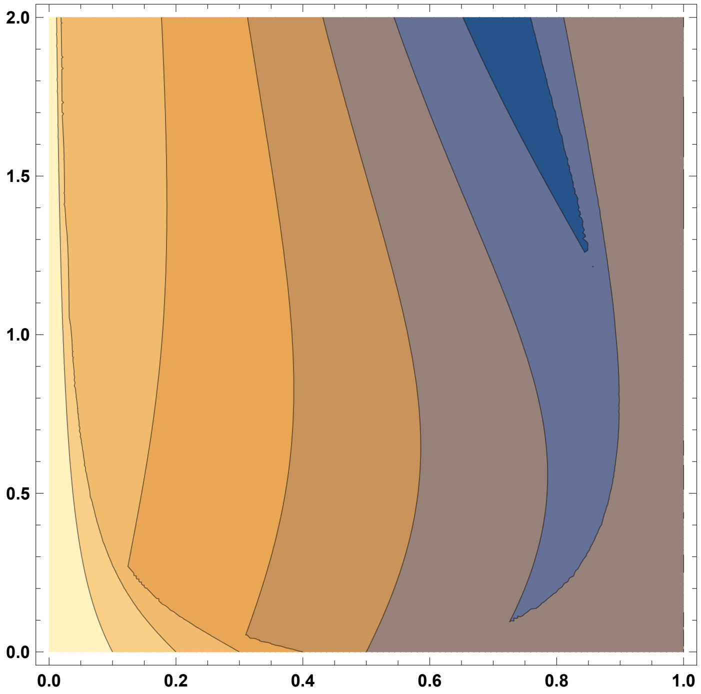

```
ContourPlot[Re[Evals[[2]]] /. σh → 2, {dh, 0, 1}, {Rv, 0, 2}]
```

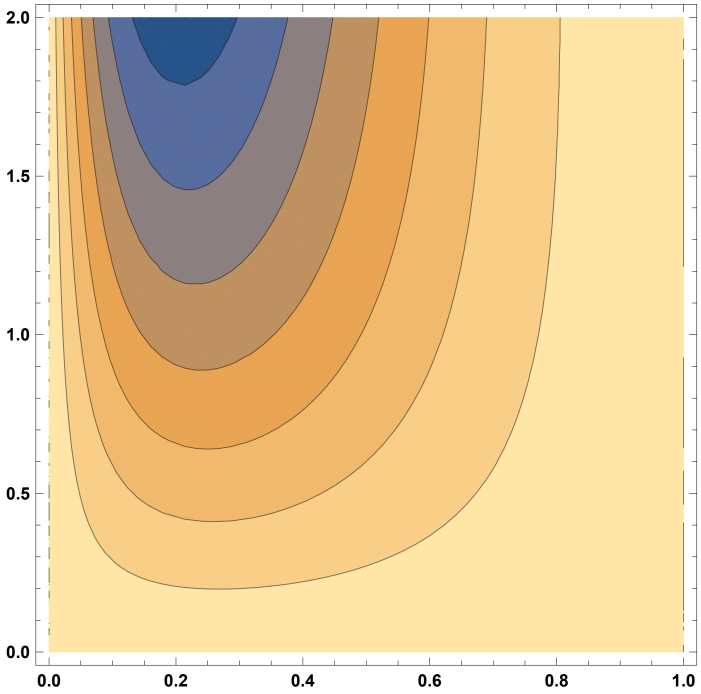

All values on the contour plots are negative, suggesting that the eigenvalues associated with the biologically relevant equilibrium have a negative real part. We conclude that the equilibrium is stable.

Biological implications / results

Seroprevalence with vaccine transmission and vaccination rate

Next, we calculate the seroprevalence that is achieved when a transmissible vaccine is used. Recall that the non-dimensionalization applied to the equations above rescales the state variables by the steady state density, $\frac{b}{d}$. As such, the seroprevalence is

In[64]:=
```
SeroTransSigND = FullSimplify[Simplify[(iv + v) /. SolTransSig[[1]]], Conds[[1]]]
```

Out[64]=
$$\frac{dh\,(-1+Rv)-\sigma h+\sqrt{-4\,dh^2\,Rv+(dh+dh\,Rv+\sigma h)^2}}{2\,dh\,Rv}$$

Convert back to dimensional parameters, except for Rv.

In[65]:=
```
SeroTransSig = FullSimplify[SeroTransSigND /. (dh → d/(d + δv)) /. (σh → σ/(d + δv)),
  Conds[[1]] && d > 0 && δv > 0 && Rv > 0]
```

Out[65]=
$$\frac{d\,(-1+Rv)-\sigma+\sqrt{d^2\,(-1+Rv)^2+2\,d\,(1+Rv)\,\sigma+\sigma^2}}{2\,d\,Rv}$$

Solve for the level of vaccine transmission necessary for herd-immunity ϕ

Solve for the critical vaccine transmission in terms of the vaccination rate σ

In[66]:=
```
CritRv = FullSimplify[Solve[SeroTransSig == ϕ , Rv]]
```

Out[66]= $\left\{\left\{Rv \to \frac{1}{1-\phi} - \frac{\sigma}{d\,\phi}\right\}\right\}$

Now, rewrite the level of transmission in terms of seroprevalence achieved with a nontransmissible vaccine, v = $\frac{\sigma}{\sigma + d}$.

In[67]:=
```
SigmaToSero = Solve[v == σ / (σ + d), σ]
```

Out[67]= $\left\{\left\{\sigma \to -\frac{d\,v}{-1+v}\right\}\right\}$

In[68]:=
```
FullSimplify[CritRv /. SigmaToSero]
```

Out[68]= $\left\{\left\{\left\{Rv \to \frac{1}{1-\phi} + \frac{v}{(-1+v)\,\phi}\right\}\right\}\right\}$

The above expression describes the critical level of vaccine transmission required, given that seroprevalence v is achieved with a non-transmissible vaccine. This is the red line plotted in both panels of Figure 4 in the main text.

Solve for the fractional reduction in baits required to maintain seroprevalence at ϕ

Earlier, we derived the relationship between the vaccination rate σ and the bait dynamics

In[69]:=
```
SigSolve = σ → c d ρ / (b c + d δ);
```

$$SigSolve = \sigma \to \frac{c\,d\,\rho}{b\,c + d\,\delta};$$

Because the effective vaccination rate σ is proportional to the rate at which vaccine baits are deposited (ρ), a fractional reduction in the vaccination rate is equivalent to a fractional reduction in the rate at which vaccine baits need to be deposited in the environment. If a non-transmissible vaccine is used, the resulting seroprevalence is

In[70]:=
$$SeroNoTransSig = \frac{\sigma}{\sigma + d};$$

Calculate the vaccination rate that is to maintain seroprevalence at ϕ with a nontransmissible vaccine:

In[71]:=
```
SigNoTrans = Solve[SeroNoTransSig == ϕ, σ]
```

Out[71]= $\left\{\left\{\sigma \to -\frac{d\,\phi}{-1+\phi}\right\}\right\}$

In contrast, when a transmissible vaccine is used, the seroprevalence (derived above) is

In[72]:=
$$SeroTransSig = \frac{d\,(-1+Rv) - \sigma + \sqrt{d^2\,(-1+Rv)^2 + 2\,d\,(1+Rv)\,\sigma + \sigma^2}}{2\,d\,Rv};$$

Calculate the vaccination rate that is required to maintain seroprevalence at ϕ using a transmissible vaccine:

In[73]:=
```
SigTrans = FullSimplify[Solve[SeroTransSig == ϕ, σ]]
```
Out[73]= $\left\{\left\{\sigma \to \frac{d\,\phi\,(-1+Rv-Rv\,\phi)}{-1+\phi}\right\}\right\}$

The fractional reduction in vaccination rate is

In[74]:=
```
FullSimplify[(1 - (σ /. SigTrans) / (σ /. SigNoTrans))]
```
Out[74]= $\{Rv - Rv\,\phi\}$

This is equation 14 in the main text.

PDE Model Analysis

Check that steady-state host density is $\frac{b}{d}$

The population density of the targeted host is described by the equation

$N_t = k\, N_{xx} + b - d\, N$, (M1)

Formal analysis of equation (1) requires both boundary conditions and an initial condition. The text imposed Neumann BC's for symmetry at the boundary points. For more generality and keeping with biological realism, we simply impose boundedness on solutions at +/- Infinity for this analysis, and let N(0,x) denote the initial condition. Setting the time derivative of (1) to 0 and integrating the resulting ODE yields the possible forms for steady-states as

```
DSolve[k Npop''[x] + b - d Npop[x] == 0, Npop[x], x]
```

$\left\{\left\{Npop[x] \to \frac{b}{d} + e^{\frac{\sqrt{d}\,x}{\sqrt{k}}} C[1] + e^{-\frac{\sqrt{d}\,x}{\sqrt{k}}} C[2]\right\}\right\}$

The biologically valid steady-state is that which is bounded at x = +/- Inf: $N^*(x) = \frac{b}{d}$. Next, we show that perturbations of Eq M1 around the unique bounded steady-state, $N^*(x) = \frac{b}{d}$, are stable. We write equation (1) in terms of the non-dimensional parameters L = $\frac{Sqrt[k]}{d}$, n = 1 + $N[t, x]$ / (b/d), $\chi = \frac{x}{L}$, and τ = d t. Here, n describes a perturbation around the steady-state $\frac{b}{d}$. Note that the usage of the parameter L here differs from that in the main text, where it denoted flight-line spacing. We investigate the case where the perturbation is spatially localized by imposing boundary conditions n[t,x] -> 0 as x -> +/- Inf. With this transform, system (1) becomes

$n_\tau = n_{\chi\chi}$ - n, (2)

with initial condition n(0,χ). In terms of the transformed state variable, the valid steady-state becomes $n^*(\chi) = 0$.

The fundamental solution of (2) is

```
ntotfund[χ_, τ_] := Exp[-τ] 1/Sqrt[4 Pi τ] Exp[-χ^2/(4 τ)];
```

which can be verified with the following:

```
Simplify[D[ntotfund[χ, τ], {τ, 1}] == D[ntotfund[χ, τ], {χ, 2}] - ntotfund[χ, τ]]
```

```
True
```

With initial condition $n_0[\chi]$, the solution to (2) is given by the convolution

```
Integrate[
 Integrate[n (0, χ) ntotfund[τ, y - χ], {χ, -Infinity, Infinity}], {τ, 0, t}]
```

which clearly limits to n[τ, χ] = 0 when τ -> Infinity, and n(0,χ) is biologically valid (bounded and non-negative). This implies that perturbations of equation (1) from the steady-state $\frac{b}{d}$ will always decay in time.
