# Supplementary figures and images for: A little goes a long way: Weak vaccine transmission facilitates oral vaccination campaigns against zoonotic pathogens
Source: PLoS Negl Trop Dis. 2019 Mar 8;13(3):e0007251. doi: 10.1371/journal.pntd.0007251 (PMC6426267; doi:10.1371/journal.pntd.0007251)

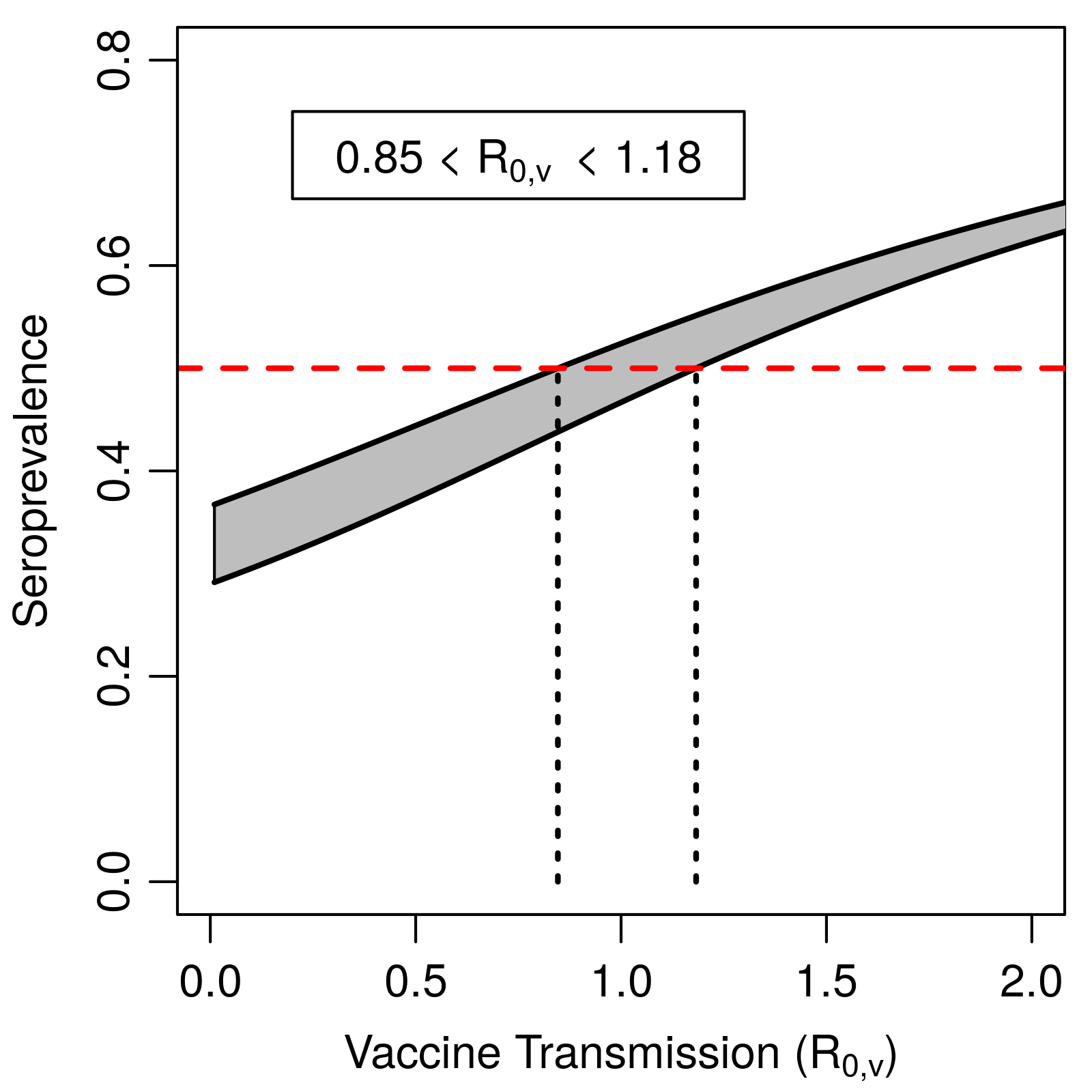

Supplement: S4 Appendix — This zip file contains R scripts that simulate and plot the numerical results presented in the manuscript. (ZIP) [file pntd.0007251.s004.zip › S4_RCode/Figure_1/Figure_1.tif]

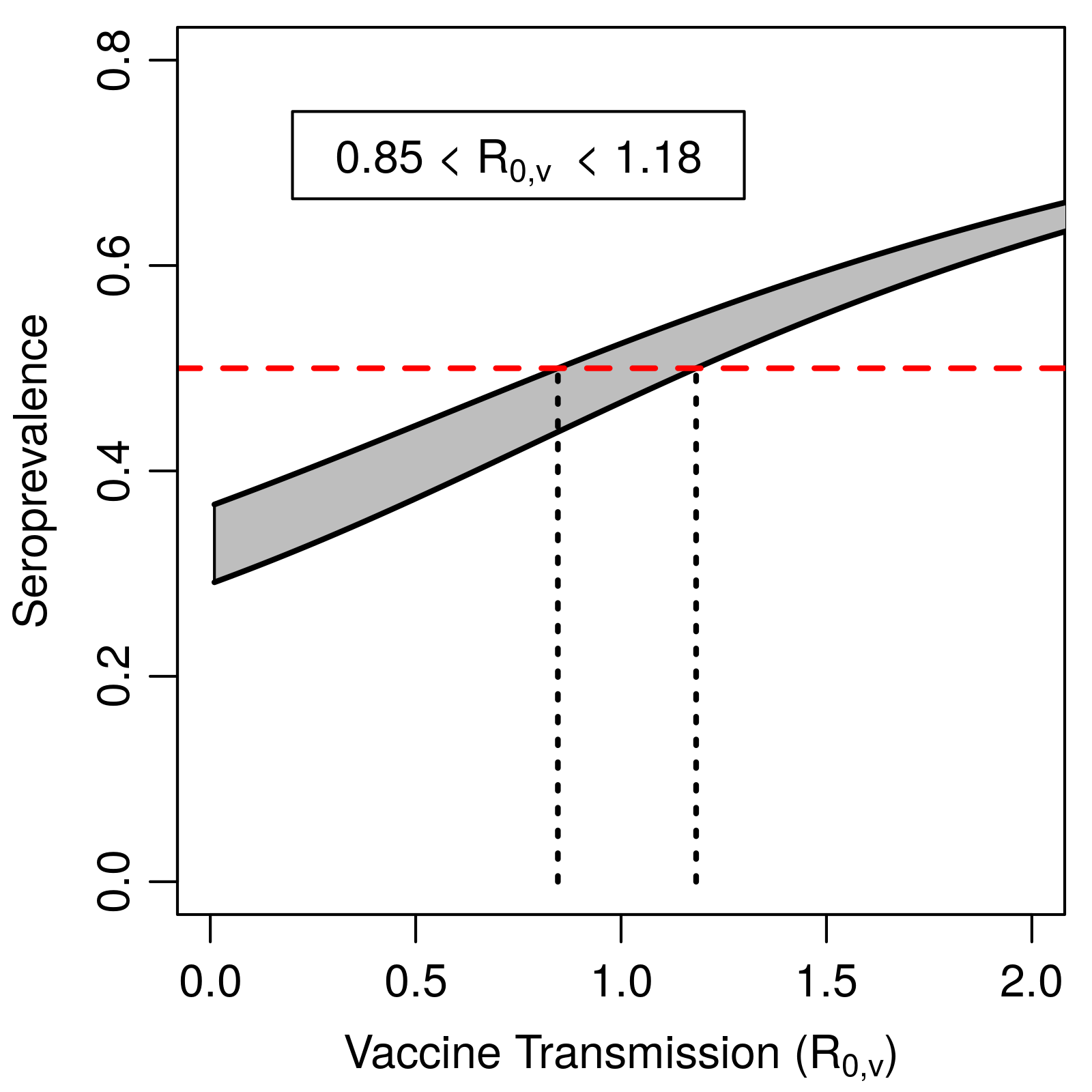

Supplement: S4 Appendix — This zip file contains R scripts that simulate and plot the numerical results presented in the manuscript. (ZIP) [file pntd.0007251.s004.zip › S4_RCode/Figure_1/Figure_1.png]

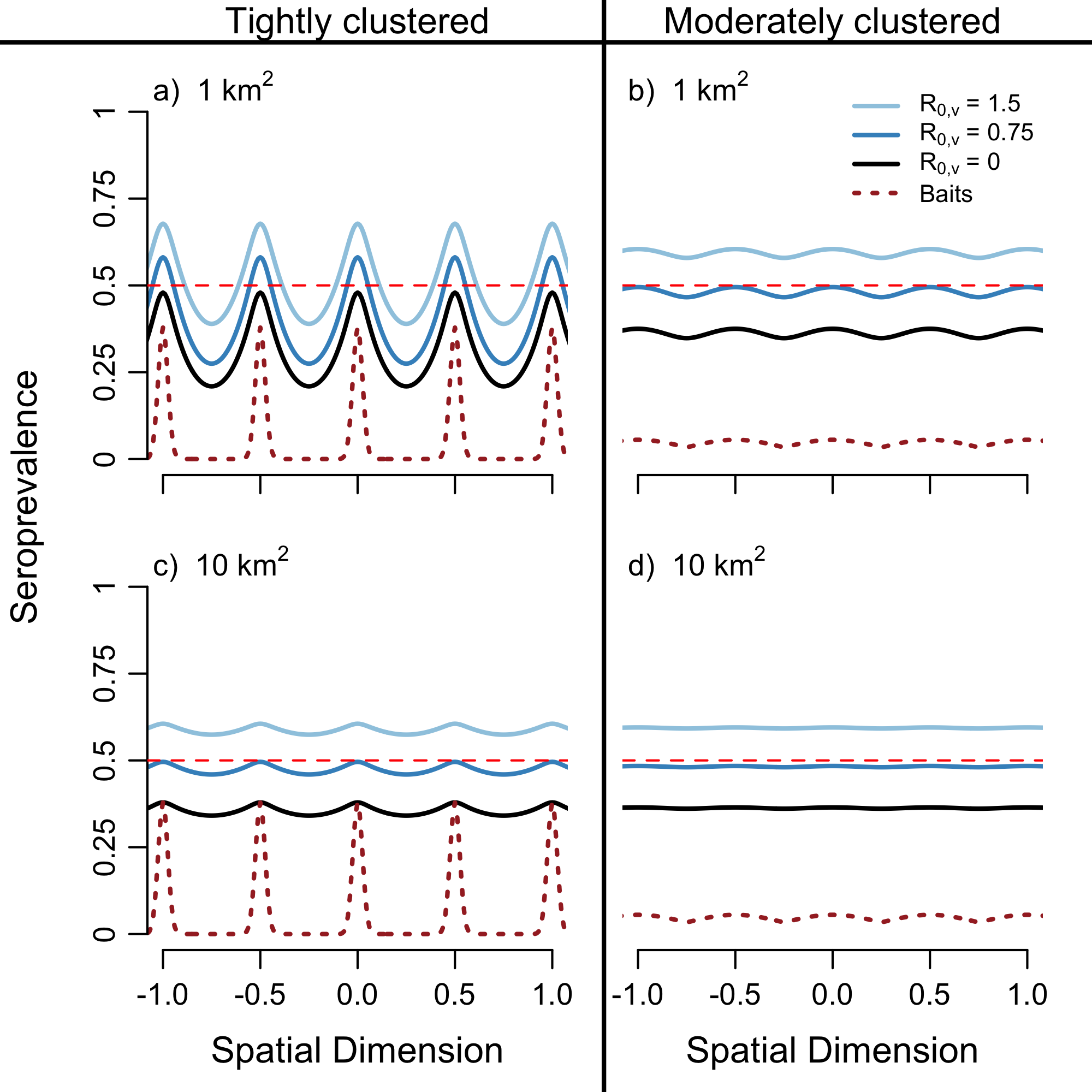

Supplement: S4 Appendix — This zip file contains R scripts that simulate and plot the numerical results presented in the manuscript. (ZIP) [file pntd.0007251.s004.zip › S4_RCode/Figure_2/Figure_2.png]

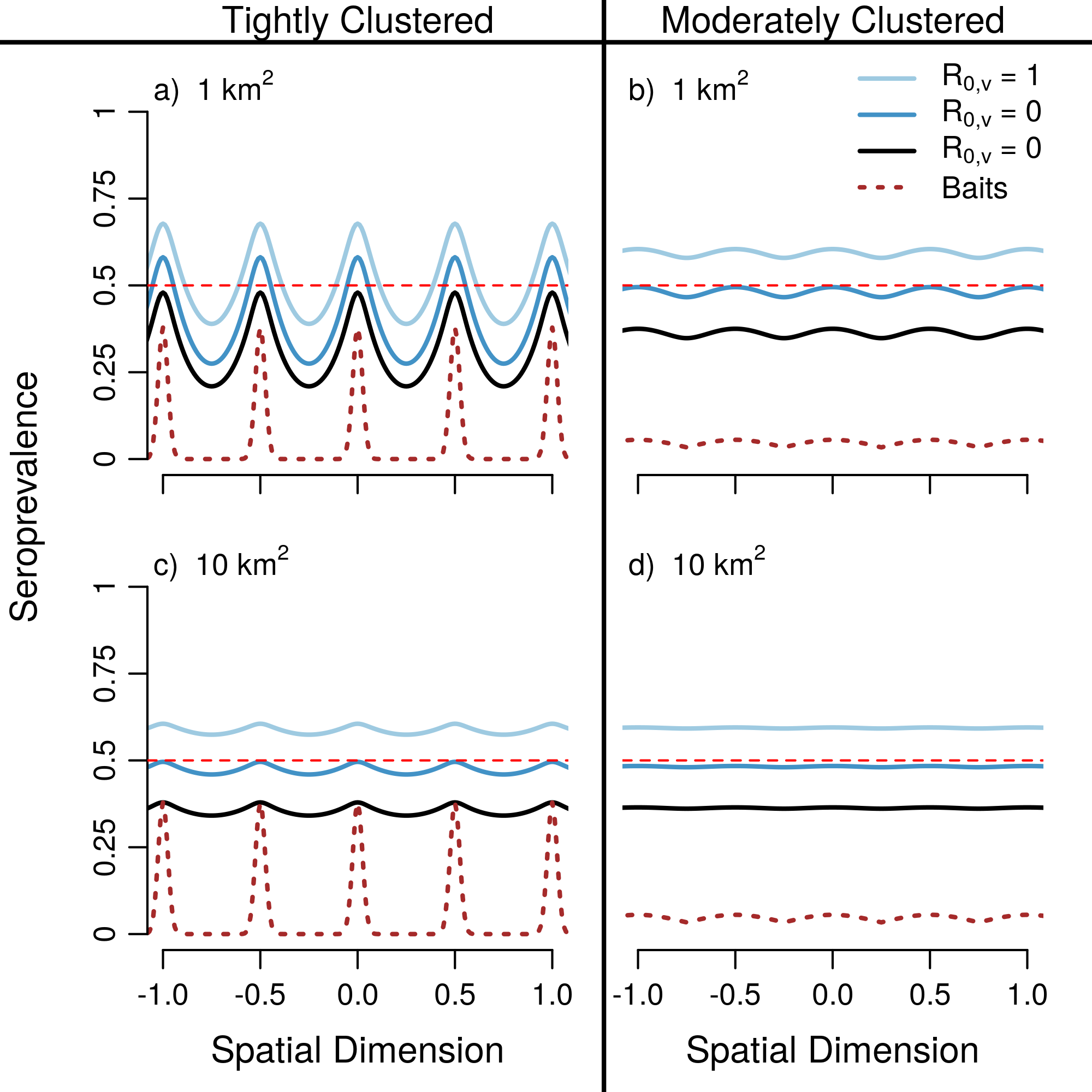

Supplement: S4 Appendix — This zip file contains R scripts that simulate and plot the numerical results presented in the manuscript. (ZIP) [file pntd.0007251.s004.zip › S4_RCode/Figure_2/Figure_2.tif]

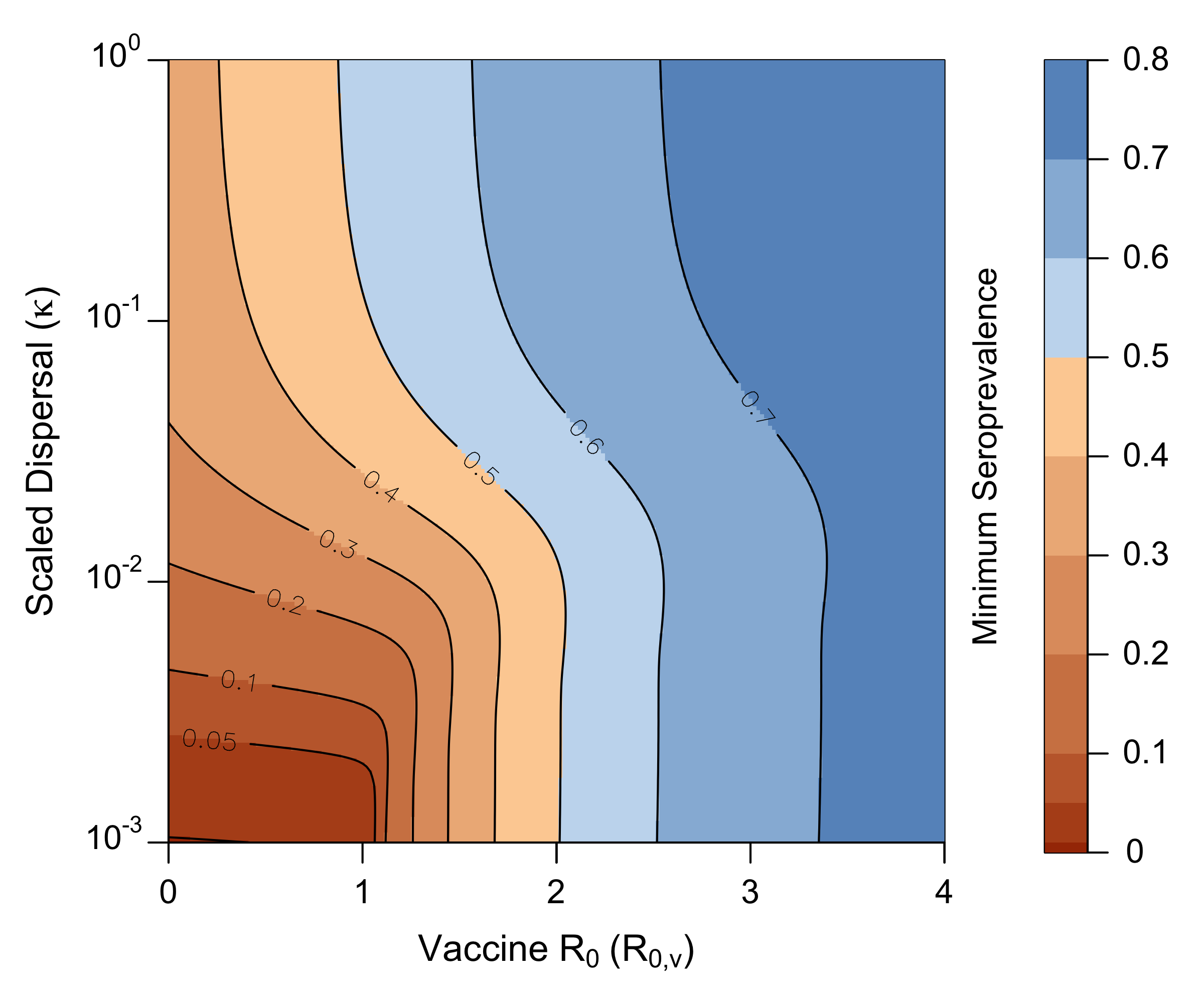

Supplement: S4 Appendix — This zip file contains R scripts that simulate and plot the numerical results presented in the manuscript. (ZIP) [file pntd.0007251.s004.zip › S4_RCode/Figure_3/Fig_3/Figure_3.png]

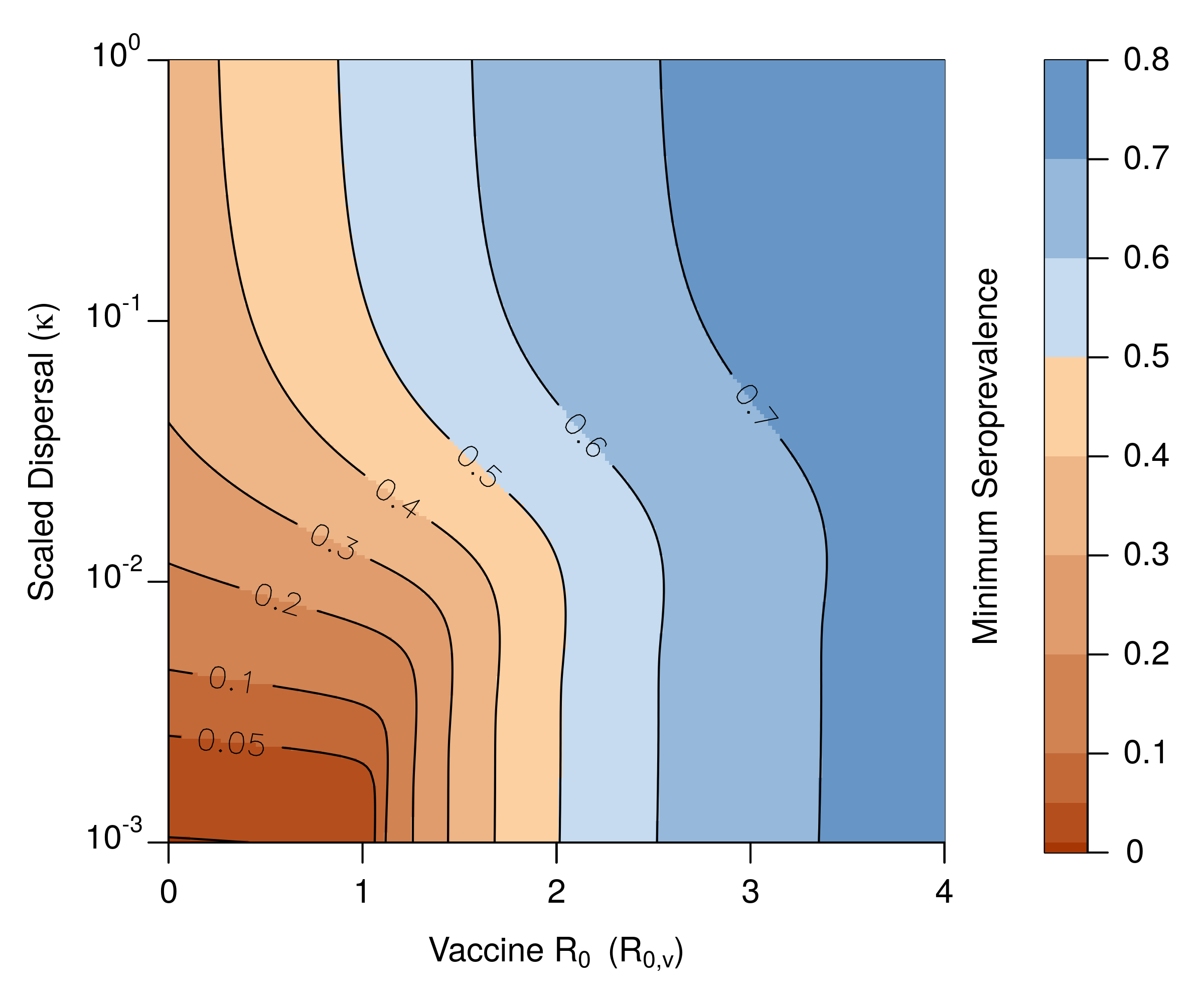

Supplement: S4 Appendix — This zip file contains R scripts that simulate and plot the numerical results presented in the manuscript. (ZIP) [file pntd.0007251.s004.zip › S4_RCode/Figure_3/Fig_3/Figure_3.tif]

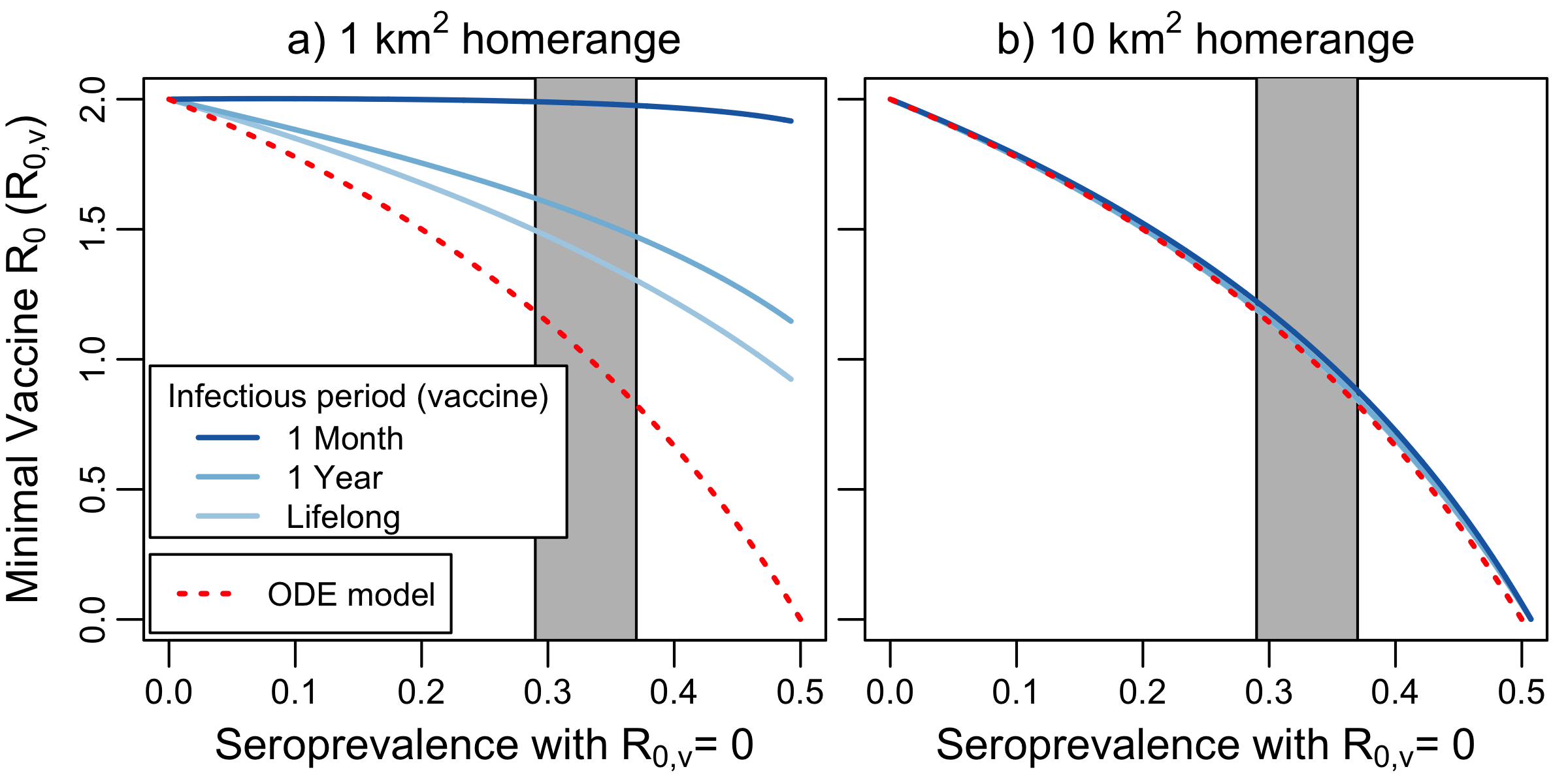

Supplement: S4 Appendix — This zip file contains R scripts that simulate and plot the numerical results presented in the manuscript. (ZIP) [file pntd.0007251.s004.zip › S4_RCode/Figure_4/Fig_4/Figure_4.png]

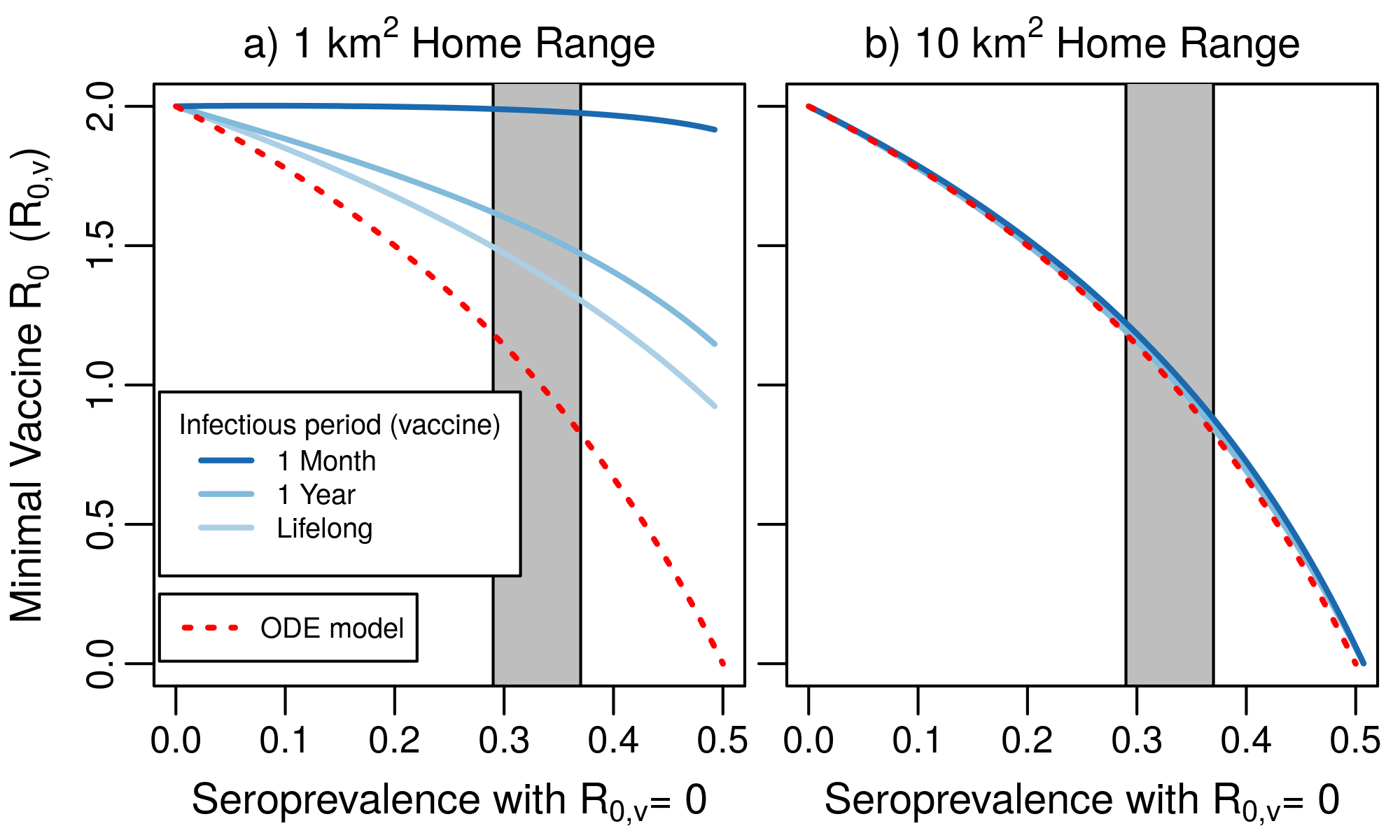

Supplement: S4 Appendix — This zip file contains R scripts that simulate and plot the numerical results presented in the manuscript. (ZIP) [file pntd.0007251.s004.zip › S4_RCode/Figure_4/Fig_4/Figure_4.tif]

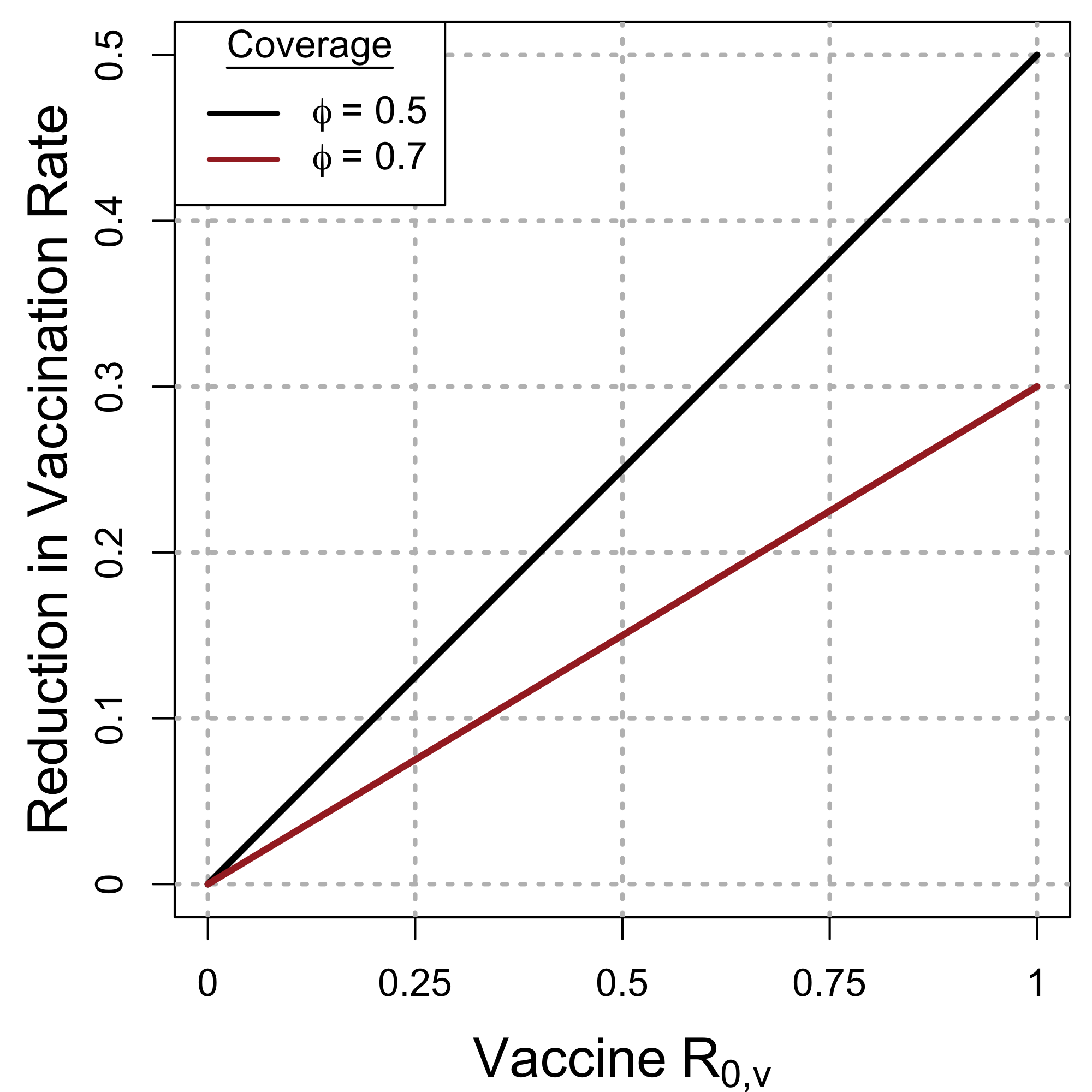

Supplement: S4 Appendix — This zip file contains R scripts that simulate and plot the numerical results presented in the manuscript. (ZIP) [file pntd.0007251.s004.zip › S4_RCode/Figure_5/Figure_5.png]

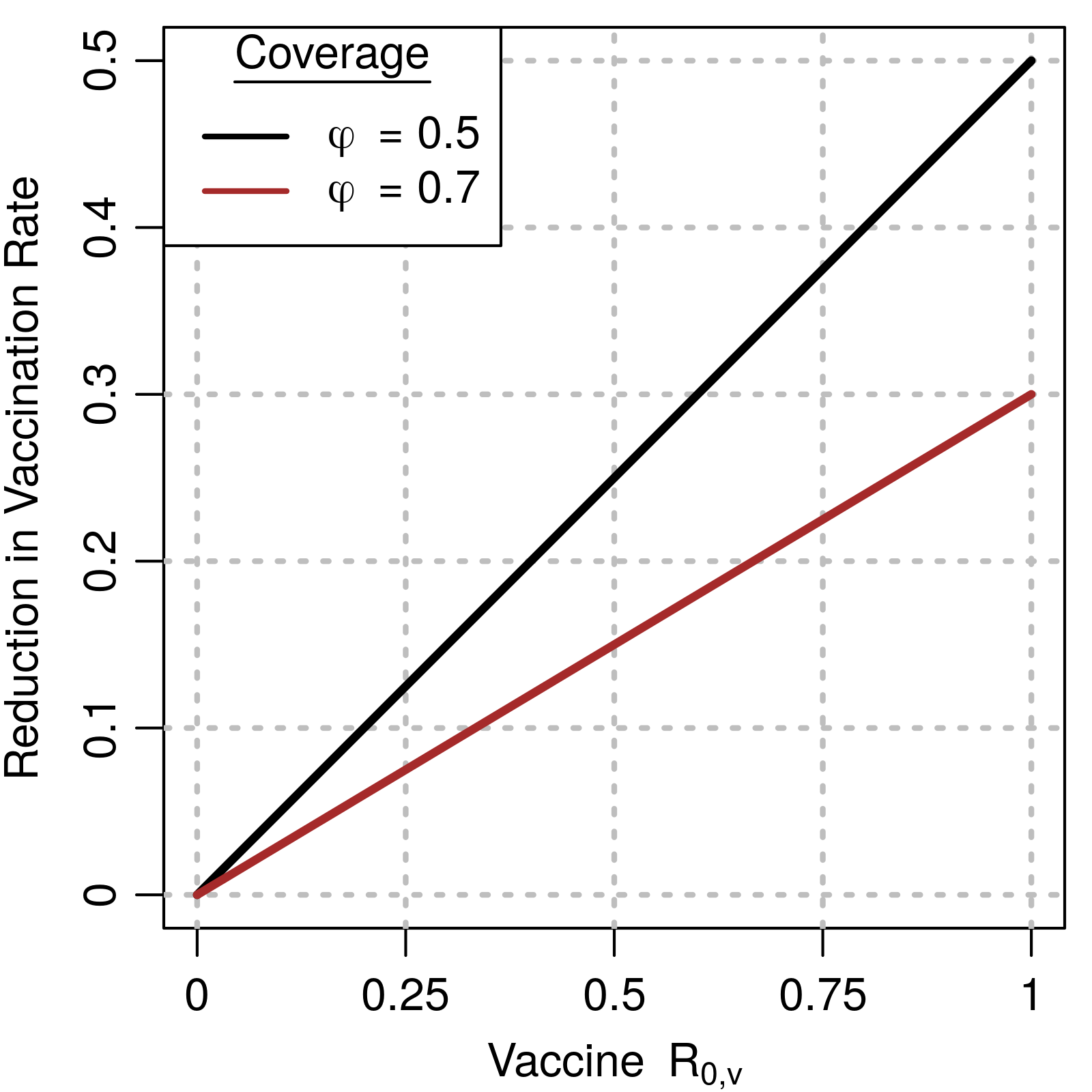

Supplement: S4 Appendix — This zip file contains R scripts that simulate and plot the numerical results presented in the manuscript. (ZIP) [file pntd.0007251.s004.zip › S4_RCode/Figure_5/Figure_5.tif]

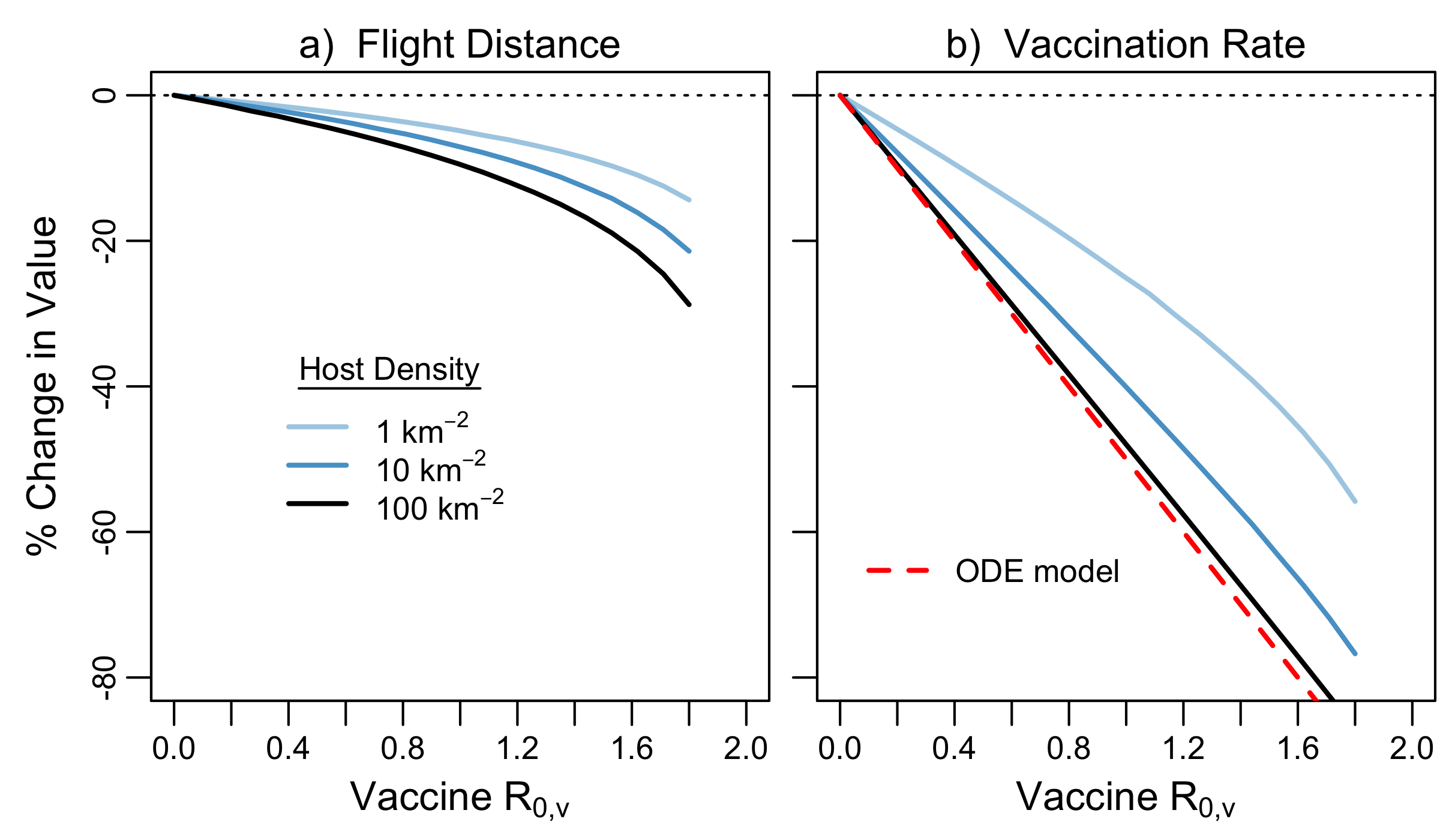

Supplement: S4 Appendix — This zip file contains R scripts that simulate and plot the numerical results presented in the manuscript. (ZIP) [file pntd.0007251.s004.zip › S4_RCode/Figure_6/Fig_6/Figure_6.png]

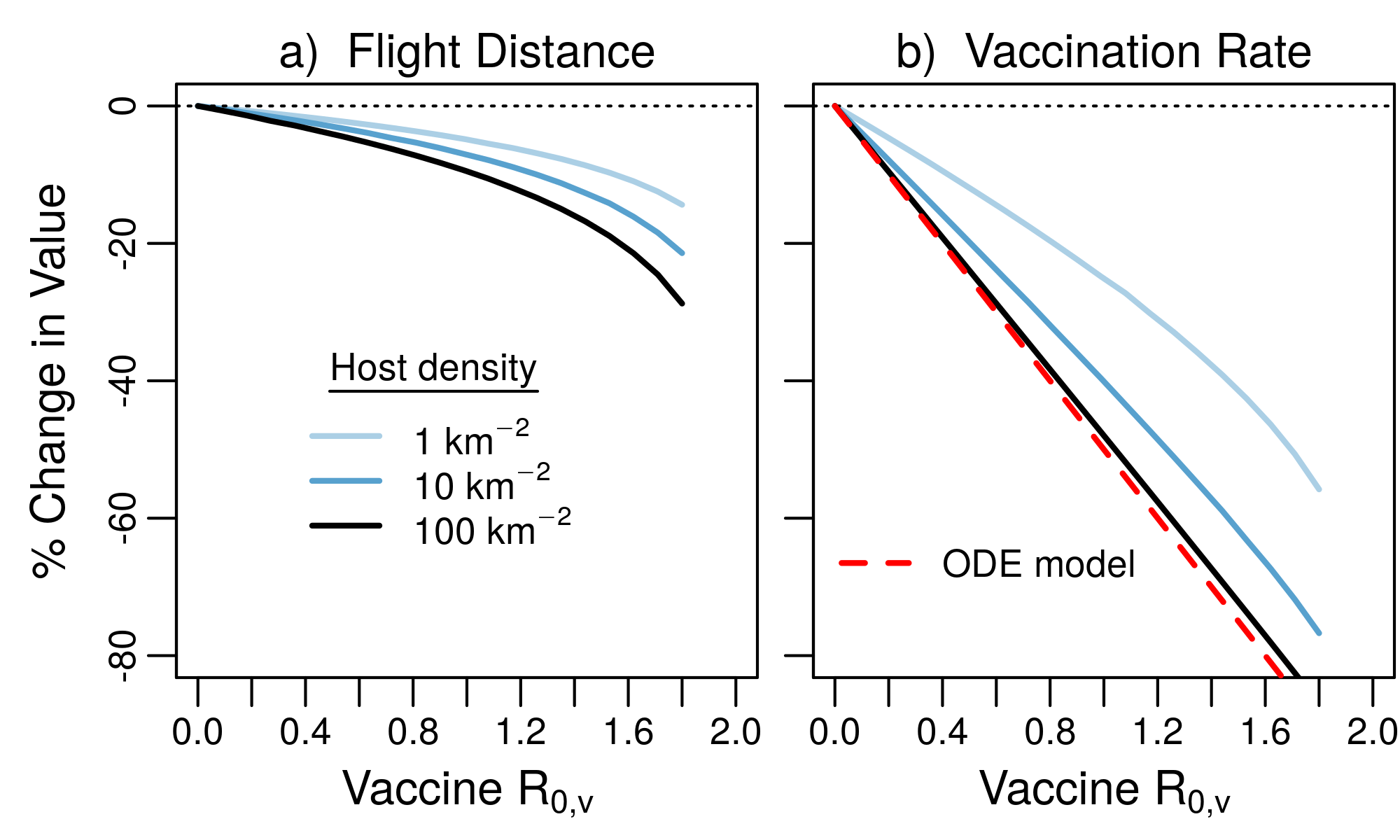

Supplement: S4 Appendix — This zip file contains R scripts that simulate and plot the numerical results presented in the manuscript. (ZIP) [file pntd.0007251.s004.zip › S4_RCode/Figure_6/Fig_6/Figure_6.tif]

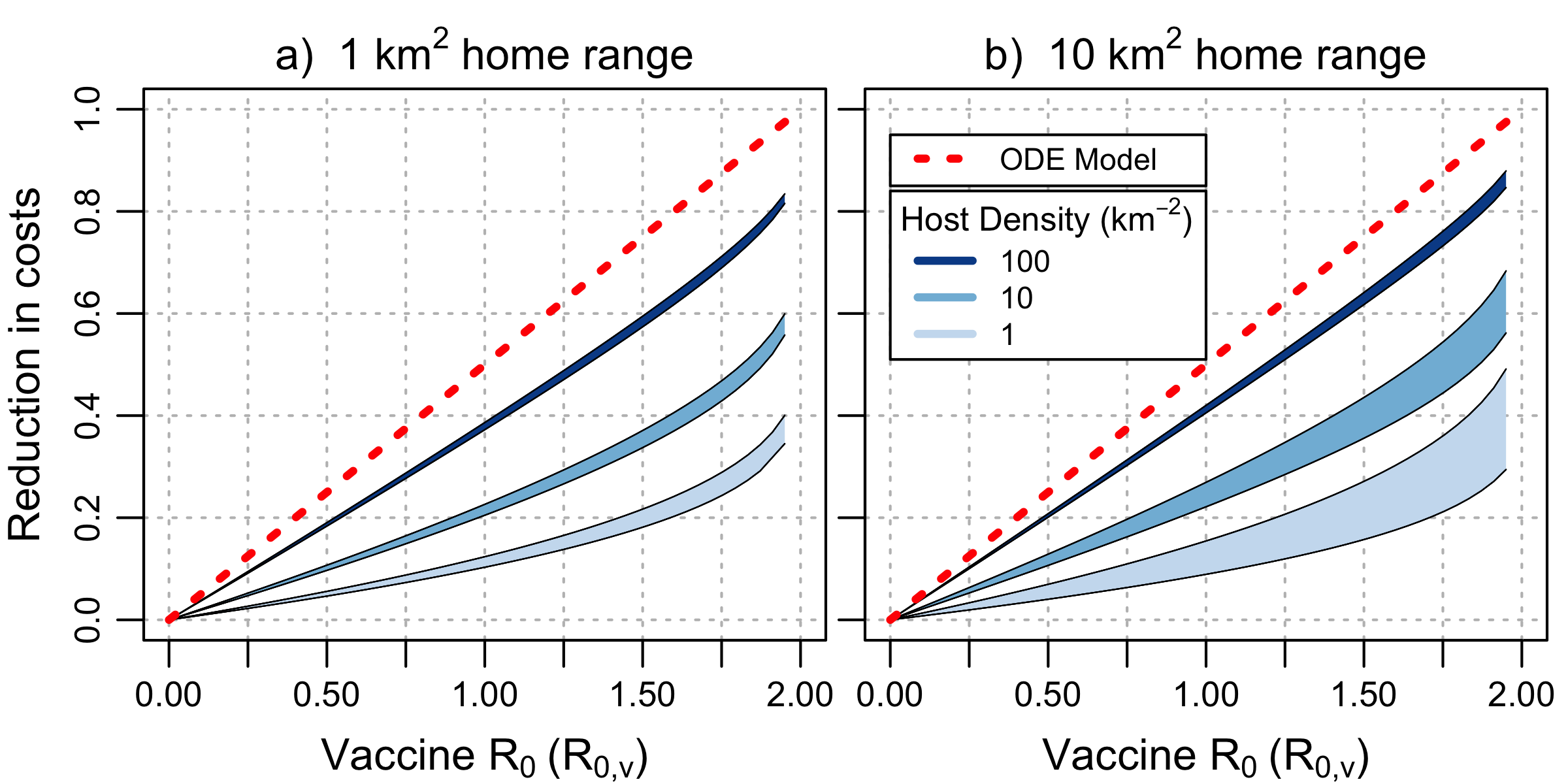

Supplement: S4 Appendix — This zip file contains R scripts that simulate and plot the numerical results presented in the manuscript. (ZIP) [file pntd.0007251.s004.zip › S4_RCode/Figure_7/Fig_7/Figure_7.png]

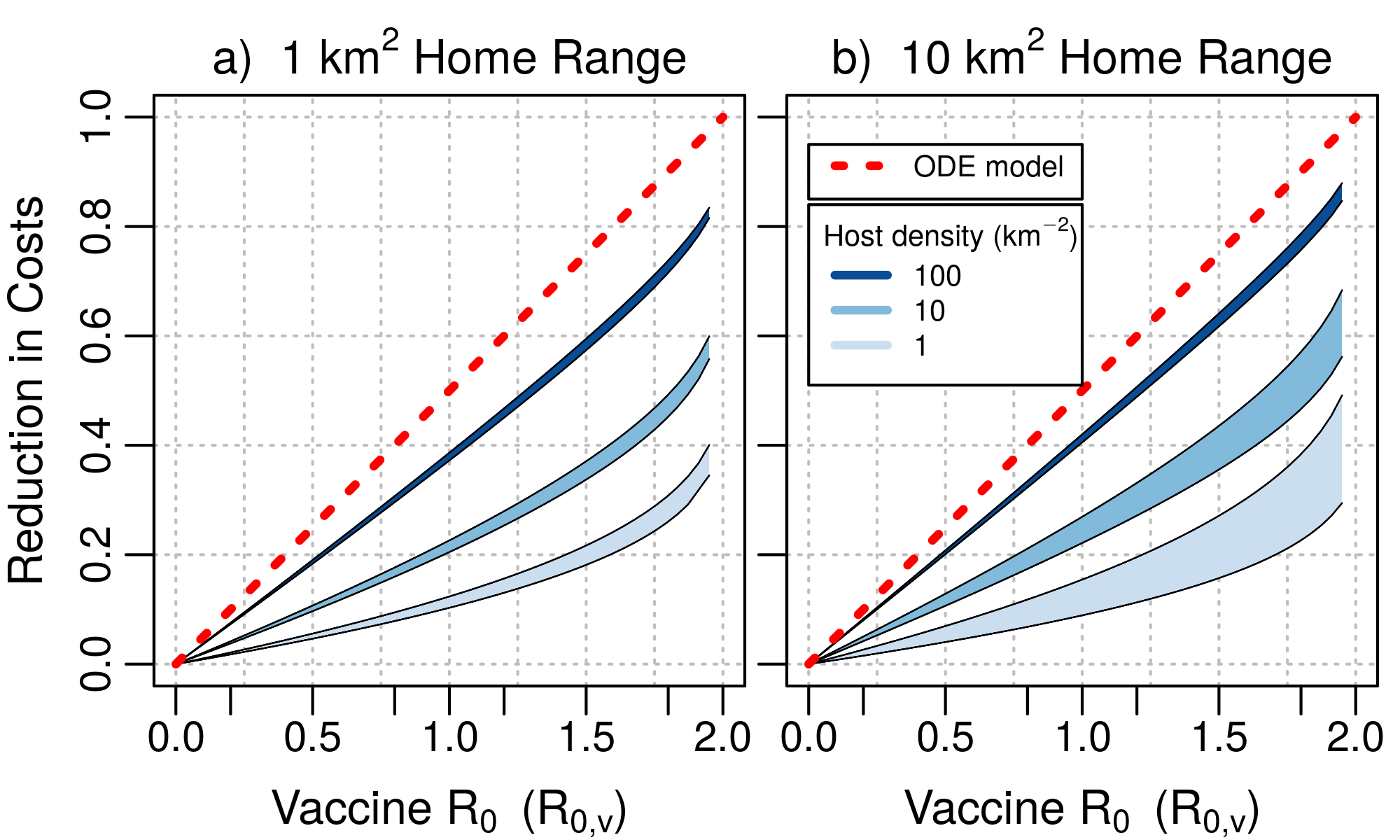

Supplement: S4 Appendix — This zip file contains R scripts that simulate and plot the numerical results presented in the manuscript. (ZIP) [file pntd.0007251.s004.zip › S4_RCode/Figure_7/Fig_7/Figure_7.tif]

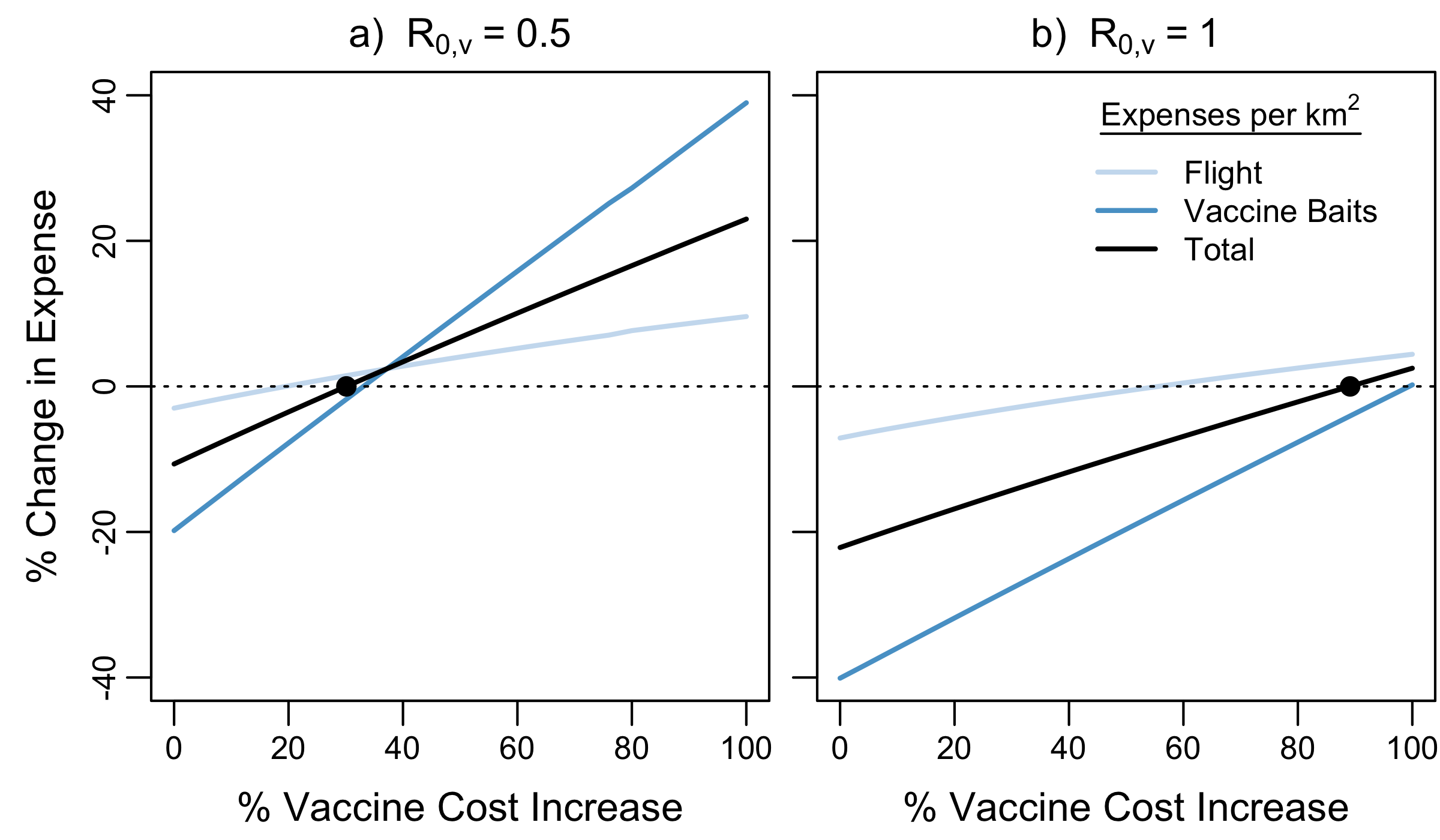

Supplement: S4 Appendix — This zip file contains R scripts that simulate and plot the numerical results presented in the manuscript. (ZIP) [file pntd.0007251.s004.zip › S4_RCode/Figure_8/Fig_8/Figure_8.png]

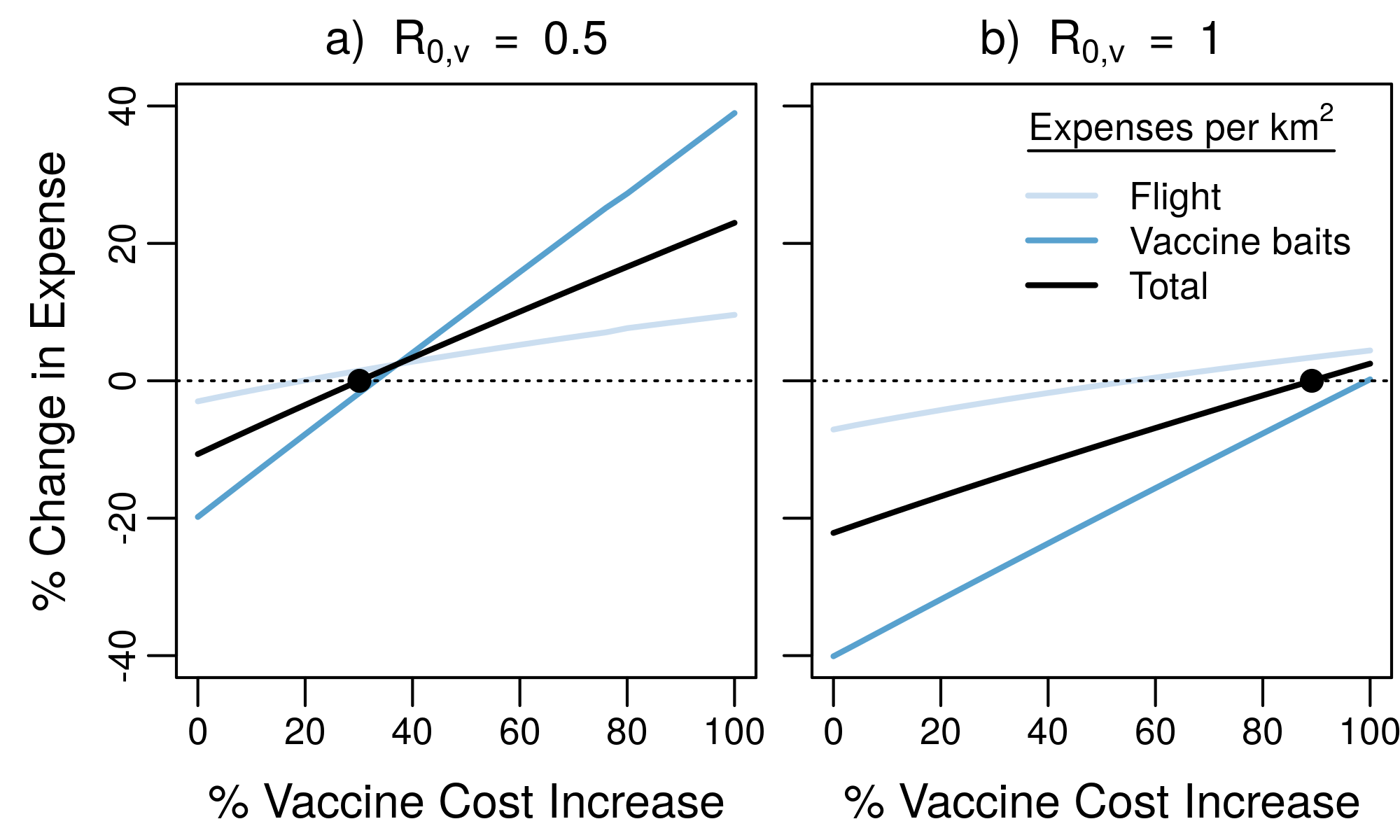

Supplement: S4 Appendix — This zip file contains R scripts that simulate and plot the numerical results presented in the manuscript. (ZIP) [file pntd.0007251.s004.zip › S4_RCode/Figure_8/Fig_8/Figure_8.tif]

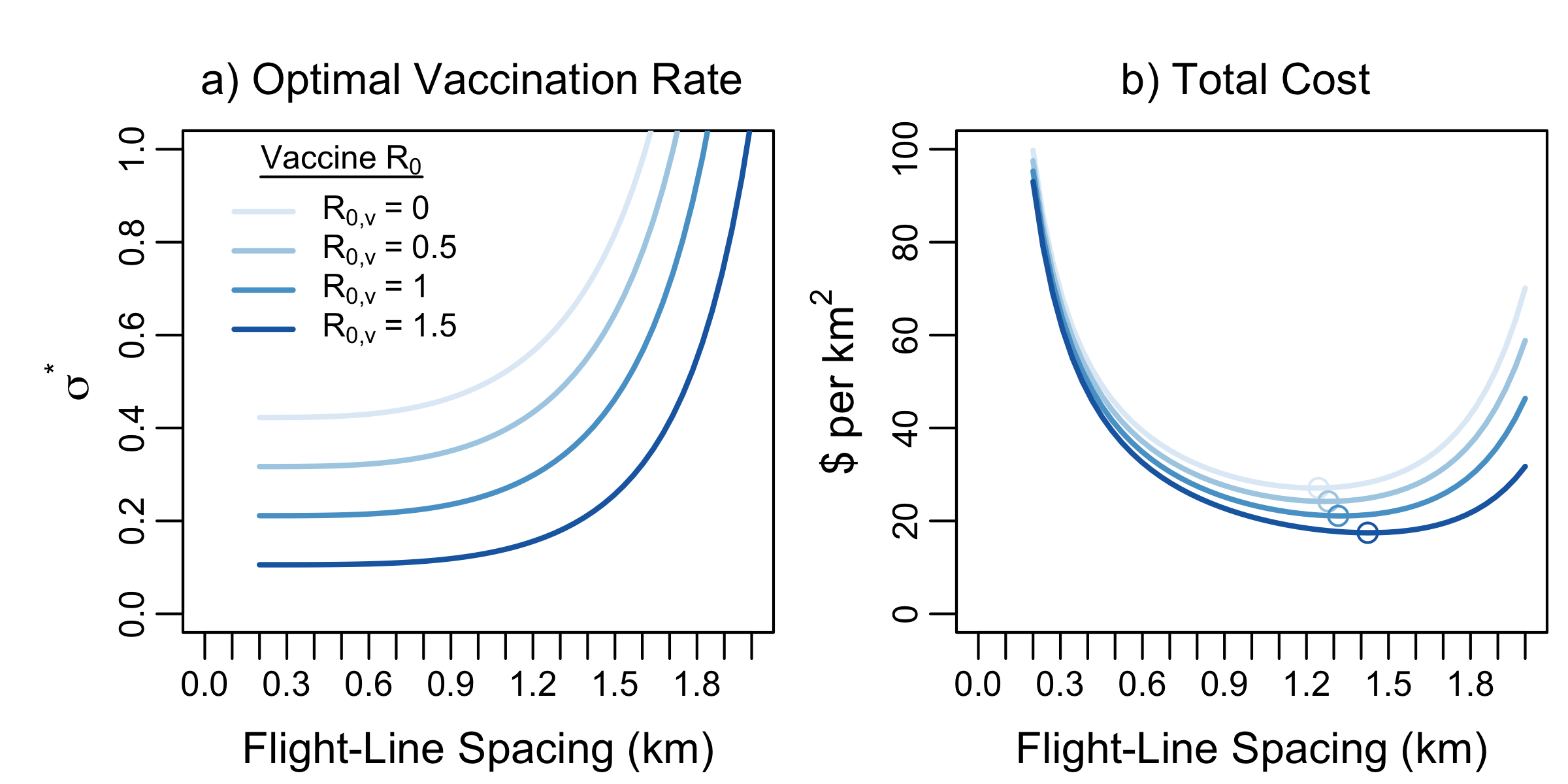

Supplement: S4 Appendix — This zip file contains R scripts that simulate and plot the numerical results presented in the manuscript. (ZIP) [file pntd.0007251.s004.zip › S4_RCode/Figure_S1/Fig_S1/Figure_S1.png]

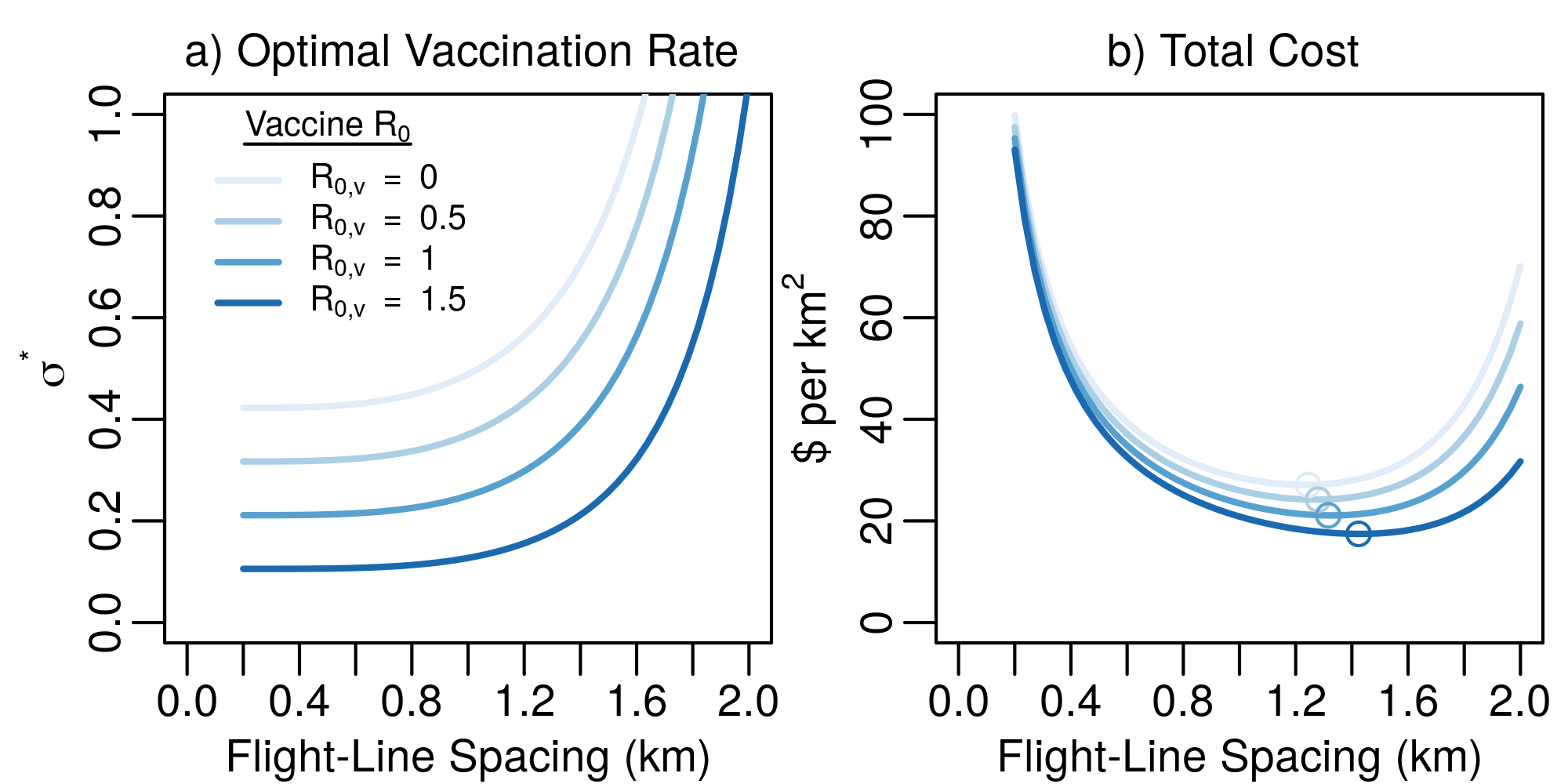

Supplement: S4 Appendix — This zip file contains R scripts that simulate and plot the numerical results presented in the manuscript. (ZIP) [file pntd.0007251.s004.zip › S4_RCode/Figure_S1/Fig_S1/Figure_S1.tif]

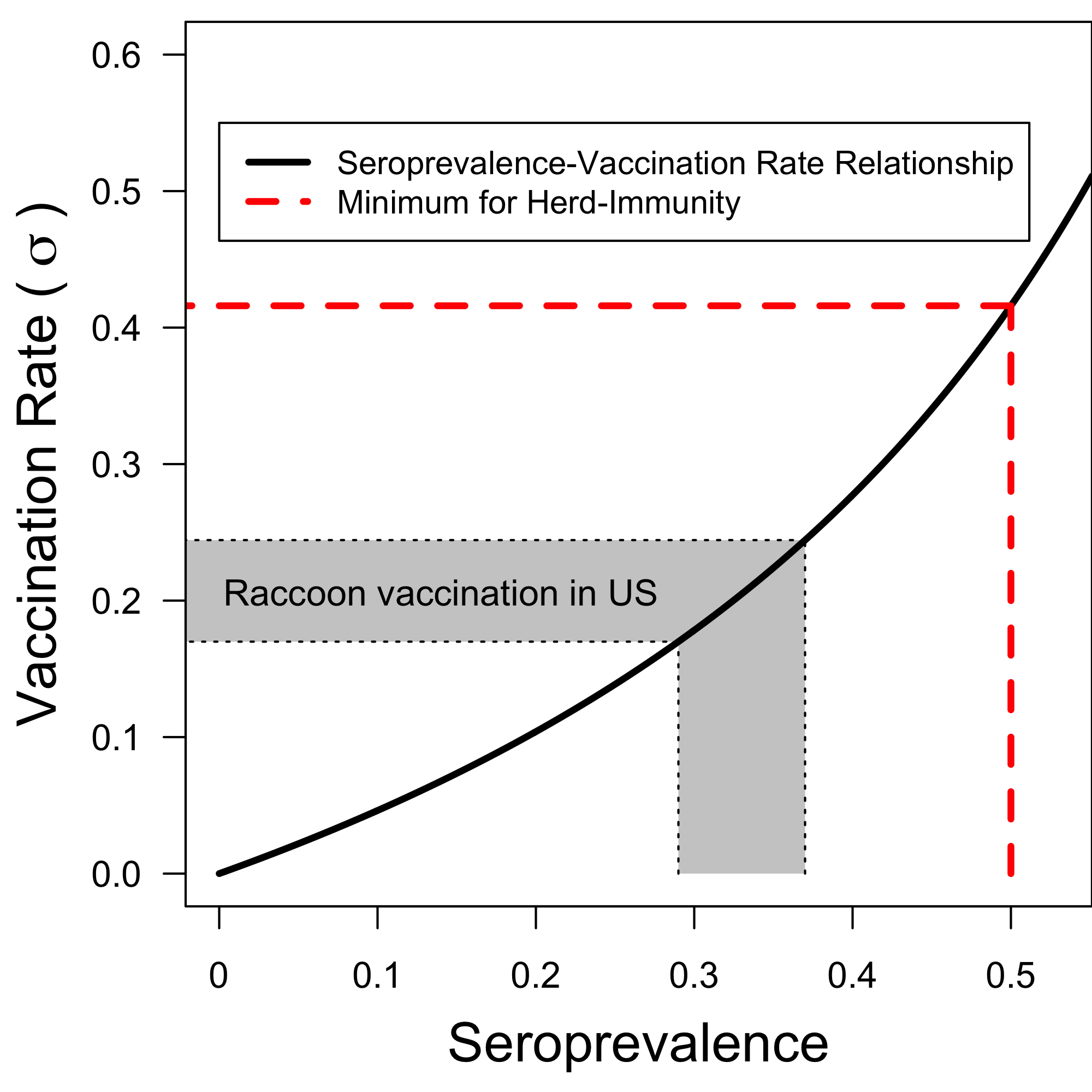

Supplement: S4 Appendix — This zip file contains R scripts that simulate and plot the numerical results presented in the manuscript. (ZIP) [file pntd.0007251.s004.zip › S4_RCode/Figure_S1_1/Figure_S1_1.png]

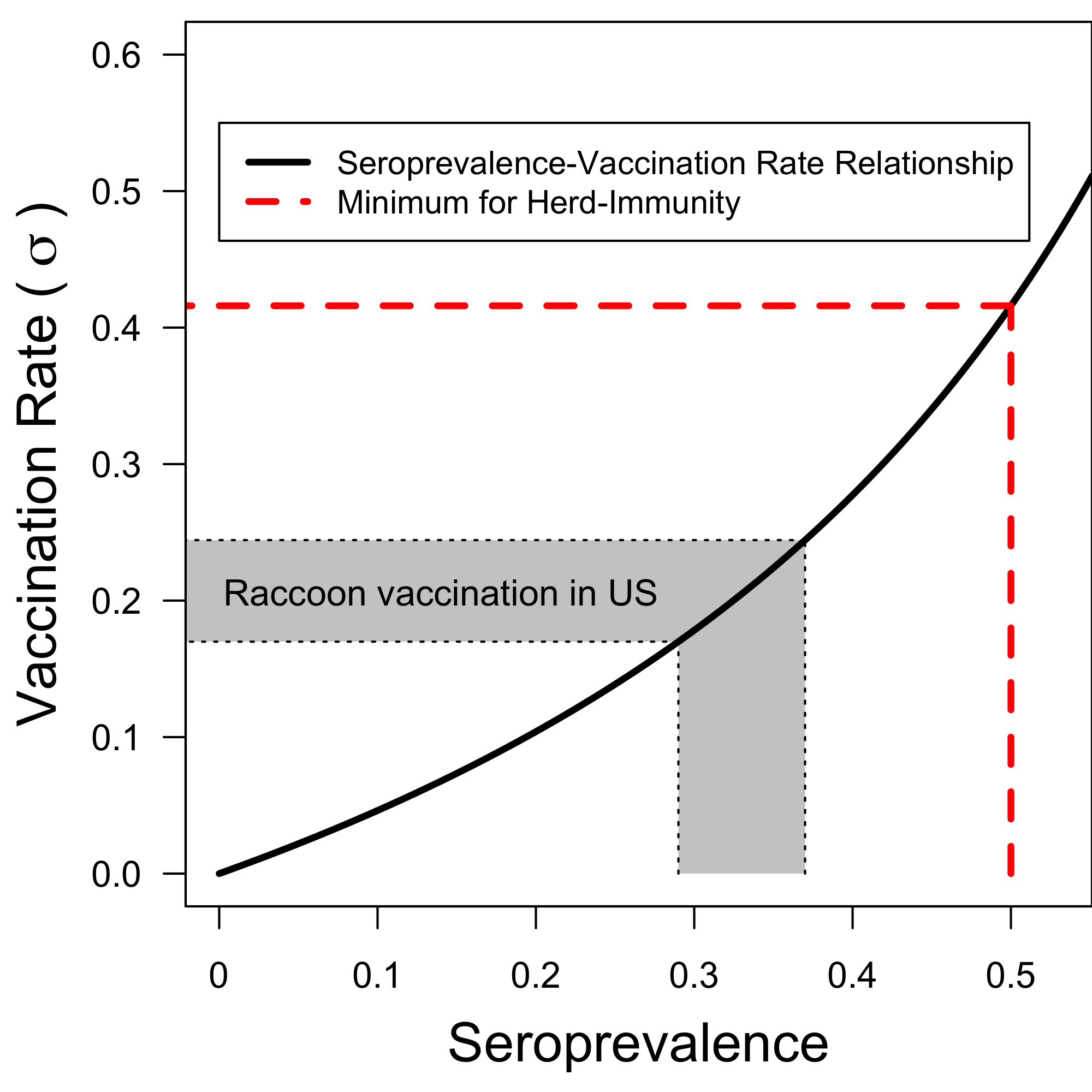

Supplement: S4 Appendix — This zip file contains R scripts that simulate and plot the numerical results presented in the manuscript. (ZIP) [file pntd.0007251.s004.zip › S4_RCode/Figure_S1_1/Figure_S1_1.tif]

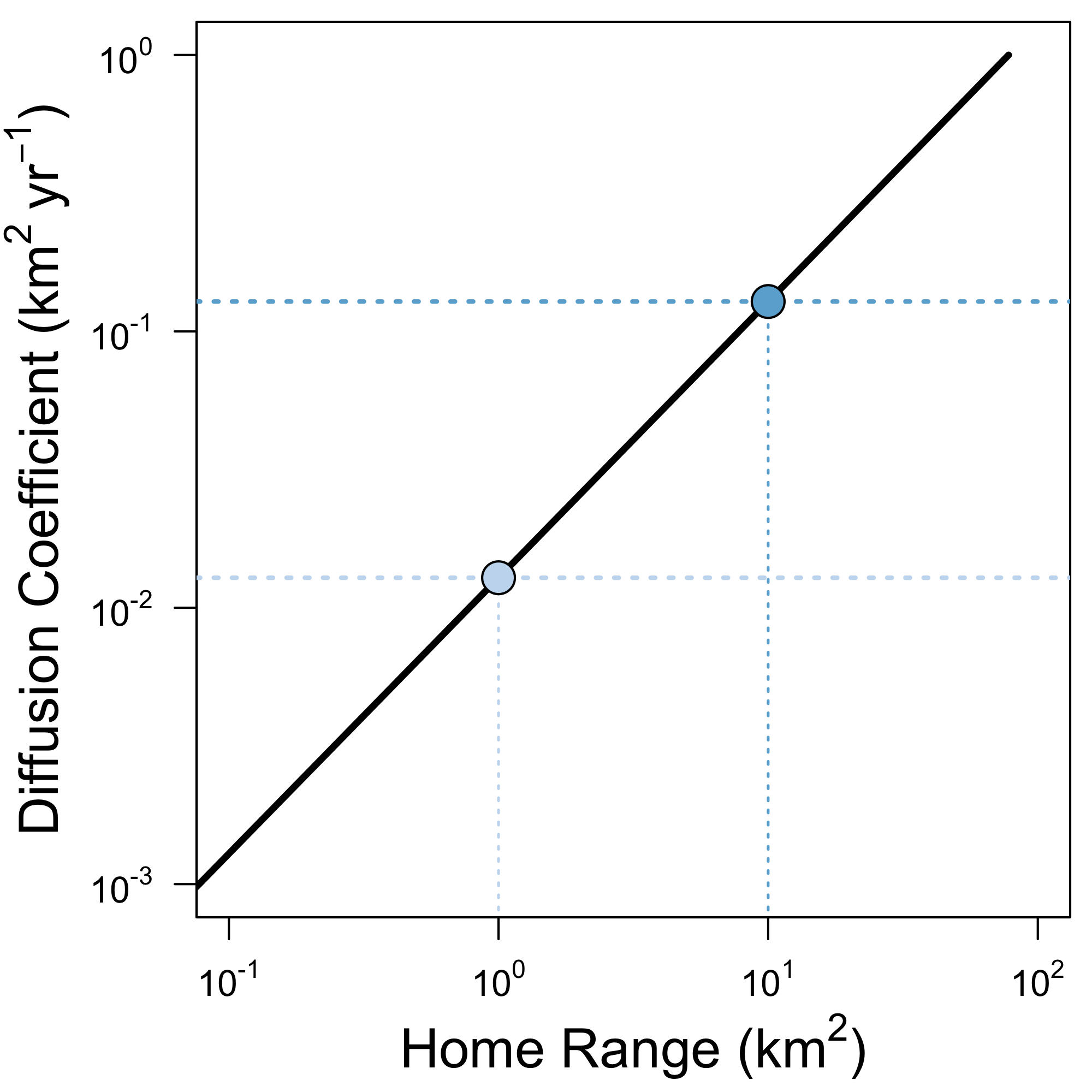

Supplement: S4 Appendix — This zip file contains R scripts that simulate and plot the numerical results presented in the manuscript. (ZIP) [file pntd.0007251.s004.zip › S4_RCode/Figure_S1_2/Figure_S1_2.png]

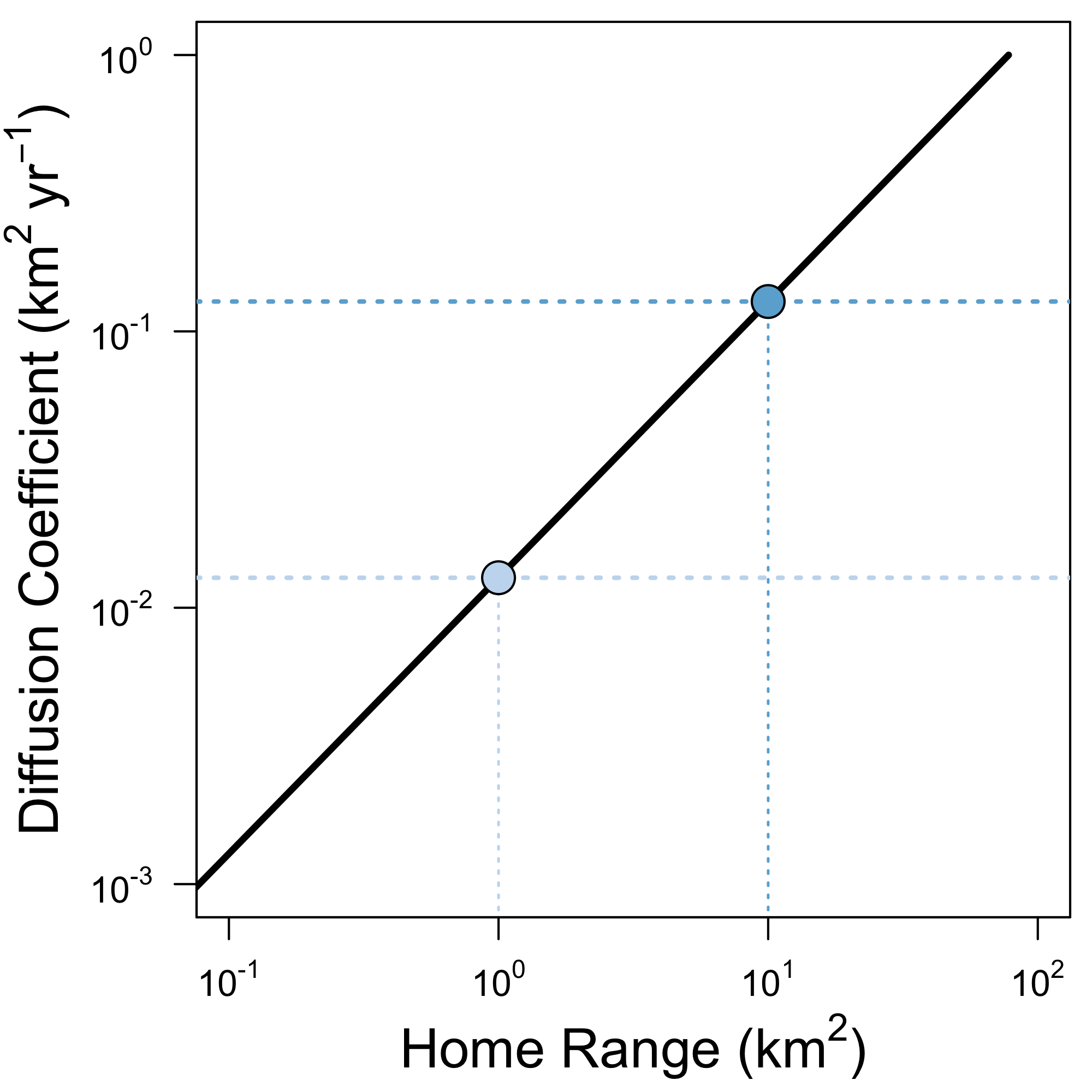

Supplement: S4 Appendix — This zip file contains R scripts that simulate and plot the numerical results presented in the manuscript. (ZIP) [file pntd.0007251.s004.zip › S4_RCode/Figure_S1_2/Figure_S1_2.tif]

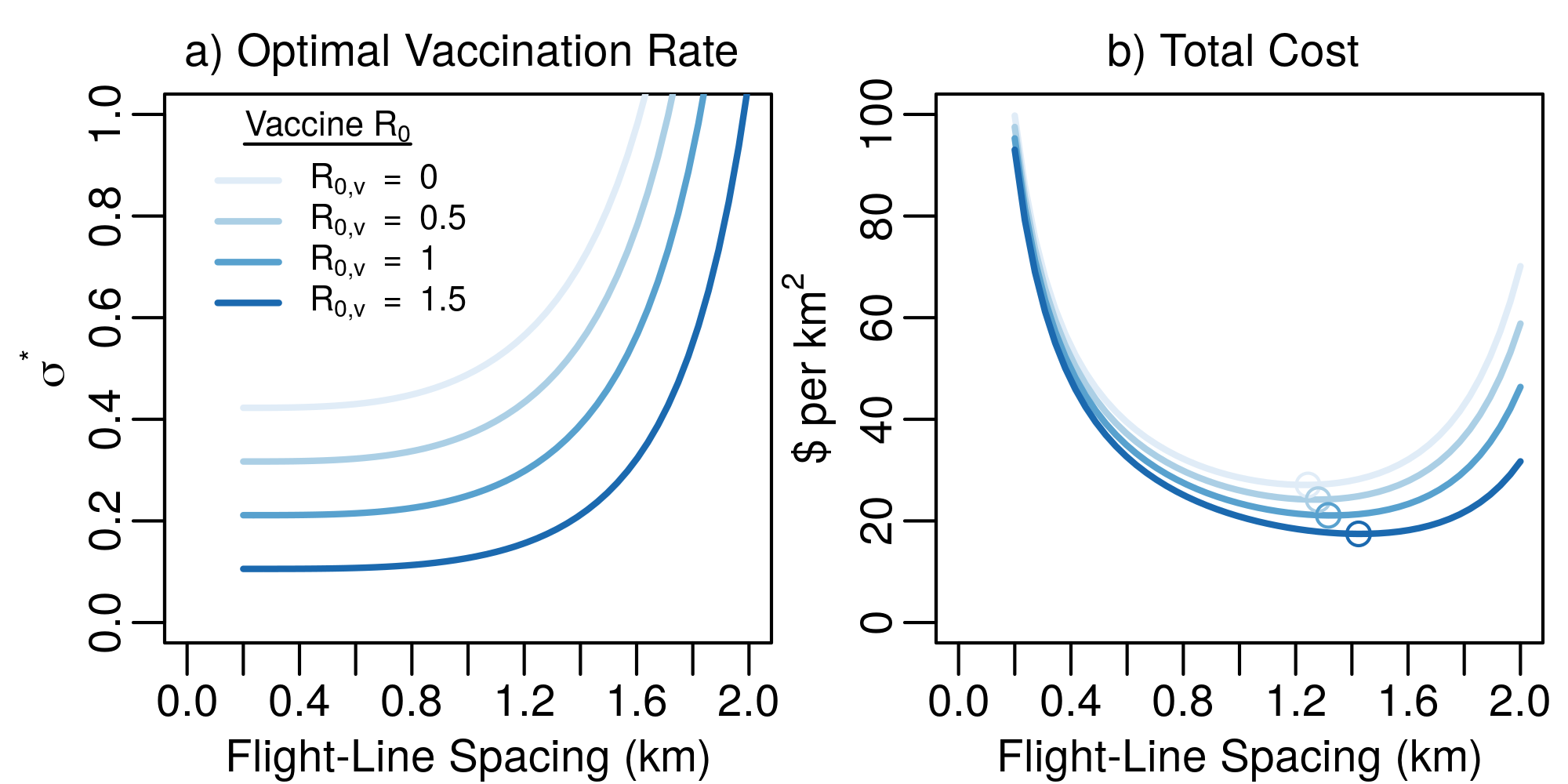

Supplement: S1 Fig — Panel (a) shows the minimal vaccination rate, σ*, that is necessary to hold seroprevalence at ϕ = 0.5 for each flight-line spacing. Panel (b) shows the resulting costs of the strategies in panel (a). Dots indicate the cost-minimizing strategy. Other parameters: d = 0.416 yr−1, δv = 12 yr−1, k = 0.1 km2 yr−1, ξ = 0.25 km, Cf = 18.16 km−1, Cb = 2.12. (TIF) [file pntd.0007251.s005.tif]
